# A pan‐metazoan concept for adult stem cells: the wobbling Penrose landscape

**DOI:** 10.1111/brv.12801

**Published:** 2021-10-06

**Authors:** Baruch Rinkevich, Loriano Ballarin, Pedro Martinez, Ildiko Somorjai, Oshrat Ben‐Hamo, Ilya Borisenko, Eugene Berezikov, Alexander Ereskovsky, Eve Gazave, Denis Khnykin, Lucia Manni, Olga Petukhova, Amalia Rosner, Eric Röttinger, Antonietta Spagnuolo, Michela Sugni, Stefano Tiozzo, Bert Hobmayer

**Affiliations:** ^1^ Israel Oceanographic & Limnological Research National Institute of Oceanography POB 9753, Tel Shikmona Haifa 3109701 Israel; ^2^ Department of Biology University of Padova Via Ugo Bassi 58/B Padova 35121 Italy; ^3^ Departament de Genètica, Microbiologia i Estadística Universitat de Barcelona Av. Diagonal 643 Barcelona 08028 Spain; ^4^ Institut Català de Recerca i Estudis Avançats (ICREA) Passeig Lluís Companys 23 Barcelona 08010 Spain; ^5^ School of Biology University of St Andrews St Andrews, Fife KY16 9ST, Scotland UK; ^6^ Department of Embryology, Faculty of Biology Saint‐Petersburg State University University Embankment, 7/9 Saint‐Petersburg 199034 Russia; ^7^ European Research Institute for the Biology of Ageing, University of Groningen, University Medical Center Groningen Antonius Deusinglaan 1 Groningen 9713 AV The Netherlands; ^8^ Institut Méditerranéen de Biodiversité et d'Ecologie marine et continentale (IMBE), Aix Marseille University, CNRS, IRD, Avignon University Jardin du Pharo, 58 Boulevard Charles Livon Marseille 13007 France; ^9^ Koltzov Institute of Developmental Biology of Russian Academy of Sciences Ulitsa Vavilova, 26 Moscow 119334 Russia; ^10^ Université de Paris, CNRS, Institut Jacques Monod Paris F‐75006 France; ^11^ Department of Pathology Oslo University Hospital Bygg 19, Gaustad Sykehus, Sognsvannsveien 21 Oslo 0188 Norway; ^12^ Collection of Vertebrate Cell Cultures Institute of Cytology, Russian Academy of Sciences Tikhoretsky Ave. 4 St. Petersburg 194064 Russia; ^13^ Université Côte d'Azur, CNRS, INSERM, Institute for Research on Cancer and Aging, Nice (IRCAN) Nice 06107 France; ^14^ Université Côte d'Azur, Federative Research Institute – Marine Resources (IFR MARRES) 28 Avenue de Valrose Nice 06103 France; ^15^ Department of Biology and Evolution of Marine Organisms Stazione Zoologica Anton Dohrn Villa Comunale Naples 80121 Italy; ^16^ Department of Environmental Science and Policy (ESP) Università degli Studi di Milano Via Celoria 26 Milan 20133 Italy; ^17^ Sorbonne Université, CNRS, Laboratoire de Biologie du Développement de Villefranche‐sur‐mer (LBDV) 06234 Villefranche‐sur‐Mer Villefranche sur Mer Cedex France; ^18^ Institute of Zoology and Center for Molecular Biosciences, University of Innsbruck Technikerstr Innsbruck 256020 Austria

**Keywords:** adult stem cells, marine invertebrates, niche, gene expression, Waddington landscape, germ cells, totipotency, cell lineages, regeneration, asexual reproduction

## Abstract

Adult stem cells (ASCs) in vertebrates and model invertebrates (e.g. *Drosophila melanogaster*) are typically long‐lived, lineage‐restricted, clonogenic and quiescent cells with somatic descendants and tissue/organ‐restricted activities. Such ASCs are mostly rare, morphologically undifferentiated, and undergo asymmetric cell division. Characterized by ‘stemness’ gene expression, they can regulate tissue/organ homeostasis, repair and regeneration. By contrast, analysis of other animal phyla shows that ASCs emerge at different life stages, present both differentiated and undifferentiated phenotypes, and may possess amoeboid movement. Usually pluri/totipotent, they may express germ‐cell markers, but often lack germ‐line sequestering, and typically do not reside in discrete niches. ASCs may constitute up to 40% of animal cells, and participate in a range of biological phenomena, from whole‐body regeneration, dormancy, and agametic asexual reproduction, to indeterminate growth. They are considered legitimate units of selection. Conceptualizing this divergence, we present an alternative stemness metaphor to the Waddington landscape: the ‘wobbling Penrose’ landscape. Here, totipotent ASCs adopt ascending/descending courses of an ‘Escherian stairwell’, in a lifelong totipotency pathway. ASCs may also travel along lower stemness echelons to reach fully differentiated states. However, from any starting state, cells can change their stemness status, underscoring their dynamic cellular potencies. Thus, vertebrate ASCs may reflect just one metazoan ASC archetype.

## I. INTRODUCTION

The prevailing vertebrate‐centric paradigm suggests the existence of idiosyncratic populations of adult stem cells (ASCs) in animals (Raff, 2003; Wagers & Weissman, 2004; Clevers, 2015; Wiggans & Pearson, 2021). In vertebrates, ASCs are defined as lineage‐restricted with tissue or organ‐specific activities, and are capable of regulating homeostasis, repair and regeneration of tissues and organs (Clevers & Watt, 2018). Vertebrate ASCs are located in defined niches, where they normally lie in a quiescent state (Slack, 2018; Marescal & Cheeseman, 2020) until called upon to activate by specific stimuli such as injury or disease (Clevers, 2015; Clevers & Watt, 2018). The literature on mammalian stem cells further defines ASCs as undifferentiated cellular entities that give rise to either daughter stem cells, self‐renewing progenitors, or lineage‐specific differentiated cells (Raff, 2003; Clevers & Watt, 2018). While at early embryogenesis vertebrate stem cells are totipotent, giving rise to both somatic and germline descendants, post‐embryonic stem cells are multipotent at best [e.g. haematopoietic stem cells (Raff, 2003; Wagers & Weissman, 2004)].

Over time, two distinct evolving views of ASCs in vertebrates have been proposed. The first considers ASCs as ‘entities’: discrete units of selection, development and regeneration (Weissman, 2000). The second focuses on their ‘state’ or ‘function’, and posits that the biological state of a cell dictates its status as an ASC or as a differentiated cell (Blau & Baltimore, 1991; Blau, Brazelton & Weismann, 2001). The latter view is supported by the controversial findings that restrictions in cell fates are flexible and that differentiated cells may regain levels of lost stemness.

In vertebrates, ASCs have been categorized by their morphology, tissue of origin, plasticity, and potency. While existing in a quiescent state, they still maintain the power to resume cellular proliferation. They tend to be found in small numbers, but are long‐lived as a population, and often express specific ‘stemness’ genes (Poulsom *et al*., 2002; Raff, 2003; Wagers & Weissman, 2004; Clevers, 2015; Rumman, Dhawan & Kassem, 2015; Grün *et al*., 2016; Clevers & Watt, 2018; Marescal & Cheeseman, 2020). Yet other authors have referred to specific ‘conditions’, rather than ‘characters’ or ‘functional potency’ when defining the ASC concept (Loeffler & Roeder, 2002; Zipori, 2004). The above views consider, as a prime defining feature, an ASC's ability to give rise to one or more differentiated cell types as part of regular bodily homeostasis, and in acute states such as those that require repairing damage (Slack, 2018).

**Fig 1:**
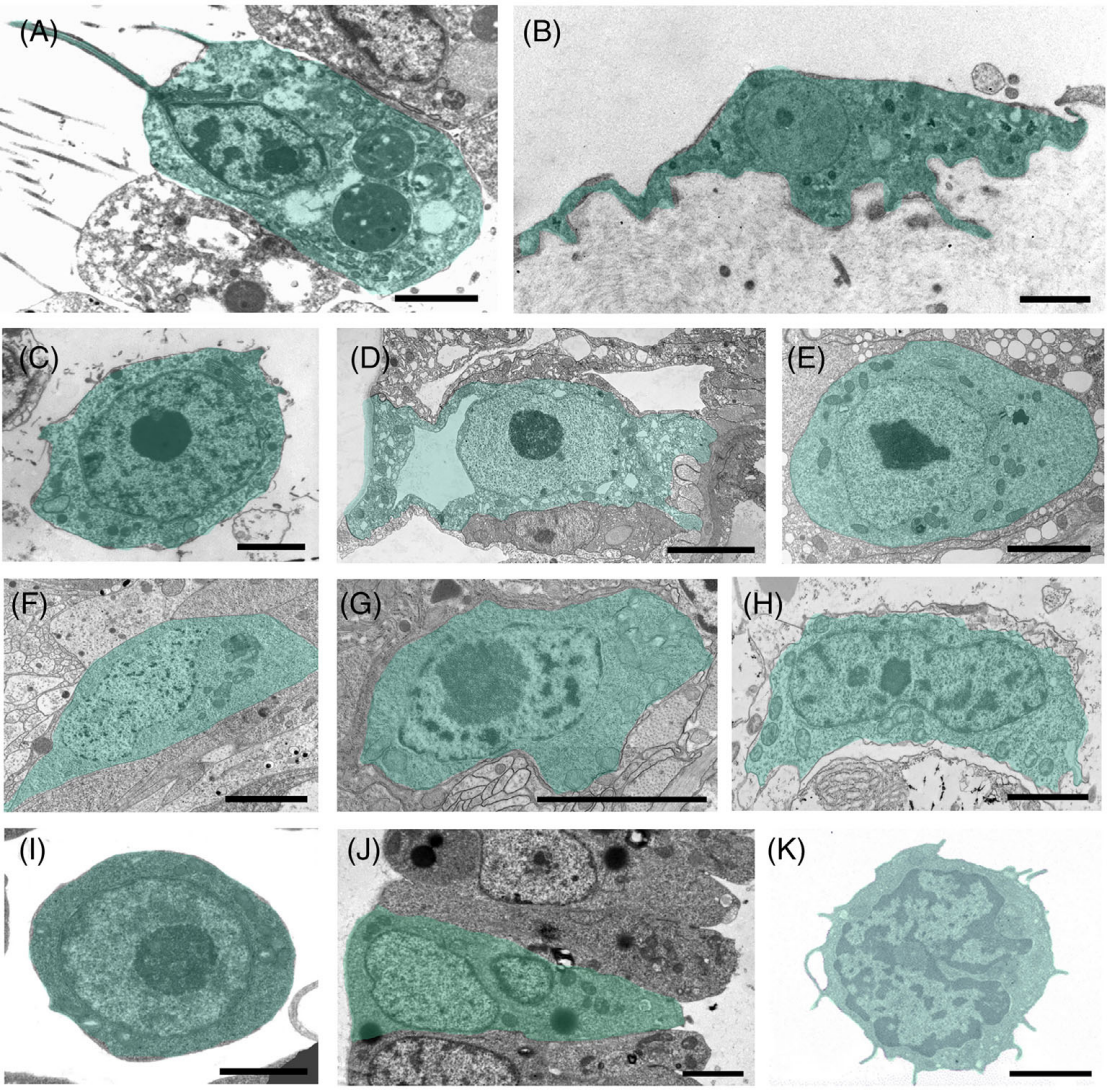
Adult stem cells (ASCs) from selected marine invertebrate phyla and a human haematopoietic stem cell visualized by transmission electron microscopy. (A–C) Porifera: a choanocyte of *Leucosolena variabilis* (A), a pinacocyte of *Oscarella* sp. (B), an archaeocyte of *Crellomima imparidens* (C). (D, E) Cnidaria: epitheliomuscular (D) and interstitial (E) stem cells of *Hydra magnipapillata*. (F, G) Platyhelminthes: neoblasts of the planarian *Schmidtea* sp. (F) and the rhabditophoran *Macrostomum lignano* (G). (H) Acoela: neoblast of *Isodiametra pulchra*. (I, J) Tunicata: haemoblast (I) and bud primordium cell (J) of *Botryllus schlosseri*. (K) Mammalia: quiescent haematopoietic stem cell of *Mus musculus* (modified from Radley *et al*., 1999). ASCs in invertebrates occur as two basic cell types, either as epithelial cells integrated into organized two‐dimensional tissue layers (A, B, D, J) or as smaller cells located in mesenchymal tissues (C, F–H), in interstitial spaces of epithelia (E), and in the circulating haemolymph/blood (I, K). Epithelial ASCs exhibit the hallmarks of typical epithelial cells including a distinct apical–basal polarity. Mammalian ASCs typically show a high nuclear to cytoplasmic ratio, round interphase nuclei with prominent nucleoli, and a ribosome‐rich cytoplasm. Scale bars: A–C, E–K, 5 μm; D, 5 μm. Photograph credits: A–C, A. Ereskovsky; D–H, B. Hobmayer; I, J, L. Manni).

While ASCs are inherently defined morphologically (Fig. 1), phenotype alone provides only tantalizing hints for their identification. For instance, it took decades of targeted research to define the population of haematopoietic stem cells (Eaves, 2015), and many years of work before the discovery of intestinal stem cells (van der Flier & Clevers, 2009). Likewise, other ASC identification criteria may conceal the authentic plasticity in their transcriptome profiles (Grün *et al*., 2016), and the detection of asymmetric cell divisions, often used to identify stem cells ‘unambiguously’, is particularly elusive. Similarly, the criteria of ASC potency and plasticity are a source of confusion (Poulsom *et al*., 2002; Raff, 2003; Wagers & Weissman, 2004). Notwithstanding such caveats, it is widely accepted that vertebrate ASCs are rare, clonogenic, and undifferentiated (characterized by a high nucleo‐cytoplasmic ratio and small cell size compared to lineage‐differentiated progenies). Moreover, they are multi/oligo/unipotent cells capable of self‐renewal and multilineage differentiation, often interacting with specialized stem cell niches, and are considered slow‐cycling cells that show distinct germ/somatic lineage potential. The somatic ASCs are tissue specific and function in homeostasis and, with constraints, in regeneration of organs/tissues.

Does the above vertebrate ASC ‘archetype’ apply to the animal kingdom (Metazoa) as a whole? Comparative approaches may shed light on this important question. A glance at the metazoan phylogenetic tree puts in stark relief the fact that ASCs have only been studied in a limited number of taxa, mainly those capable of asexual reproduction and/or with high competency for regeneration, including some spiralian protostomes (lophotrochozoans, i.e. Platyhelminthes) and deuterostomes (i.e. tunicates, echinoderms), as well as many non‐bilaterian lineages (i.e. cnidarians, poriferans). Somatic ASCs have not been reported in most ecdysozoans (i.e. Nematoidea, Scalidophora and Panarthropoda), except for a few arthropods (Shukalyuk *et al*., 2007; Alié *et al*., 2015).

To fill this conceptual lacuna, we evaluate the distribution and the properties of ASCs in non‐vertebrate metazoans in the context of the vertebrate ASC exemplar, excluding invertebrates such as fruit flies and nematodes, which while excellent genetic model systems are by all accounts highly derived ecdysozoans. Using inter/intra‐phyla comparative analyses of ASC properties, their gene expression and the cellular environment, as well as their role in unique biological processes (e.g. whole‐body regeneration), we put forward the hypothesis that vertebrates represent only one particular prototype of ASC, and that ASCs in fact exhibit a wider range of properties and abilities, some non‐existent in vertebrates. In light of this, we propose a unified model to explain ASC diversity in metazoans – ‘the wobbling Penrose landscape’, a modification of the traditional Waddington landscape metaphor.

## II. VERTEBRATE *VERSUS* INVERTEBRATE ASCs AT A GLANCE

Apart from two fundamental properties of stem cells, i.e. self‐renewal and differentiation potential, it appears that many cardinal ASC traits differ between vertebrates and other phyla. Fifteen traits are highlighted in Table 1, together spanning a wide range of characteristics from morphology, differentiation state and somatic/germ lineage characteristics, to some key biological properties. Vertebrate ASCs are constrained to one of the three germ layers (Weissman, Anderson & Gage, 2001) and they give rise to lineage‐restricted progenies that are limited to specific organs/tissues (Tanaka & Reddien, 2011), with the germline being sequestered from the somatic lineages early in ontogeny. ASCs are generally rare in vertebrates (e.g. only 0.001–0.01% of mononuclear cells isolated from a Ficoll density gradient of feline bone marrow aspirate are mesenchymal stem cells; Martin *et al*., 2002) and pluripotent at best; they are slow cycling and reside in compartmentalized niches, with restricted migration potential (Moore & Lyle, 2011). These vertebrate traits are inconsistent with many of the ASC attributes found in other groups (Table 1). Even the statement that the ‘ability of stem cells to reside within niches is an evolutionarily conserved phenomenon’ (Fuchs, Tumbar & Guasch, 2004, p. 771) is not applicable to all, or even the majority, of metazoan ASCs. Further, ASCs in other lineages may arise *de novo* by trans‐differentiation from somatic cells (Ferrario *et al*., 2020), which is not a common phenomenon in the vertebrates (Goodell, Nguyen & Shroyer, 2015; Merrell & Stanger, 2016), and even from germ cells under specific conditions (Table 1). The aforementioned disparate characters have particularly emerged in long‐lived and indeterminately growing animals, where organismal senescence (sensu Rinkevich & Loya, 1986) has not been documented or is delayed (e.g. sponges, corals, and the immortal *Hydra*).

**Table 1:** Central traits distinguishing vertebrate adult stem cell (ASCs) from non‐ecdysozoan invertebrate ASCs [selected citations; aberrant status such as cancer cells in vertebrates or reprogramming approaches such as iPS (induced pluripotent stem) cells are not included]

ASC trait	Status in vertebrates	Status in marine invertebrates (most cases)
Abundance	Rare, 0.001–0.01% (e.g. Martin *et al*., 2002)	Up to 20–30% of all cells in flatworms (Handberg‐Thorsager, Fernandez & Salo, 2008; Gentile, Cebrià & Bartscherer, 2011); 20–30% of all cells in the freshwater hydrozoan *Hydra* (Bosch *et al*., 2010; Hobmayer *et al*., 2012); up to 50–80% (choanocytes) of all cells in Calcarea (Jones, 1961; A. E., unpublished results) and up to 3–14% of all cells in Demospongiae (Diaz, 1979; Custodio, Hajdu & Muricy, 2004).
Potency	Primarily uni/oligopotency, some pluripotency	Pluri‐ and totipotency, with differentiation potential towards cell lineages from more than a single germ layer (Müller, Teo & Frank, 2004; Manni *et al*., 2007; Rinkevich & Matranga, 2009; Rinkevich, Matranga & Rinkevich, 2009; Wagner, Wang & Reddien, 2011; Reyes‐Bermudez, Hidaka & Mikheyev, 2021).
Stemness outcomes	Limited to organs and tissues	May develop whole organisms *via* asexual reproduction (e.g. budding) or *via* regeneration of minute fragments (Manni *et al*., 2007, 2019; Rinkevich *et al*., 2007, 2009, 2011; Voskoboynik *et al*., 2007; Bely & Nyberg, 2010; Bosch *et al*., 2010; Lehoczky, Robert & Tabin, 2011; Lavrov & Kosevich, 2016; Lai & Aboobaker, 2018).
Amoeboid cell motility	Not recorded under normal conditions	Demosponge archaeocytes (Funayama, 2008), hydrozoan interstitial cells (Bode, 1996), planarian neoblasts (Isaeva, Aleksandrova & Reunov, 2005*a*; Abnave *et al*., 2017) and amoebocytes in stellate echinoderms (Khadra *et al*., 2018) competent for amoeboid motility and/or active migration.
Exhibiting morphologies of differentiated cells	Not recorded under normal conditions	Recorded in various phyla. Examples are the morphologies of choanocytes in sponges and amoebocytes in anthozoans (Gold & Jacobs, 2013; Ereskovsky *et al*., 2015; Funayama, 2018); epithelial cells in *Hydra* (Hobmayer *et al*., 2012); filopodia/extended cell processes in flatworm neoblasts (Baguñà, 2012; Abnave *et al*., 2017; Ivankovic *et al*., 2019); also hypothesized for flagellated coelomic epithelial cells in a starfish (Bossche & Jangoux, 1976).
Soma/germ stem cell boundaries	The germline is sequestered at early ontogeny	Boundaries between soma/germ stem cells are blurred in many taxa and germ cells can arise from ASCs (Buss, 1982; Blackstone & Jasker, 2003; Rinkevich & Yankelevich, 2004; Seipel, Yanze & Schmid, 2004; Rinkevich *et al*., 2009; Rosner *et al*., 2009; Gold & Jacobs, 2013; Dannenberg & Seaver, 2018; DuBuc *et al*., 2020; Vasquez‐Kuntz *et al*., 2020).
Expression of germ cell markers in ASCs	Not recorded (except in some cancers)	Present in ASCs and various somatic cells (e.g. *Vasa*, *Piwi* and *POU* genes) (Raz, 2000; Mochizuki, Nishimiya‐Fujisawa & Fujisawa, 2001; Seipel *et al*., 2004; Shukalyuk *et al*., 2007; Rosner *et al*., 2009; Rinkevich *et al*., 2010; Rosner & Rinkevich, 2011; Fierro‐Constaín *et al*., 2017; Xu & Sun, 2020).
Germ stem cell trans‐differentiation to ASCs	Not recorded	Present, recorded in some regenerative scenarios such as in flatworms (Gremigni & Puccinelli, 1977).
*De novo* emergence of ASCs	Not recorded	Present in cnidarians, sponges and tunicates (Müller *et al*., 2004; Manni *et al*., 2007, 2019; Schmich *et al*., 2007; Rinkevich *et al*., 2010; Rinkevich & Rinkevich, 2013; Borisenko *et al*., 2015; Ereskovsky *et al*., 2015; Ferrario *et al*., 2020; Xu & Sun, 2020).
Source cells for regeneration	Tissue resident; mostly lineage‐restricted ASCs	Whole organismal residency; potential mobilization and expansion of ASCs from other sites/tissues; in planarians and some tunicates, a single ASC may regenerate a whole organism (Rinkevich, Shlemberg & Fishelson, 1995; Rinkevich *et al*., 2010, 2011; Lehoczky *et al*., 2011; Wagner *et al*., 2011; Rinkevich & Rinkevich, 2013; Blanchoud, Rinkevich & Wilson, 2018; Fields & Levin, 2018). Presence of dedifferentiation processes (Ferrario *et al*., 2020; Xu & Sun, 2020).
Contribution to dormancy	Inconclusive	Hibernation and aestivation in botryllid ascidians (Hyams *et al*., 2017).
ASC niche	Essential for ASC quiescence and long‐term survival (Marescal & Cheeseman, 2020)	No distinct anatomical stem cell niche has been elucidated for the vast majority of non‐ecdysozoan invertebrates (Rinkevich, 2009; Rinkevich *et al*., 2009). A few ephemeral ASC niches were identified in botryllid ascidians (Voskoboynik *et al*., 2008; Rinkevich *et al*., 2013; Rosner *et al*., 2013; Rosental *et al*., 2018).
Contribution to indeterminate growth	Indeterminate growth does not exist in birds and mammals	Indeterminate growth exists in various taxa within sponges, cnidarians, annelids, bryozoans, and tunicates (Jackson & Coates, 1986; Hughes, 1987; Vogt, 2012; Gazave *et al*., 2013); Direct evidence for the role of ASCs found in sponges, flatworms, cnidarians and annelids (e.g. in atokous worms).
Contribution to immortal lifespan	Immortality does not exist	Immortality exists in cnidarians (Martínez, 1998; Schmich *et al*., 2007; Dańko, Kozłowski & Schaible, 2015), planarians [further associated with neoblasts (Saló, 2006; Tan *et al*., 2012)] and sponges (which may live for thousands of years) (Gatti, 2002; McMurray, Blum & Pawlik, 2008); extended lifespan in bivalves, the longest lived non‐colonial animals (Gruber *et al*., 2015).
ASCs as units of selection	Unspecified; yes, in transmissible tumours	Present, potentially in all marine invertebrates with a somatic embryogenesis type of ontogeny (Buss, 1982; Rinkevich, 2000, 2009, 2011; Weissman, 2000; Laird, De Tomaso & Weissman, 2005; Fields & Levin, 2018).

## III. THE WIDE RANGE OF METAZOAN ASC MORPHOTYPES

**Fig 2:**
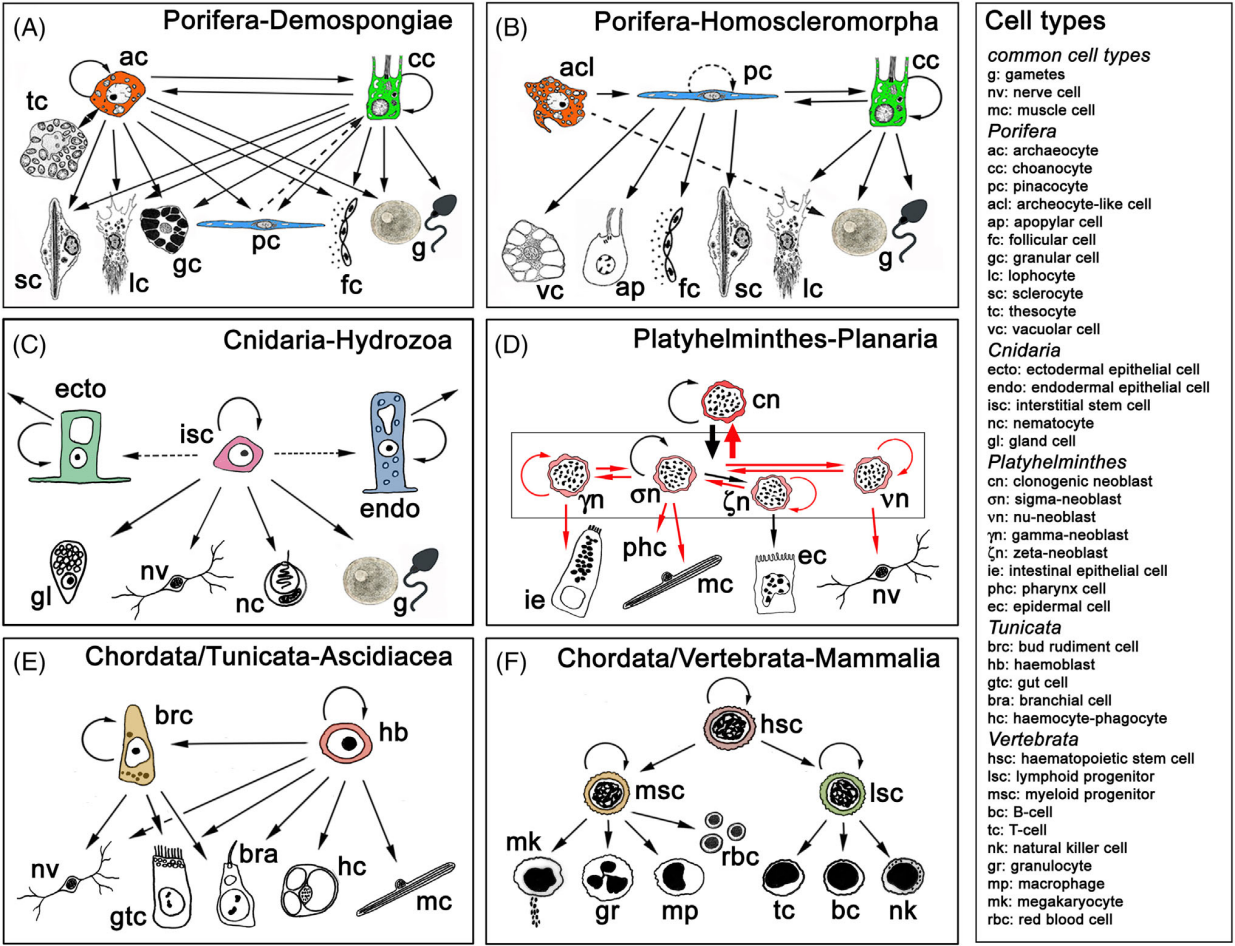
Plasticity, self‐renewal, and differentiation dynamics in selected invertebrate and vertebrate adult stem cell (ASC) lineages. ASCs are highlighted in colour, differentiation products shown as black and white schemes. Conversion of one ASC type into another occurs in pre‐bilaterian sponges and hydrozoans, and within the flatworm neoblast lineage (A–D). Differentiation of gametes as descendants of ASCs is a common feature in the pre‐bilaterian sponges and hydrozoans (A, B). The dashed arrows in sponge ASC lineages represent capacities for self‐renewal, phenotypic conversion and differentiation based on observations of cellular behaviour during growth, tissue renewal and regeneration, which have not yet been validated by stringent experimental analysis. In C stippled arrows represent the formation of hydrozoan epithelial cells from interstitial stem cells as described in *Hydractinia* spp., which does not occur in *Hydra* spp. In D red arrows in the planarian neoblast system are based on the lineage‐restricted expression of gene sets, which require further validation using precise lineage tracing and functional interference assays. The self‐renewal capacity of zeta‐, gamma‐, and nu‐neoblasts is under discussion. Species sources: (A) *Amphimedon queenslandica*, *Ephydatia fluviatilis*; (B) *Oscarella lobularis*; (C) *Hydractinia* spp., *Hydra vulgaris*; (D) *Schmidtea mediterranea*; (E) *Botryllus schlosseri*, *Ciona robusta*; (F) *Homo sapiens*. Schemes in C and D are modified from Gold & Jacobs (2013) and Zhu & Pearson (2016), respectively.

Almost no study on ASCs outside vertebrates has been devoted to capturing their degree of potency by using criteria of increased stringency, as has recently been proposed for mammalian systems (Posfai *et al*., 2021). However, many phyla (e.g. Porifera, Cnidaria, Ctenophora, Annelida, Acoela, Platyhelminthes, Echinodermata, Cephalochordata and Tunicata) possess large pools of *bona fide* ASCs throughout the lifespan of the organism, most of which are multipotent (in sponges, flatworms, acoels, cnidarians, annelids and tunicates; Fig. 2; see online Supporting Information, Tables S1 and S2) and some of which (e.g. cnidarians, flatworms and tunicates) have been suggested to be totipotent (Müller *et al*., 2004; Manni *et al*., 2007; Rinkevich & Matranga, 2009; Rinkevich, Matranga & Rinkevich, 2009; Wagner *et al*., 2011; Kassmer, Langenbacher & De Tomaso, 2020). In many groups, ASCs give rise not only to somatic lineages, but also to germ cell lineages, with no signature of germ‐cell sequestration (Gschwentner *et al*., 2001; Takamura, Fujimura & Yamaguchi, 2002; Rinkevich, 2009; Juliano, Swartz & Wessel, 2010; Juliano & Wessel, 2010; Gold & Jacobs, 2013; Solana, 2013; Yoshida *et al*., 2017; Adamska, 2018; Dannenberg & Seaver, 2018; DuBuc *et al*., 2020; Vasquez‐Kuntz *et al*., 2020), and in some animals, ASCs are the only proliferative cells (Bely & Sikes, 2010*a*).

ASCs in invertebrates represent a wide range of phylum‐specific and characteristic cell types, morphologies and behaviours (Figs 1 and 2; Table S1), which range from sponge archaeocytes and choanocytes (Simpson, 1984; Ereskovsky, 2010), hydrozoan interstitial cells (i‐cells) (Bosch, 2009; Plickert, Frank & Müller, 2012) and platyhelminth or acoel neoblasts (Wagner *et al*., 2011; Baguñà, 2012) to tunicate haemoblasts (Freeman, 1964; Voskoboynik *et al*., 2008; Kawamura & Sunanaga, 2010; Rinkevich *et al*., 2013; Kassmer *et al*., 2020). Comparisons within phyla reveal a considerable degree of additional variation, where ASC properties are possessed only by particular taxa within a phylum (e.g. demosponge archaeocytes, hydrozoan i‐cells). Similarly, ASC lineages and progenitors may show intra‐phylum modifications (e.g. Müller *et al*., 2004; Borisenko *et al*., 2015; Funayama, 2018; Lavrov *et al*., 2018; Fig. 2; Table S1).

Outside the vertebrates, ASCs are often highly abundant (primarily choanocytes in sponges, ecto/endodermal epitheliomuscular cells in cnidarian polyps and neoblasts in flatworms; Simpson, 1984; Handberg‐Thorsager *et al*., 2008; Bosch *et al*., 2010; Gentile *et al*., 2011; Hobmayer *et al*., 2012; Table 1) and the literature reveals cases of putative totipotency, as high differentiation potential contributes to more than a single germ layer (Fig. 2; Rinkevich *et al*., 2009; Wagner *et al*., 2011). Emblematic structures in many of these ASCs are the so‐called chromatoid bodies [reported in neoblasts, i‐cells and archaeocytes, as well as most recently in a small pool of notochord cells in cephalochordates (Rossi *et al*., 2008; Isaeva *et al*., 2009; Isaeva & Akhmadiev, 2011; Holland & Somorjai, 2020)] – electron‐dense aggregates often adjacent to the nuclear envelope that resemble the germline granules of vertebrates and insects.

Many invertebrate ASCs consist of epithelial tissues, exhibiting epithelial cell hallmarks (lacking the characteristic large nucleus/cytoplasmic ratio of other ASCs), with distinct apical–basal and planar cell polarities, apical cell–cell junctions, and basal cell–extracellular matrix interactions, all of which are features of differentiated cells. The most prominent example is found in sponges, where cells of the inner and outer epithelia – choanocytes and pinacocytes, respectively (Fig. 1A, B) – function as true epithelial cells, while potentially acting as stem cells during tissue renewal and regeneration (Ereskovsky *et al*., 2015; Lavrov *et al*., 2018). The same applies to the hydrozoan cnidarian ectodermal and endodermal epitheliomuscular cells (Fig. 1D; Bosch *et al*., 2010; Hobmayer *et al*., 2012) and the colonial tunicate bud primordium (Fig. 1J; Manni *et al*., 2007, 2019). Further, the literature reveals cases where these ASCs not only express genes associated with germline stem cells, but are also able to differentiate into somatic cells or gametes, indicating the lack of strict boundaries between somatic/germline lineages. Examples include sponge archaeocytes and choanocytes (Fierro‐Constaín *et al*., 2017; Funayama, 2018), hydrozoan i‐cells (Bode, 1996), flatworm and acoel neoblasts (Shibata, Rouhana & Agata, 2010; Chiodin *et al*., 2013; Lai & Aboobaker, 2018), the posterior stem cells of the annelid growth zone (Giani *et al*., 2011; Gazave *et al*., 2013; Kozin & Kostyuchenko, 2015) and tunicate haemoblasts (Magor *et al*., 1999; Stoner, Rinkevich & Weissman, 1999; Laird *et al*., 2005; Voskoboynik *et al*., 2007; Rosner *et al*., 2009; Brown *et al*., 2009*a*; Rinkevich, 2017; Rosner, Kravchenko & Rinkevich, 2019; Kassmer *et al*., 2020).

## IV. GENE EXPRESSION IN INVERTEBRATE ASCs

**Fig 3:**
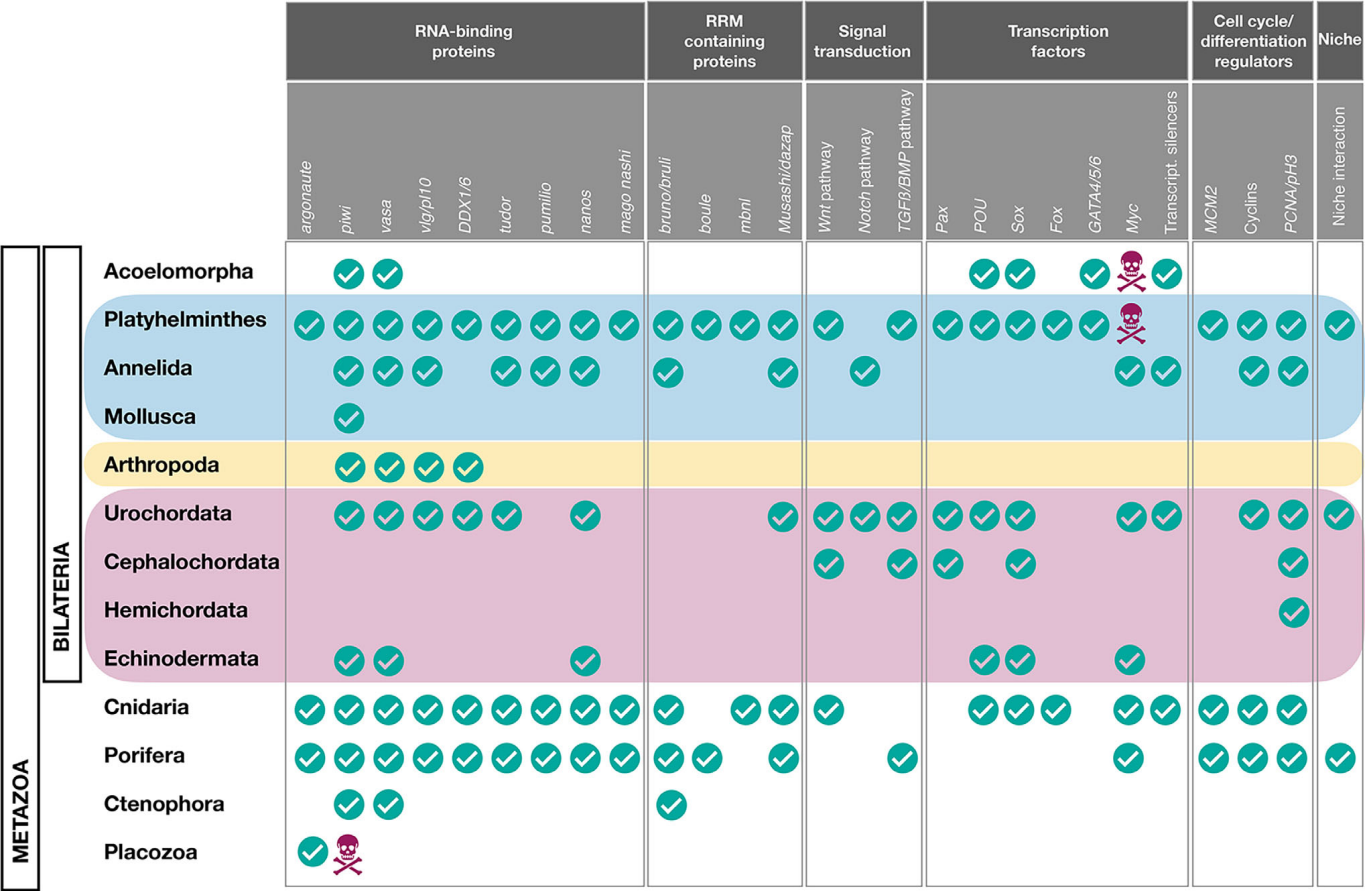
The expression of ‘stemness’ genes in somatic cells of invertebrates. Five functional gene categories are depicted, each represented by 3–9 specific genes (in grey boxes). Bilaterian phyla are grouped by colour, with pink for Deuterostomia (Chordata and Ambulacraria) and blue (Spiralia) and yellow (Ecsysozoa) for Protostomia. Ticks indicate that expression of stemness genes in ASCs in at least one species for the phylum has been reported. Note that for most metazoan phyla and many gene categories, no data are available. Only taxa for which sufficient information on ASCs is available are included. The red skull and crossbones indicate the absence/loss of the gene(s) in the phylum. RRM, RNA‐recognition motif. Data from model ecdysozoans are excluded (*Drosophila*, nematodes; see text for details). See Table S2 for the original data on which this figure is based.

Invertebrate ASCs express orthologues of many vertebrate ‘stemness’ genes, as well as genes that contribute to cancer cell ‘stem cell potential’ (Conte *et al*., 2009; Mashanov *et al*., 2010; Yun *et al*., 2017; Ben‐Hamo *et al*., 2018). A list of selected genes and gene families is provided in Fig. 3 and Table S2. However, it is challenging to identify or compare stemness gene signatures across diverse taxa separated by wide evolutionary distances (Alié *et al*., 2015; Wiggans & Pearson, 2021). Also, the molecular mechanisms by which invertebrates maintain viable ASC stocks, with long‐term stability and constant proliferation during their lifespan, remain elusive (Conte *et al*., 2009). This is true for *Myc*, one of the major vertebrate stem cell maintenance factors, and which has been associated with ASC self‐renewal in hydrozoan i‐cells (Hartl *et al*., 2010, 2019; Plickert *et al*., 2012). In‐depth single‐cell transcriptome analysis of hydrozoan i‐cell and flatworm neoblast lineages failed to identify common sets of stemness factors (Fincher *et al*., 2018; Plass *et al*., 2018; Siebert *et al*., 2019).

As in the vertebrates (Lander, 2009), the essence of ASC stemness cannot be distilled down to a single shared molecular fingerprint, further highlighted by the co‐expression of somatic/germ stem cell signatures in invertebrate ASCs (Table 1), wherever these ASCs have been studied. This includes the expression of genes such as *POU*, *SOX*, *Piwi*, *Bruno*, *Vasa* and *Pl10* orthologues in a number of metazoan ASCs, including sponge archaeocytes and choanocytes (Funayama, 2008, 2018; Fierro‐Constaín *et al*., 2017), hydrozoan i‐cells (Seipel *et al*., 2004; Rebscher *et al*., 2008; Leclère *et al*., 2012), neoblasts of acoels and planarians (Guo, Peters & Newmark, 2006; Pfister *et al*., 2008; De Mulder *et al*., 2009*b*; Önal *et al*., 2012), tunicate ASCs (Sunanaga, Watanabe & Kawamura, 2007; Rosner *et al*., 2009, 2019; Rinkevich *et al*., 2010), putative stem cells from annelid growth zones (Rebscher *et al*., 2007; Giani *et al*., 2011; Gazave *et al*., 2013), and presumably in regenerating nemertean tissues (Xu & Sun, 2020). This toti/pluripotency in non‐vertebrate phyla maintains functions such as gametogenesis, embryogenesis, homeostasis, asexual reproduction and regeneration (Fierro‐Constaín *et al*., 2017), supporting the idea of global conservation in pluripotency‐associated genes for day‐to‐day needs (Fig. 3; Table S2), as reported for cell adhesion receptors and nuclear receptors (Gamulin *et al*., 1994).

In contrast to the vertebrates, somatic and germline stemness markers (e.g. *Vasa*, *Pl10*, *Piwi*, *Nanos*, *Bruno*, *Pumilio*, *Tudor*, etc.; Fig. 3), as well as alkaline phosphatase (Isaeva, 2011), are co‐expressed in differentiated somatic cells/tissues in many invertebrate phyla (Table 1). This character has been recorded in sponges (Funayama, 2018), cnidarians (Mochizuki *et al*., 2001), ctenophores (Alié *et al*., 2011), annelids (Rebscher *et al*., 2007; Dill & Seaver, 2008; Gazave *et al*., 2013), parasitic crustaceans (Shukalyuk *et al*., 2007; Shukayuk & Isaeva, 2012), molluscs and echinoderms (Lai & Aboobaker, 2018) and colonial tunicates (Rabinowitz, Alphasi & Rinkevich, 2009; Rosner *et al*., 2009; Brown *et al*., 2009*a*; Rinkevich *et al*., 2010; Rabinowitz & Rinkevich, 2011). These observations may either imply distinct functions or pleiotropy for the genes in differentiated somatic cells (Juliano *et al*., 2010). Alternatively, this may suggest that the conventional view of distinct ‘stem cell genes’ should be reconsidered.

By tracing shared transcriptomic signatures for demosponge archaeocytes, flatworm neoblasts and *Hydra* i‐cells, Alié *et al*. (2015) revealed 180 orthology groups, considered as a relevant proxy for the core set for ancestral stem cells. Most of these genes pre‐dated animal origins, with only a few representing true metazoan innovations. These findings reinforce the idea of a conserved ancestral multipotency program associated with pluri/totipotency (Önal *et al*., 2012; Fierro‐Constaín *et al*., 2017; Fig. 3), although the putative gene regulatory networks have been rewired throughout evolution to generate clade‐specific morphologies/physiologies. These observations are in line with the hypothesis of the existence of primordial stem cells (Solana, 2013). Interestingly, the ancestral stem cell transcriptomic landscape (Alié *et al*., 2015) is noticeably poor in transcription factors, yet it is rich in RNA regulatory players, including many RNA‐binding proteins, which are typical regulators of mammalian embryonic stem cells.

## V. THE ENVIRONMENT – ASC NICHES IN INVERTEBRATES

The term ‘stem cell niche’, originally conceptualized by Schofield (1978), refers to a discrete anatomical microenvironment within which stem cells reside, as well as their milieu, which together play critical roles in maintaining/regulating ‘stemness’ properties (Spradling, Drummond‐Barbosa & Kai, 2001; Fuchs *et al*., 2004; Li & Xie, 2005; Saez, Yusuf & Scadden, 2017). Morphologically, all ‘niches’ consist of homing stem cells and their progeny, heterologous cell types and the surrounding niche‐specific extracellular matrix (Chacón‐Martínez, Koester & Wickström, 2018; Christodoulou *et al*., 2020). Studies in vertebrate models have elucidated a wide range of core elements associated with stem cell niche environments, encompassing networks of cell–cell and cell–extracellular matrix interactions and soluble signalling factors (autocrine, paracrine, systemic), which act as biochemical cues to determine ASC fates and behaviours (Scadden, 2006; Chacón‐Martínez *et al*., 2018; Singh *et al*., 2019). Thus, the maintenance of a niche is associated with, and based on, active crosstalk between ASCs and their niche components (Saez *et al*., 2017; Durand, Charbord & Jaffredo, 2018). The niche architecture in model organisms (e.g. mice, *Caenorhabditis elegans*, *Drosophila melanogaster*) constitutes one of the basic consensus feature central to the definition of ASCs (Slack, 2018).

Fuchs *et al*. (2004) argued that ASC competence to reside within discrete niches is an evolutionarily conserved feature between *Drosophila* and vertebrates, and that ASC niches are armed with shared properties, such as three‐dimensional spaces, basement membranes, extracellular matrices and paracrine signalling (Spradling *et al*., 2001; Scadden, 2006). ASC niches further generate extrinsic factors, such as BMP (bone morphogenetic protein) and Wnt (wingless‐related integration site) signals, that have emerged as common pathways for controlling stem cell self‐renewal and lineage fate from *Drosophila* to mammals (Li & Xie, 2005). Yet no such distinct anatomical stem cell niche has thus far been convincingly elucidated in non‐ecdysozoan invertebrates (Rinkevich, 2009; Rinkevich *et al*., 2009), and few putative stem cell niches have been identified (Table S3) that satisfy the strict criteria set for the vertebrate/insect ASC niches.

While knowledge gained from mammalian, *D. melanogaster* and *C. elegans* models provides guidelines for defining comparable niches in other metazoans, studies on sponge archaeocytes and choanocytes, hydrozoan i‐cells and platyhelminth and acoel neoblasts have failed to define either discrete anatomical microenvironments where stem cells reside, or a niche‐specific extracellular matrix to which ASCs home. Nevertheless, by employing the niche concept more loosely (Morrison & Spradling, 2008), the existence of ‘permissive’ stem cell niches for i‐cells in *Hydra* (e.g. Khalturin *et al*., 2007; Table S3) and for planarians neoblasts (Pellettieri & Sanchez Alvarado, 2007; Dingwall & King, 2016; Table S3) has been proposed. These claims were later adjusted by viewing the whole animal or tissue as a single functional stem cell niche. In *Hydra*, it was first suggested that the body column of the polyp could be considered a stem cell niche (Bosch *et al*., 2010). In planarians, a ‘global niche’ (macro‐environment) tenet was postulated, implying that the potential niche is ‘extended to the entire planarian body, in which long‐range signals, released by various differentiated tissues, regulate stem cell behaviour in response to environmental variations’ (Rossi & Salvetti, 2019, p. 33).

Botryllid ascidians reveal a different scenario relative to other taxa, with putative ASCs homing to discrete, yet ephemeral, microenvironments (Table S3). The first presumed niche, considered a somatic stem cell niche, was identified in the endostyle area (Voskoboynik *et al*., 2008), to which haemoblasts and proliferating cells migrate. Whole‐blood transcriptomes revealed a shared expression of >300 genes with human neural precursors and haematopoietic bone marrow, suggesting that the endostyle represents the haematopoietic stem cell niche (Rosental *et al*., 2018). Rinkevich *et al*. (2013) revealed the transient presence of ASC niches around zooid endostyles, termed ‘cell islands’. They host cycling putative stem cells that migrate weekly *via* the blood vasculature, from degenerating cell islands to newly formed ones in developing buds, which are also regarded as ‘ephemeral soma’ (Qarri *et al*., 2020). Cells within cell islands express a wide range of markers, including somatic stem cell markers [including PKC (protein kinase C), STAT (signal transducer and activator of transcription)], germ cell markers (*Nanos*, *Vasa*, alkaline phosphatase, *Piwi*) and signalling components of the BMP, FGF (fibroblast growth factor) and Slit/Robo (secreted SLIT glycoproteins and their roundabout receptors) pathways. Trafficking of germ stem cells between other putative transient niches was suggested to occur during the weekly blastogenic cycles in botryllid ascidians (Kawamura, Tachibana & Sunanaga, 2008*b*; Rosner *et al*., 2013).

## VI. IDIOSYNCRATIC FEATURES ASSOCIATED WITH ASCs IN INVERTEBRATES

**Fig 4:**
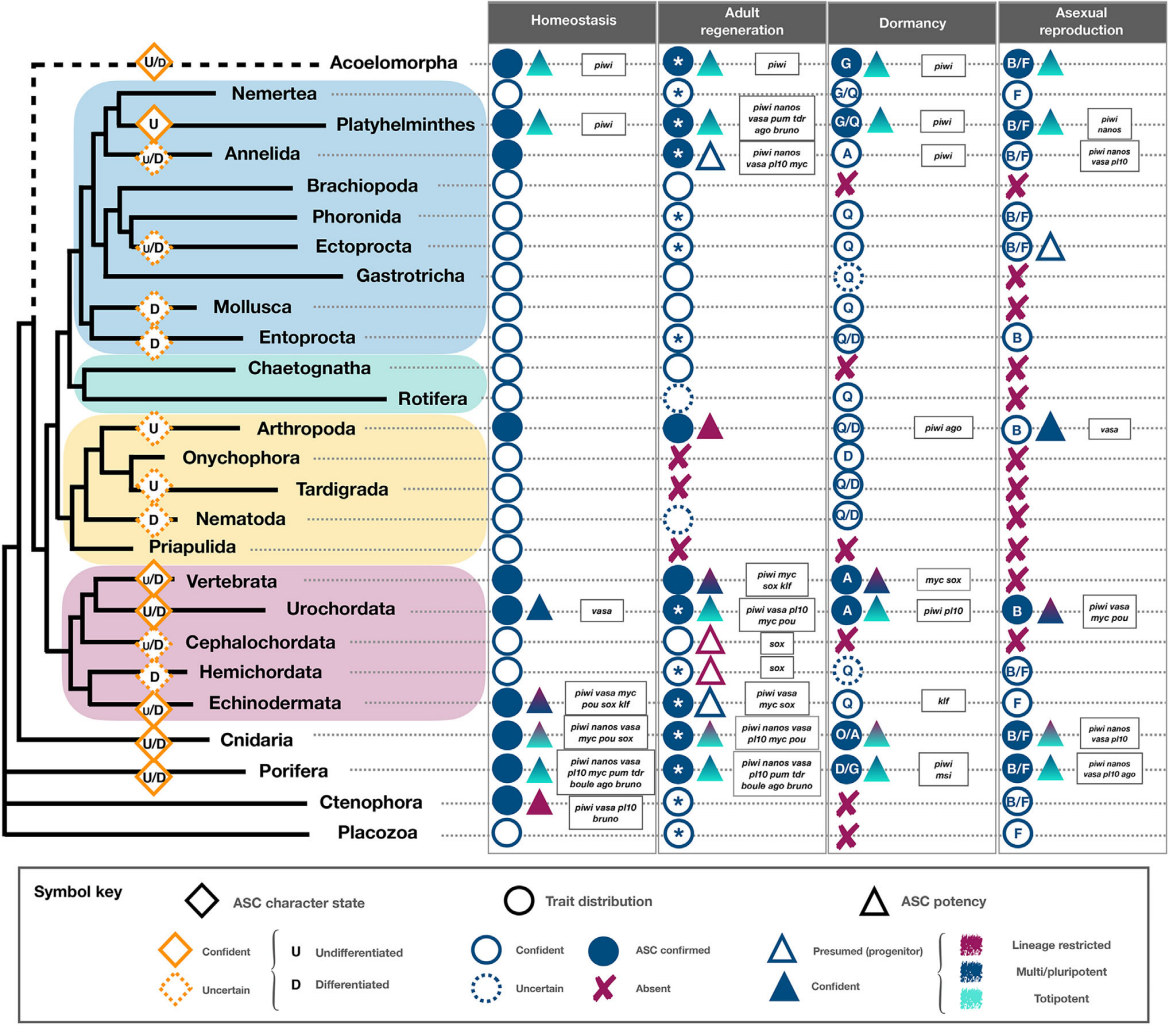
Adult stem cells (ASCs) are involved in four major biological processes in Metazoa: homeostasis, adult regeneration, dormancy and agametic asexual reproduction. The presence of the biological process, involvement of undifferentiated/differentiated putative ASCs or progenitors and their level of potency, as well as the specific classes of stemness gene families they express are mapped for all phyla, when present in at least a single member of the group considered. In the metazoan phylogeny, Deuterostomia are in pink, Ecdysozoa are in yellow, and Spiralia are in green (Gnathifera) and blue (Lophotrochozoa). The position of the Acoelomorpha is debated (dotted line). Circles: empty circle – documented presence of the biological process; filled circle – cases where putative ASCs or progenitors are involved; dotted line circle – inconclusive evidence for the presence of the biological process. A red cross signifies the absence of the biological process in the clade as currently documented. As homeostasis is a property of life, all phyla are shown with an empty circle. For adult regeneration, an asterisk within a circle documents the presence of whole‐body regeneration. Dormancy refers to any documented type of dormant stage or torpor‐like process and has likely evolved independently in each lineage. For dormancy, the dotted line circle indicates potential involvement in non‐adults. A – quiescence, diapause, growth/degrowth; D – diapause; G – growth/degrowth; O – ontogeny reversal; Q – quiescence. For agametic asexual reproduction, B – any form of budding; F – any form of fission/fragmentation. Triangles indicate the level of documented potency for ASCs (filled) and progenitors (empty). Red = lineage restricted/unipotent; cyan = totipotent; blue = multi/pluripotent; gradient triangle = documented cases of several ASCs or progenitors with different potency. Selected stemness gene families whose members are expressed in ASCs or progenitors during the biological process are listed in a box for each process and phylum. The relative contribution of undifferentiated (U) *versus* differentiated (D) ASCs or progenitors within each phylum is mapped onto the phylogeny if known; levels of confidence are represented by solid (higher) and dotted (lower) diamonds, while the sizes of D and U reflect their presumed level of contribution. See Tables [Link], [Link] and Figs. S1 and S2 for the original data used to generate this figure.

Many of the characters used to identify vertebrate ASCs are associated with their functions, primarily with the perpetuation of lineages, replacement of cells due to wear‐and‐tear and the supply of differentiated cells for maintenance (Raff, 2003; Wagers & Weissman, 2004; Morrison & Spradling, 2008; Rumman *et al*., 2015; Clevers & Watt, 2018). By contrast, beyond their functions in supporting homeostasis, ASCs in many metazoans (Fig. 4; Table S4) also play major roles in supporting key biological features such as regeneration in adults, including whole‐body regeneration, and agametic asexual reproduction such as budding and fission (Weissman, 2000; Raff, 2003; Rinkevich *et al*., 2007, 2009, 2011; De Mulder *et al*., 2009*b*; Isaeva *et al*., 2009; Bely & Nyberg, 2010; Funayama, 2018; Lai & Aboobaker, 2018; Ivankovic *et al*., 2019; Rossi & Salvetti, 2019; Tables S5 and S6; Figs S1 and S2), as well as regulation of dormancy or torpor‐like states (Hyams *et al*., 2017; Table S7).

A comprehensive survey across 26 metazoan phyla identifies ASCs and progenitors with putative roles in homeostasis (10 out of 26 phyla), regeneration (9 out of 23 phyla able to regenerate, of which 14 exhibit the capacity for whole‐body‐regeneration), asexual reproduction (5 out of 15 phyla), and in regulating dormant states (6 out of 20 phyla; Fig. 4; Tables [Link], [Link]). Regeneration patterns, type of dormancy and asexual modes of reproduction differ among phyla (Fig. 4) as well as within specific taxonomic groups (Tables [Link], [Link]; Figs S1 and S2), and are further tuned by the contributions of dedifferentiation processes (Ferrario *et al*., 2020). While proper identification of stem cells or lineage‐committed progenitor cells is still lacking for many lineages, the literature already indicates major differences between ASCs in various species in terms of general and specific markers for ASCs (the current terminology is based on the vertebrate ASC literature). Many metazoan phyla show ASC‐associated phenomena not recorded in vertebrates, both under normal physiological and hostile environmental conditions, including whole‐body regeneration, budding, fission and fusion of body fragments, and cycles of growth/decay. When studied in detail, the involvement of multi/pluri/totipotent ASCs is often revealed (Fig. 4; Tables [Link], [Link]; Figs S1 and S2). Thus, at least some ASCs in invertebrates can produce differentiated lineages and can impart stemness at the totipotent level.

An additional biological feature of ASCs is their roles in organisms with indeterminate growth (where growth does not cease at adulthood), reflecting an unfolding ontogenic trait from birth to death (Vogt, 2012). This rarely studied phenomenon is characteristic of particular lineages (e.g. bivalve molluscs, echinoderms, solitary ascidians, annelids) as well as colonial/modular marine invertebrates (e.g. corals, sponges, bryozoans, ascidians).

## VII. DISCUSSION

This review describes ASC states across the breadth of non‐vertebrate metazoans, fuelling the argument that ASCs in many taxa possess modified and diversified repertoires relative to the status and properties of vertebrate ASCs. Indeed, current ACS concepts were constructed from studies on vertebrates and select canonical ecdysozoan models (fruit flies, nematodes). It is evident that the ASC attributes detailed here are not shared by all animal phyla. However, cumulatively this review emphasizes that vertebrate ASCs represent a ‘unique’ case that could be considered distinct from most other animals. Additional work is needed to reach a better understanding of ASC diversity and properties in other lineages in order to obtain a comprehensive view of the similarities and differences across the Metazoa.

ASCs in many aquatic invertebrates are the engine for agametic asexual reproduction and whole‐body regeneration; they can be far from rare (up to 40% of the animal's cells), and encompass entities with unorthodox cellular shapes and behaviours (e.g. amoeboid movement). These ASCs drive whole‐organismal functions (dormancy, fission, fragmentation, budding); co‐express repertoires of germ and somatic lineage markers, refuting the rule of germ cell sequestration; and may emerge *de novo* according to need, without the requirement for a stem cell niche. Additionally, as the shared stemness capacity of all ASCs ‘cannot be reduced to the molecular properties of individual cells’ (Lander, 2009, p. 5), we suggest that other ASCs exhibiting extensive lineage‐specific adaptations or distant evolutionary affinities of ‘stemness’ may go unnoticed.

**Fig 5:**
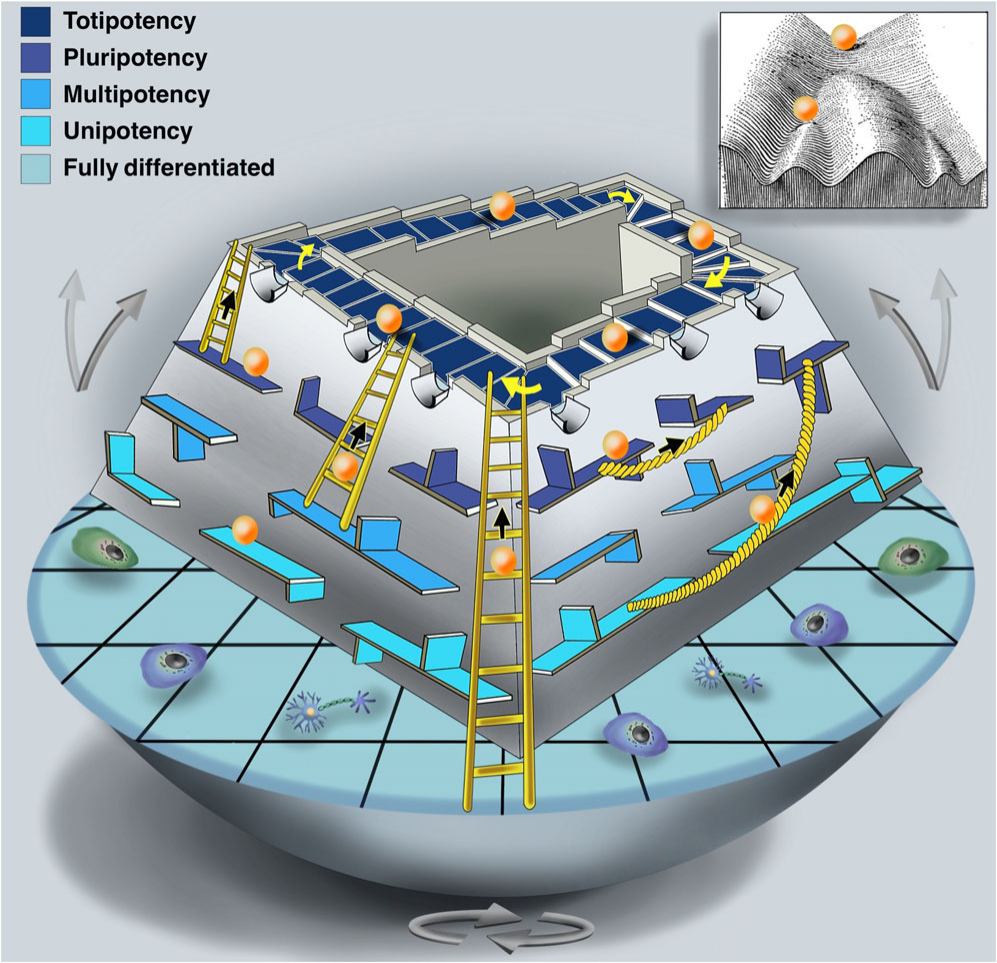
A graphical visualization of the ‘wobbling Penrose landscape’ metaphor. In the Penrose Staircase of stemness (the dark‐blue stairs), totipotent adult stem cells (ASCs) make turns in ascending or descending courses, forming a continuous loop, so that the stemness course of a totipotent stem cell could extend throughout ontogeny (presenting endless totipotency; with no niche involvement) and never acquires any upper or lower values. At any step during this journey (represented by funnels), an ASC may start a labyrinthine journey down stemness echelons (the grey downhill walls), descending from one tier (where they can stay, or continue onwards) to a lower one, downhill to a fully differentiated state (with multipotency to unipotency levels of stemness correspondingly coloured in paler blues, see key). The Penrose landscape carries the property of Escherian movement, allowing continuous passage of stem cells at any stemness status either up (towards totipotency, even from fully differentiated states; shown by the ladders) or sideways to change their stemness status (through transdifferentiation/dedifferentiation; shown by the ropes). In the Penrose landscape, as opposed to the hilly Waddingtonian landscape metaphor (see insert), there is no automatic downhill route (symbolized by valleys) in potency and no determinant bifurcated choices, but stemness is portrayed by a flexible, multi‐choice status without a decisive fate. Depending on the internal and external cues experienced, the Penrose landscape can ‘wobble’, representing a dynamic landscape of stemness. Not all ASCs from every lineage display the full range of movements possible within the wobbling Penrose landscape, but the cumulative data suggest its existence.

The traditional powerful metaphor of Waddington's landscape (e.g. Waddington, 1957; Noble, 2015; Moris, Pina & Arias, 2016; Rajagopal & Stanger, 2016), is an iconic illustration that describes how sequential developmental fate decisions allow an ASC to transform along alternative descending cell lineages. Discussed extensively, this metaphor reveals the conceptual framework for ASC stemness, hitherto through the vertebrate perspective. However, Waddington's metaphor does not cover many ASC phenomena, such as regeneration in non‐vertebrate deuterostomes (echinoderms, hemichordates and cephalochordates), which is largely based on local dedifferentiation rather than on undifferentiated ASCs (Ferrario *et al*., 2020) or transdifferentiation in regenerating medusae (Schmid & Reber‐Muller, 1995). These disparities lead us to propose an alternative metaphor, termed the ‘wobbling Penrose landscape’, which illustrates metazoan stemness better (Fig. 5). It defines the continuously acquired totipotency through ontogeny and astogeny observed in many phyla, and thus differs fundamentally from the unidirectional trajectory of differentiation in the Vertebrata, typified by gradually diminished cellular potency through ontogeny.

In the classical Waddington's landscape metaphor (Waddington, 1957; see inset to Fig. 5), a stem cell begins its journey at the top of a hill (representing the highest stemness level, or totipotency) and slides down to bi‐ or multifurcated paths within inescapable valleys (signifying determined fates) in a landscape driven by a metaphorical gravitational force, which guides the cell into one of several possible decisions or fates (each leads to a different cell type and altered level of specification). The kernel of ASC stemness in invertebrates, on the other hand, relies on the logic of the Penrose staircase (https://en.wikipedia.org/wiki/Penrose_stairs), an ‘Escherian stairwell’ of stemness. Here the stairs make turns in ascending or descending courses, yet form continuous loops, from birth to death, where the totipotent stemness course of a stem cell lasts for the duration of the animal's lifespan (Fig. 5). At any point in the Penrose staircase, an ASC may start a journey down stemness echelons to initiate cascades of cellular phenotypes and lineage segregations that recapitulate hierarchies of potency and differentiated cell types. This cascading landscape further allows cells at any point in the slope to turn back into an ascending trajectory towards higher levels of stem cell potency. Cells may thus travel all the way up to the Penrosian loop of totipotency, or move to different statuses (dedifferentiation, transdifferentiation; Fig. 5), depicting a dynamic (wobbling) landscape that does not inevitably entail progressive loss of stemness. Thus, when a cell ‘makes a decision’, the subsequent journey is not bound by this decision. Importantly, in this model, there is no need for the existence of any ASC niches.

The wobbling Penrose landscape diverges conceptually from the Waddingtonian landscape in three key ways: (*i*) there is no bifurcation ‘choice’, or travelling along symbolic valleys, as in the Waddingtonian landscape, which is subject to a gravity force. The Penrose landscape is a gravity‐independent construct, allowing continual gradients of cellular potency, without any predetermined decision. (*ii*) The likelihood of backward/sideways trajectories in the Waddingtonian landscape has rarely been raised in the literature (e.g. Pesaresi, Sebastian‐Perez & Cosma, 2019) as, conceptually, such processes necessitate invested energy. In the wobbling Penrose landscape, stem cells, progenitors and even fully differentiated cells at any level of stemness status can move up or change stemness position (Fig. 5). (*iii*) There is no single downward route in the potency slope but, instead, multiple trajectories of cellular potency can emerge.

The vertebrate literature also reveals cases more in keeping with the wobbling Penrose than the Waddingtonian unidirectional landscape (e.g. Furusawa & Kaneko, 2012; Clevers, 2015; Sieweke, 2015; Kholodenko & Yarygin, 2017; Buczacki, 2019), including ASCs that are not endowed with a determined fate while subjected to stochastic events (Clevers, 2015; Post & Clevers, 2019) or cases of ‘stem cell plasticity’ (Loeffler & Roeder, 2002; Poulsom *et al*., 2002; Raff, 2003; Wagers & Weissman, 2004; Chacón‐Martínez *et al*., 2018) where committed stem cells differentiate or transdifferentiate into different cell lineages. While we do not review the vertebrate literature extensively here, such putative cases fitting a wobbling landscape may commonly exist.

As in the vertebrates, ASCs in invertebrates maintain lineages, replace cell losses caused by wear‐and‐tear, and regulate between quiescence and proliferation. Yet, in many invertebrate taxa, stemness is further associated with (*i*) sets of responses to environmental assaults (e.g. whole‐body regeneration, dormancy), or ecotoxicological impacts (Rosner *et al*., 2021). (*ii*) novel biological traits expressed irrespective of environmental cues (e.g. budding, fission, fragmentation), (*iii*) innate immunity (Ballarin *et al*., 2021), and (*iv*) indeterminate growth [e.g. sponges, cnidarians, annelids (e.g. atokous worm stage), tunicates (Jackson & Coates, 1986; Hughes, 1987; Gazave *et al*., 2013)], a largely neglected trait as the conventional models in stem cell research follow determinate growth plans (Vogt, 2012). Along this line of acquired traits, aquatic invertebrate ASCs not only demonstrate a higher fidelity of stem cell renewal, even when compared with tumorigenesis (Robert, 2010; Vogt, 2012; Tascedda & Ottaviani, 2014), but in some cases, are also elevated to the level of legitimate units of selection (Buss, 1982; Rinkevich, 2000, 2009, 2011; Weissman, 2000; Fields & Levin, 2018).

## VIII. CONCLUSIONS

(1) The current paradigm suggests the lifelong existence of adult stem cells (ASCs) in Metazoa. In vertebrates, ASCs are defined as lineage‐restricted cells, limited to tissue or organ‐specific activities, that are capable of regulating homeostasis, repair and regeneration of tissues and organs. While during early embryogenesis stem cells in vertebrates are totipotent and then pluripotent, post‐embryonic ASCs are multipotent at best. It is widely accepted that vertebrate ASCs are rare, clonogenic, undifferentiated, and often express specific ‘stemness’ genes. They are capable of self‐renewal and multilineage differentiation, often interacting with specialized stem cell niches, and are considered slow‐cycling cells that show distinct germ/somatic lineage potential. They function in homeostasis and, with constraints, in the regeneration of organs/tissues.

(2) Numerous key ASC traits in invertebrates differ from those assigned to ASCs of vertebrates. Fifteen such traits are highlighted herein, revealing a wide range of disparate characteristics from morphology, differentiation states and somatic/germ lineage characteristics, to some essential biological properties and roles. Numerous predominantly marine phyla (e.g. Porifera, Cnidaria, Ctenophora, Annelida, Acoela, Platyhelminthes, Echinodermata, Cephalochordata and Tunicata) possess large pools of *bona fide* ASCs throughout the lifespan of the organism (sometimes consisting of up to 40% of all animals' cells), most of which are multipotent, pluripotent and even totipotent, with high differentiation potential that contribute to more than a single germ layer. They may arise *de novo* by transdifferentiation from somatic cells and even from germ cells, with no signature of germ‐cell sequestration, and are key players in phenomena such as whole‐body regeneration, asexual budding and dormancy. Many invertebrate ASCs consist of epithelial tissues, exhibiting epithelial cell hallmarks with distinct apical–basal and planar cell polarities, apical cell–cell junctions, and basal cell–extracellular matrix interactions, all of which are features of differentiated cells.

(3) ASCs in invertebrates represent a wide range of phylum‐specific and characteristic cell types, morphologies and behaviours, ranging from sponge archaeocytes and choanocytes, hydrozoan i‐cells, platyhelminth or acoel neoblasts to tunicate haemoblasts. Even within phyla, comparisons reveal a considerable degree of additional variation, where ASC properties are possessed by only particular taxa within a phylum. In the same way, ASC lineages and progenitors may show intra‐phylum specializations.

(4) Invertebrate ASCs express orthologues of many vertebrate ‘stemness’ genes, as well as genes that contribute to cancer cell ‘stem cell potential’. However, it is challenging to identify let alone compare stemness gene signatures across diverse invertebrate taxa spanning wide evolutionary distances. The molecular mechanisms by which invertebrates hold viable ASC stocks, with long‐term stability and constant proliferation during their lifespan, remain elusive. In addition, the essence of ASC stemness in marine invertebrates cannot be distilled down to a single shared molecular fingerprint. Also, in contrast to the vertebrates, somatic and germline stemness markers (e.g. *Vasa*, *Pl10*, *Piwi*, *Nanos*, *Bruno*, *Pumilio*, *Tudor*, etc.) are co‐expressed in differentiated somatic cells/tissues in many invertebrate phyla.

(5) While knowledge gained from mammalian, *D. melanogaster* and *C. elegans* models provide guidelines for defining comparable niches in other metazoans, studies on sponge archaeocytes and choanocytes, hydrozoan i‐cells and platyhelminth and acoel neoblasts have failed to define either discrete anatomical microenvironments where stem cells reside, or a niche‐specific extracellular matrix to which ASCs home. In hydrozoans and planarians, studies further view the whole animal or tissue as a single functional stem cell niche. Botryllid ascidians, by contrast, reveal a different scenario relative to other taxa, with putative ASCs homing to discrete, yet ephemeral, microenvironments.

(6) Beyond their functions in supporting homeostasis, ASCs in many metazoans also play major roles in supporting key biological processes such as regeneration in adults, including whole‐body regeneration, agametic asexual reproduction such as budding and fission, indeterminate growth, postponed ageing and dormancy phenomena.

(7) Conceptualizing the above disparities, we present an alternative stemness metaphor to the Waddington landscape, termed the ‘wobbling Penrose’ landscape. In this metaphor, totipotent ASCs adopt ascending/descending courses of an ‘Escherian stairwell’, in a lifelong totipotency pathway. ASCs may also travel along lower stemness echelons to reach fully differentiated states. However, from any starting state, cells can change their stemness status, underscoring their dynamic cellular potencies. Thus, vertebrate ASCs may reflect just one metazoan ASC archetype.

## IX ACKNOWLEDGEMENTS

This study is based upon work from COST Action 16203 ‘Stem cells of marine/aquatic invertebrates: from basic research to innovative applications’ (MARISTEM), supported by COST (European Cooperation in Science and Technology). The idea for this review was initially discussed by the Action core group (B. R., B. H., L. B., P. M., I. S.) and developed during a specific workshop entitled ‘Adult stem cells (ASC) from marine/aquatic invertebrates’ held at the Obergurgl Center of the University of Innsbruck from March 29 to 31, 2019. B. R. is supported by a grant from the United States‐Israel Binational Science Foundation (BSF no. 2015012), Jerusalem, Israel. E. R. is supported by funding from the French Government (National Research Agency, ANR) through the ‘Investments for the Future’ programs IDEX UCAJedi (ANR‐15‐IDEX‐01) as well as the grant RENEW (ANR‐19‐PRC). B. H. is supported by the Marie Skłodowska‐Curie COFUND program ARDRE ‘Ageing, Regeneration and Drug Research’ (research grant nr. 847681).

## X AUTHOR CONTRIBUTIONS

B. R. conceived the idea and wrote the first version of the manuscript. B. H., L. B., P. M. and I. S. further developed the idea and coordinated analyses. E. G., O. P., M. S., I. S., P. M., L. B., B. H., A. E. and D. K. actively collected and analysed the literature. B. R., B. H., L. B., P. M., I. S. and O. B.‐H. discussed and created Fig. 5. All co‐authors read and commented on drafts, and approved the final version.

## Supporting information


**Fig. S1.** Diversity of adult stem cell (ASC) contributions to four major biological processes in the Cnidaria: homeostasis, dormancy, regeneration and agametic asexual reproduction. The presence of the biological process, involvement of undifferentiated/differentiated putative ASCs or progenitors and their level of potency, as well as the specific classes of stemness gene families they express are mapped for major cnidarian lineages. Circles: empty circle – documented presence of the biological process; filled circle – cases where putative ASCs or progenitors are involved. A red cross signifies the absence of the biological process in the lineage as currently documented. As homeostasis is a property of life, all groups are shown with an empty circle. For adult regeneration, the asterisk documents the presence of whole‐body regeneration. Dormancy refers to any documented type of dormant stage or torpor‐like process and has likely evolved independently in each lineage. For dormancy, A – quiescence, diapause, growth/degrowth; G – growth/degrowth; O – ontogeny reversal; Q – quiescence. For agametic asexual reproduction, B – any form of budding, F – any form of fission/fragmentation. Triangles indicate the level of documented potency for ASCs: red = lineage restricted/unipotent; cyan = totipotent; blue = multi/pluripotent; gradient triangle = documented cases of several ASCs or progenitors with different potency. Selected stemness gene families whose members are expressed in ASCs during the biological process are listed in a box for each process and group where known. The relative contribution of undifferentiated (U) *versus* differentiated (D) ASCs or progenitors within each subclass is mapped onto the phylogeny where known; levels of confidence are represented by solid (higher) and dotted (lower) diamonds, while the sizes of D and U reflect their presumed level of contribution. A hypothetical ancestral state for this character is proposed at the corresponding node of the simplified cnidarian phylogenetic tree. A general consensus for all features across Cnidaria is proposed at the top of the figure. Key species for which data exist in each subclass are named. Data are derived from Tables [Link], [Link].

**Fig. S2.** Diversity of adult stem cell (ASC) contributions to four major biological processes in the Echinodermata: homeostasis, dormancy, regeneration and agametic asexual reproduction. The presence of the biological process, involvement of undifferentiated/differentiated putative ASCs or progenitors and their level of potency, as well as the specific classes of stemness gene families they express are mapped for major echinoderm lineages. Circles: empty circle – documented presence of the biological process; filled circle – cases where putative ASCs or progenitors are involved. A red cross signifies the absence of the biological process in the lineage as currently documented. As homeostasis is a property of life, all groups are shown with an empty circle. For adult regeneration, the asterisk documents the presence of whole‐body regeneration. Dormancy refers to any documented type of dormant stage or torpor‐like process and has likely evolved independently in each lineage. For dormancy, the dotted line circle indicates potential involvement in the respective biological feature in non‐adults. Q – quiescence. For agametic asexual reproduction, F – any form of fission/fragmentation. Triangles indicate the level of documented potency for ASCs (filled) and progenitors (empty). Red = lineage restricted/unipotent; blue = multi/pluripotent; gradient triangle = documented cases of several ASCs or progenitors with different potency. Selected stemness gene families whose members are expressed in ASCs or progenitors during the biological process are listed in a box for each process and group where known. The relative contribution of undifferentiated (U) *versus* differentiated (D) ASCs or progenitors within each class is mapped onto the phylogeny; levels of confidence are represented by solid (higher) and dotted (lower) diamonds, while the sizes of D and U reflect their presumed level of contribution. A hypothetical ancestral state for this character is proposed at the corresponding node of the simplified echinoderm phylogenetic tree. A general consensus for all features across Echinodermata is proposed at the top of the figure. Key species for which data exist in each class are named. Data are derived from Tables [Link], [Link].

**Table S1.** Properties of selected, well‐studied adult stem cell (ASC) lineages in invertebrates.

**Table S2.** Genes expressed in invertebrate adult stem cell (ASCs) and progenitor cells during potency state changes.

**Table S3.** Suggested stem cell niches (SCNs) present in invertebrates.

**Table S4.** Overview of the involvement of adult stem cell (ASCs) and progenitors during homeostasis in metazoans.

**Table S5.** Overview of the involvement of adult stem cell (ASCs) and progenitors in regeneration processes in metazoans.

**Table S6.** Overview of the involvement of adult stem cell (ASCs) and progenitors in agametic asexual reproduction (budding, fission/fragmentation) in metazoans.

**Table S7.** Overview of the involvement of adult stem cell (ASCs) and progenitors in dormancy in metazoans.
