# Supplementary figures and images for: A pan‐metazoan concept for adult stem cells: the wobbling Penrose landscape
Source: Biol Rev Camb Philos Soc. 2021 Oct 6;97(1):299–325. doi: 10.1111/brv.12801 (PMC9292022; doi:10.1111/brv.12801)

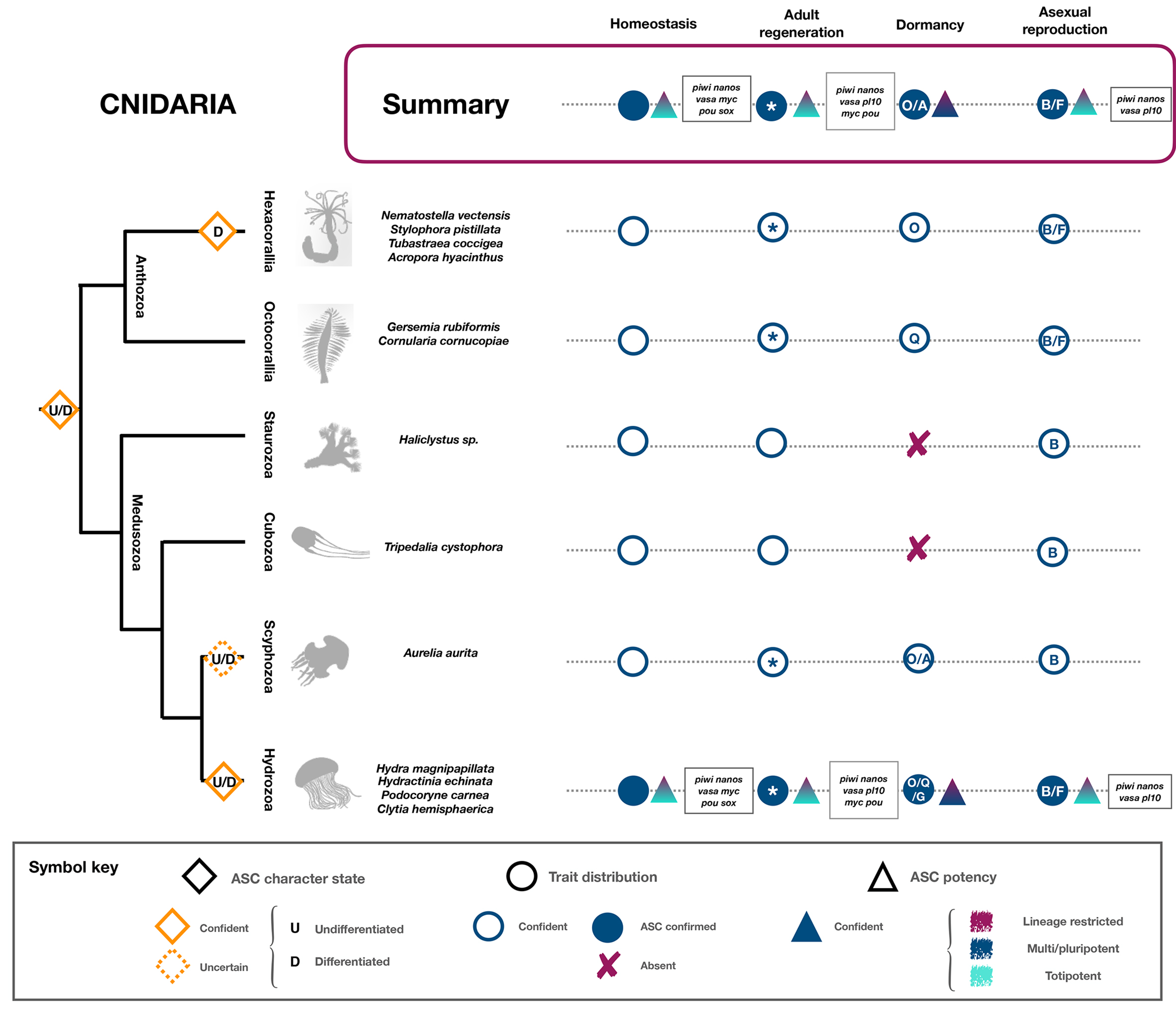

Supplement: Supplementary file 1 — Fig. S1. Diversity of adult stem cell (ASC) contributions to four major biological processes in the Cnidaria: homeostasis, dormancy, regeneration and agametic asexual reproduction. The presence of the biological process, involvement of undifferentiated/differentiated putative ASCs or progenitors and their level of potency, as well as the specific classes of stemness gene families they express are mapped for major cnidarian lineages. Circles: empty circle – documented presence of the biological process; filled circle – cases where putative ASCs or progenitors are involved. A red cross signifies the absence of the biological process in the lineage as currently documented. As homeostasis is a property of life, all groups are shown with an empty circle. For adult regeneration, the asterisk documents the presence of whole‐body regeneration. Dormancy refers to any documented type of dormant stage or torpor‐like process and has likely evolved independently in each lineage. For dormancy, A – quiescence, diapause, growth/degrowth; G – growth/degrowth; O – ontogeny reversal; Q – quiescence. For agametic asexual reproduction, B – any form of budding, F – any form of fission/fragmentation. Triangles indicate the level of documented potency for ASCs: red = lineage restricted/unipotent; cyan = totipotent; blue = multi/pluripotent; gradient triangle = documented cases of several ASCs or progenitors with different potency. Selected stemness gene families whose members are expressed in ASCs during the biological process are listed in a box for each process and group where known. The relative contribution of undifferentiated (U) versus differentiated (D) ASCs or progenitors within each subclass is mapped onto the phylogeny where known; levels of confidence are represented by solid (higher) and dotted (lower) diamonds, while the sizes of D and U reflect their presumed level of contribution. A hypothetical ancestral state for this character is proposed at the corresponding node of the [file BRV-97-299-s007.jpg]

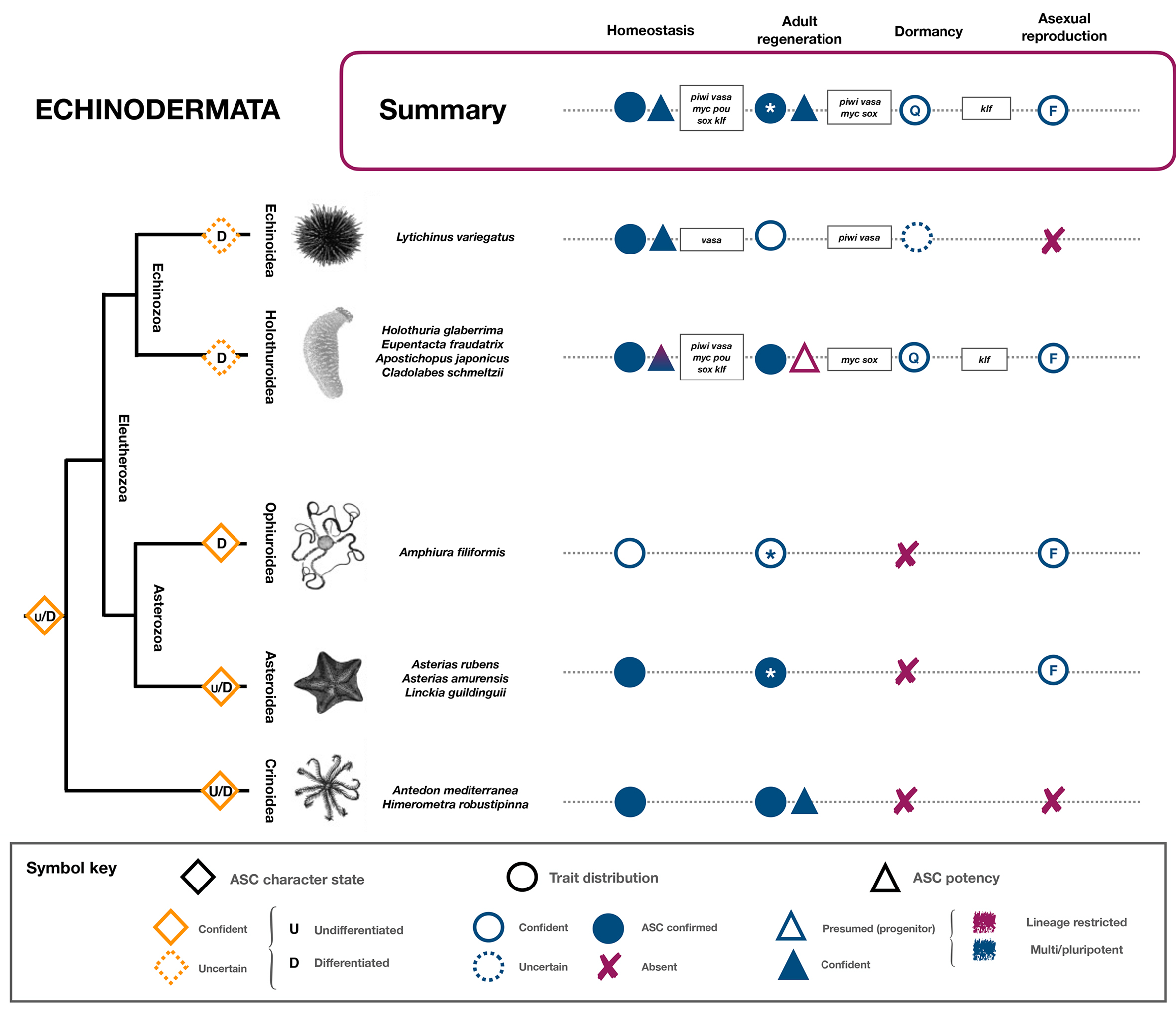

Supplement: Supplementary file 2 — Fig. S2. Diversity of adult stem cell (ASC) contributions to four major biological processes in the Echinodermata: homeostasis, dormancy, regeneration and agametic asexual reproduction. The presence of the biological process, involvement of undifferentiated/differentiated putative ASCs or progenitors and their level of potency, as well as the specific classes of stemness gene families they express are mapped for major echinoderm lineages. Circles: empty circle – documented presence of the biological process; filled circle – cases where putative ASCs or progenitors are involved. A red cross signifies the absence of the biological process in the lineage as currently documented. As homeostasis is a property of life, all groups are shown with an empty circle. For adult regeneration, the asterisk documents the presence of whole‐body regeneration. Dormancy refers to any documented type of dormant stage or torpor‐like process and has likely evolved independently in each lineage. For dormancy, the dotted line circle indicates potential involvement in the respective biological feature in non‐adults. Q – quiescence. For agametic asexual reproduction, F – any form of fission/fragmentation. Triangles indicate the level of documented potency for ASCs (filled) and progenitors (empty). Red = lineage restricted/unipotent; blue = multi/pluripotent; gradient triangle = documented cases of several ASCs or progenitors with different potency. Selected stemness gene families whose members are expressed in ASCs or progenitors during the biological process are listed in a box for each process and group where known. The relative contribution of undifferentiated (U) versus differentiated (D) ASCs or progenitors within each class is mapped onto the phylogeny; levels of confidence are represented by solid (higher) and dotted (lower) diamonds, while the sizes of D and U reflect their presumed level of contribution. A hypothetical ancestral state for this character is proposed at the correspondi [file BRV-97-299-s008.jpg]
