# Supplementary material for: A pan‐metazoan concept for adult stem cells: the wobbling Penrose landscape
Source: Biol Rev Camb Philos Soc. 2021 Oct 6;97(1):299–325. doi: 10.1111/brv.12801 (PMC9292022; doi:10.1111/brv.12801)
Supplement: Supplementary file 3 — Table S1. Properties of selected, well‐studied adult stem cell (ASC) lineages in invertebrates. [file BRV-97-299-s005.docx]

**Table S1.** Properties of selected, well-studied adult stem cell (ASC) lineages in invertebrates. A summary of the properties of major aquatic invertebrate ASC lineages studied in depth at the cellular and molecular levels. Presented are the best-studied ASC lineages from sponges, cnidarians, flatworms, acoelomorphs, and tunicates. These ASC lineages provide the basis for adult tissue replacement and contribute to injury-induced regeneration processes. Exceptions are sponge archaeocytes and pinacocytes, for which detailed data have been collected under conditions of regeneration, while data on tissue homeostasis are limited. In addition, tunicate bud primordium cells have not yet been tested for their potential role in regeneration. Tracing the ontogenetic descent (lineage tracing) from embryonic or larval tissues is available for most ASC lineages. Furthermore, *de novo* formation of ASCs by trans- or dedifferentiation of cells of a different lineage has been demonstrated to occur in sponges and hydrozoans. Potency is defined according to guidelines provided by the NIH (*https://stemcells.nih.gov/glossary.htm*). Aquatic ASCs exhibit multi- or pluripotency. Totipotency has been proposed for a few lineages, i.e. for interstitial stem cells in the hydrozoan *Hydractinia echinata* and bud primordium cells in the colonial tunicate *Botryllus schlosseri*. However, in these cases unambiguous proof still does not exist. Despite rather rigorous testing, no evidence for senescence has been found in hydrozoan ASCs, and this has been attributed to asexual life-cycle traits. Most bilaterian aquatic invertebrate ASCs exhibit cellular ageing. Senescence in sponge ASCs has thus far not been studied in sufficient detail. Finally, we list the major experimental approaches taken to analyse aquatic invertebrate ASC lineages. Due to their accessibility in well-established laboratory models, planarian neoblasts, hydrozoan interstitial stem cells and tunicate haemoblasts can be studied using a wide array of techniques. In other ASC lineages, methods such as stem cell cloning and sophisticated molecular tools need to be developed. Statements generally refer to all the listed species of a given taxon. Colour-coding is used in cases of ASC lineages displaying different characters between and within classes of a given taxon. The coloured numbers of references refer to the respective specific coloured species names.

| **Phylum** | **Class (*Species*)** | **ASC lineage** | **Ontogenetic origin** | ***de novo* formation** | **Potency** | **Senescence** | **Experimental validation** | | **Reference** |
| --- | --- | --- | --- | --- | --- | --- | --- | --- | --- |
| **PORIFERA** |  |  |  |  |  |  | |  |  |
|  | Demospongiae (*Ephydatia fluviatilis*, *Halisarca dujardini*, *Amphimedon queenslandica*) | archaeocyte | from larval archaeocytes and ciliated cells^1,2^ | from gemmule thesocytes and from choanocytes^3–6^ | pluripotent ^1–6^ | not rigorously tested | | regeneration studies^3,4^; expression of stem cell markers^5,7,8^ | 1–8 |
|  | Demospongiae (*Ephydatia fluviatilis*, *Halisarca dujardini*, *Amphimedon queenslandica*); Homoscleromorpha (*Oscarella lobularis*); Calcarea (*Leucosolenia* sp., *Sycon* sp.) | choanocyte | from larval ciliated cells^1,2,9,10^ | from archaeocytes^5–6,11^; from pinacocytes^11,12,13,14^ | pluripotent^1,2,5–7,11,12,13,14^ | not rigorously tested | | regeneration studies^6,13^; lineage tracing^5^; expression of stem cell markers^5,7,8,12^ | 1, 2, 5–14 |
|  | Homoscleromorpha (*Oscarella lobularis*); Calcarea (*Leucosolenia* sp., *Sycon* sp*.*) | pinacocyte | from larval ciliated cells ^9,10^ | from choanocytes^11,13,14^ | multipotent^9,10,11,13,14^ | not rigorously tested | | regeneration studies^11,14^ | 9–11,  13, 14 |
| **CNIDARIA** |  |  |  |  |  |  | |  |  |
|  | Hydrozoa (*Hydractinia* spp., *Hydra vulgaris*) | interstitial cell | from embryonic endoderm^15^ | not observed | pluripotent^16,17^; multipotent^18–22^ | not observed | | regeneration studies^23,24^; stem cell cloning^18^; lineage tracing^16,22^; expression of stem cell markers^25–27^; genetic interference^28^ | 15–25,  27,  28 |
|  | Hydrozoa (*Hydractinia* spp*.*, *Hydra vulgaris*) | ectodermal and endodermal epitheliomuscular cell | from embryonic ecto- and endoderm^21^ | from interstitial cells^17^; *de novo* formation not observed in *Hydra* | multipotent^20,21^ | not observed | | regeneration studies^24^; lineage tracing^29^; expression of stem cell marker genes^26,30^ | 17, 20,  21, 24,  26, 29,  30 |
| **PLATYHELMINTHES** |  |  |  |  |  |  | |  |  |
|  | Rhabditophora (*Schmidtea mediterranea*, *Macrostomum lignano*) | neoblast | piwi-1 positive blastomeres^31^ | not observed | pluripotent^32,33,34^ | yes | | regeneration studies^32,35^; stem cell cloning^34^; lineage tracing^33,36,37^; expression of stem cell markers^33,36,37^; genetic interferences^33,36,37^ | 31–37 |
| **ACOELOMORPHA** |  |  |  |  |  |  | |  |  |
|  | Acoela (*Isodiametra pulchra*) | neoblast | not known | not observed | pluripotent^38,39^ | yes | | lineage tracing^39,40^; expression of stem cell markers^39,40^; genetic interference^39^ | 38–40 |
| **UROCHORDATA** |  |  |  |  |  |  | |  |  |
|  | Ascidiacea (*Ciona robusta*, *Botryllus schlosseri*) | haemoblast | from larval mesenchymal cells | not observed | multipotent^41,43,46^ | yes^42^; not observed^43^ | | lineage tracing^41,42,44,45^; expression of stem cell-markers^1,2,43–46^; stem cell cloning^43,44^; genetic interference^45^ | 1, 2, 41–46 |
|  | Ascidiacea (*Botrylloides diegensis*) | haemoblast | not known | not observed | pluripotent^47^ | not observed | | expression of stem cell markers; functional analyses; rescue experiment^47^ | 47 |
|  | Ascidiacea (*Botryllus schlosseri*) | bud primordium epithelial cell | from embryonic ectoderm | not observed | pluripotent^48,49^ | yes | | histological analysis^48,49^ | 48, 49 |

**References**

1. Wielsputz C, Saller U. 1990. The metamorphosis of the parenchymula-larva of *Ephydatia fluviatilis* (Porifera, Spongillidae). Zoomorphology. 109:173-177.

2. Ereskovsky AV, Konjukov P, Willenz P. 2007*a*. Experimental metamorphosis of *Halisarca dujardini* larvae (Demospongiae, Halisarcida): Evidence of flagellated cell totipotentiality. J Morph. 268:529-536.

3. De Sutter D, Van de Vyver G. 1977. Aggregative properties of different cell types of fresh-water sponge *Ephydatia fluviatilis* isolated on ficoll gradients. Roux's Arch Dev Biol. 181:151-161.

4. Buscema M, De Sutter D, Van de Vyver, G. 1980. Ultrastructural study of differentiation processes during aggregation of purified sponge archaeocytes. Wilhelm Roux Arch Dev Biol. 188:45–53.

5. Funayama N, Nakatsukasa M, Mohri K, Masuda Y, Agata K. 2010. Piwi expression in archeocytes and choanocytes in demosponges: insights into the stem cell system in demosponges. Evol Dev. 12:275–287.

6. Borisenko IE, Adamska M, Tokina DB, Ereskovsky AV. 2015. Transdifferentiation is a driving force of regeneration in *Halisarca* dujardini (Demospongiae, Porifera). Peer J. 3:e1211.

7. Sogabe S, Hatleberg WL, Kocot KM, Say TE, Stoupin D, Roper KE, Fernandez-Valverde SL, Degnan SM, Degnan BM. 2019. Pluripotency and the origin of animal multicellularity. Nature. 570:519-522.

8. Alié A, Hayashi T, Sugimura I, Manuel M, Sugano W, Mano A, Satoh N, Agata K, Funayama N. 2015. The ancestral gene repertoire of animal stem cells. Proc Natl Acac Sci USA. 112:E7093-E7100.

9. Ereskovsky AV, Tokina DB, Bezac C, Boury-Esnault N. 2007*b*. Metamorphosis of cinctoblastula larvae (Homoscleromorpha, Porifera). J Morph. 268:518-528.

10. Amano S, Hori I. 1993. Metamorphosis of calcareous sponges. II. Cell rearrangement and differentiation in metamorphosis. Inv Reprod Dev. 24:13-26.

11. Ereskovsky AV, Borisenko IE, Lapebie P, Gazave E, Tokina DB, Borchiellini C. 2015. *Oscarella lobularis* (Homoscleromorpha, Porifera) regeneration: Epithelial morphogenesis and metaplasia. PLoS ONE. 10:e0134566.

12. Fierro-Constain L, Schenkelaars Q, Gazave E, Haguenauer A, Rocher C, Ereskovsky A, Borchiellini C, Renard E. 2017. Conservation of the germline multipotency program, from sponges to vertebrates: A stepping stone to understanding the somatic and germline origins. Genome Biol Evol. 9:474-488.

13. Lavrov AI, Bolshakov FV, Tokina DB, Ereskovsky AV. 2018. Sewing wounds up: the epithelial morphogenesis as a central mechanism of calcaronean sponge regeneration. J Exp Zool Part B: Mol Dev Evol. 330:351-371.

14. Korotkova GP. 1972. Regeneration of the calcareus sponge *Sycon lingua*. Trans Leningrad Soc Nat. 78:155-171.

15. Weiler-Stolt B. 1960. Über die Bedeutung der interstitiellen Zellen für die Entwicklung und Fortpflanzung mariner Hydrozoen. Wilhelm Roux’s Arch Dev Biol. 152:398-455.

16. Plickert G, Frank U, Müller WA. 2012. *Hydractinia*, a pioneering model for stem cell biology and reprogramming somatic cells to pluripotency. Int J Dev Biol. 56:519-534.

17. Müller WA, Teo R, Frank U. 2004. Totipotent migratory stem cells in a hydroid. Dev Biol. 275:215-224.

18. Bosch TCG, David CN. 1987. Stem cells of *Hydra magnipapillata* can differentiate into somatic cells and germ line cells. Dev Biol. 121:182-191.

19. Bode HR. 1996. The interstitial cell lineage in *Hydra*: a stem cell system that arose early in evolution. J Cell Sci. 109:1155-1164.

20. Bosch TCG, Anton-Erxleben F, Hemmrich G, Khaltutrin K. 2010. The *Hydra* polyp: Nothing but an active stem cell community. Dev Growth Differ. 52:15-25.

21. Hobmayer B, Jenewein M, Eder D, Eder M-K, Glasauer S, Gufler S, Hartl M, Salvenmoser W. 2012. Stemness in *Hydra* - a current perspective. Int J Dev Biol. 56:509-517.

22. David CN. 2012. Interstitial stem cells in *Hydra*: multi-potency and decision making. Int J Dev Biol. 56:489-497.

23. Gahan JM, Bradshaw B, Flici H, Frank U. 2016. The interstitial stem cells in *Hydractinia* and their role in regeneration. Curr Op Genet Dev. 40:65-73.

24. Vogg MC, Galliot B, Tsiairis CD. 2019. Model systems for regeneration: *Hydra*. Development. 146:dev177212.

25. Watanabe H, Hoang VT, Mättner R, Holstein TW. 2009. Immortality and the base of multicellular life: Lessons from cnidarian stem cells. Sem Cell Dev Biol. 20:1114-1125.

26. Hemmrich G, Khalturin K, Boehm A-M, Puchert M, Anton-Erxleben F, Wittlieb J, Klostermeier UC, Rosnestiel P, Oberg H-H, Domazet-Loso T, et al. 2012. Molecular signatures of the three stem cell lineages in *Hydra* and the emergence of stem cell function at the base of multicellularity. Mol Biol Evol. 29:3267-3280.

27. Siebert S, Farrell JA, Cazet JF, Abeykoon Y, Primack AS, Schnitzler CE, Juliano CE. 2019. Stem cell differentiation trajectories in *Hydra* resolved at single-cell resolution. Science. 365:eaav9314.

28. Boehm AM, Khalturin K, Anton-Erxleben F, Hemmrich G, Klostermeier UC, Lopez-Quintero JA, Oberg HH, Puchert M, Rosenstiel P, Wittlieb J, et al. 2012. FoxO is a critical regulator of stem cell maintenance in immortal *Hydra*. PNAS. 109:19697-19702.

29. Wittlieb J, Khalturin K, Lohmann JU, Anton-Erxleben F, Bosch TCG. 2006. Transgenic *Hydra* allow in vivo tracking of individual stem cells during morphogenesis. PNAS. 103:6208-6211.

30. Buzgariu W, Al Haddad S, Tomczyk S, Wenger Y, Galliot B. 2015. Multi-functionality and plasticity characterize epithelial cells in *Hydra*. Tissue Barriers. 3:e1068908.

31. Davies EL, Lei K, Seidel CW, Kroesen AE, McKinney SA, Guo L, Mc Robb S, Ross EJ, Gotting K, Sánchez Alvarado A. 2017. Embryonic origin of adult stem cells required for tissue homeostasis and regeneration. eLife 6:e21052.

32. Reddien PW. 2018. The cellular and molecular basis for planarian regeneration. Cell. 175:327-345.

33. Wudarski J, Simanov D, Ustyantsev K, de Mulder K, Grelling M, Grudniewska M, Beltman F, Glazenburg L, Demircan T, Wunderer J, et al. 2017. Efficient transgenesis and annotated genome sequence of the regenerative flatworm model *Macrostomum lignano*. Nat Commun. 8:2120.

34. Wagner DE, Wang IE, Reddien PW. 2011. Clonogenic neoblasts are pluripotent adult stem cells that underlie planarian regeneration. Science. 332:811-816.

35. De Mulder K, Pfister D, Kuales G, Egger B, Salvenmoser W, Willems M, Steger J, Fauster K, Micura R, Borgonie G, et al. 2009*b*. Stem cells are differentially regulated during development, regeneration and homeostasis in flatworms. Dev Biol. 334:198-212.

36. Fincher CT, Wurtzel O, de Hoog T, Kravarik KM, Reddien PW. 2018. Cell type transcriptome atlas for the planarian *Schmidtea mediterranea*. Science. 360:eaaq1736.

37. Plass M, Solana J, Wolf FA, Ayoub S, Misios A, Glažar P, Obermayer B, Theis FJ, Kocks C, Rajewsky N. 2018. Cell type atlas and lineage tree of a whole complex animal by single-cell transcriptomics. Science. 360:eaaq1723.

38. Bely AE, Sikes JM. 2010*a*. Acoel and platyhelminth models for stem-cell research. J Biol. 9:14.

39. De Mulder K, Kuales G, Pfister D, Willems M, Egger B, Salvenmoser W, Thaler M, Gorny AK, Hrouda M, Borgonie G, et al. 2009*a*. Characterization of the stem cell system of the acoel *Isodiametra pulchra*. BMC Dev Biol. 9:69.

40. Egger B, Steinke D, Tarui H, De Mulder K, Arendt D, Borgonie G, Funayama N, Gschwentner R, Hartenstein V, Hobmayer B, et al. 2009*b*. To be or not to be a flatworm: the acoel controversy. PLoS One. 4:e5502.

41. Jeffery WR. 2019. Progenitor targeting by adult stem cells in *Ciona* homeostasis, injury, and regeneration. Dev Biol. 448:279-290.

42. Jeffery WR. 2015*b*. Regeneration, stem cells, and aging in the tunicate *Ciona*: Insights from the oral siphon. Int Rev Cell Mol Biol. 319:255-282.

43. Laird DJ, De Tomaso AW, Weissman IL. 2005. Stem cells are units of natural selection in a colonial ascidian. Cell. 123:1351-1360.

44. Voskoboynik A, Soen Y, Rinkevich Y, Rosner A, Ueno H, Reshef R, Ishizuka KJ, Palmeri KJ, Moiseeva E, Rinkevich B, et al. 2008. Identification of the endostyle as a stem cell niche in a colonial chordate. Cell Stem Cell. 3:456-464.

45. Rinkevich Y, Voskoboynik A, Rosner A, Rabinowitz C, Paz G, Oren M, Douek J, Alfassi G, Moiseeva E, Ishizuka KJ, et al. 2013. Repeated, long-term cycling of putative stem cells between niches in a basal chordate. Dev Cell. 24:76-88.

46. Rosental B, Kowarsky M, Seita J, Corey DM, Ishizuka KJ, Palmeri KJ, Chen SY, Sinha R, Okamoto J, Mantalas G, et al. 2018. Complex mammalian-like haematopoietic system found in a colonial chordate. Nature. 564:425-429.

47. Kassmer SH, Langenbacher AD, De Tomaso AW. 2019. Integrin-alpha-6+ candidate stem cells are responsible for whole body regeneration in the invertebrate chordate *Botrylloides diegensis*. Nat Commun. 7(11):4435.

48. Manni L, Zaniolo G, Cima F, Burighel P, Ballarin L. 2007. *Botryllus schlosseri*: a model ascidian for the study of asexual reproduction. Dev Dyn. 236:335-352.

49. Manni L, Anselmi C, Cima F, Gasparini F, Voskoboynik A, Martini M, Peronato A, Burighel P, Zaniolo G, Ballarin L. 2019. Sixty years of experimental studies on the blastogenesis of the colonial tunicate *Botryllus schlosseri*. Dev Biol. 448:293-308.
