# Supplementary material for: A pan‐metazoan concept for adult stem cells: the wobbling Penrose landscape
Source: Biol Rev Camb Philos Soc. 2021 Oct 6;97(1):299–325. doi: 10.1111/brv.12801 (PMC9292022; doi:10.1111/brv.12801)
Supplement: Supplementary file 4 — Table S2. Genes expressed in invertebrate adult stem cell (ASCs) and progenitor cells during potency state changes. [file BRV-97-299-s004.docx]

**Table S2.** Genes expressed in invertebrate adult stem cells (ASCs) and progenitor cells during potency state changes. On the basis of the codified proteins, genes were grouped as: genes coding for: RNA-binding proteins, RNA recognition motif (RRM)-containing proteins, signal transduction proteins, transcription factors, chromatin modification/cell cycle/ proteins, proteins involved in autophagy, control of differentiation proteins, niche interaction proteins and genes for microRNAs (miRNAs). Each category of genes (titles in boxes with green background) can contain various subgroups (titles in boxes with blue background). Within each subgroup, genes are listed according to the phylogenetic position of the organisms.

| **Species** | **Gene** | **Cell types** | **Tissue/organ** | **Interval of expression** | **Identification methods** | **Reference** |
| --- | --- | --- | --- | --- | --- | --- |
| **RNA-binding proteins** | | | | | | |
| ***Argonaute family silencing genes*** | | | | | | |
| *Ephydatia fluviatilis* (Porifera, Demospongiae) | *EfpiwiA*  *EfpiwiB* | choanocytes  archaeocytes | choanocyte chambers  mesohyl | throughout ontogeny | ISH, scRNAseq | 1, 2 |
| *Oscarella lobularis* (Porifera, Homoscleromorpha) | *OlpiwiA* | choanocytes  pinacocytes  vacuolar cells type 1 | choanocyte chambers  pinacoderm  mesohyl | throughout ontogeny  during wound healing  during wound healing | ISH | 3 |
|  | *OlpiwiB*  *Olago* | choanocytes  pinacocytes  vacuolar cells type 1 | choanocyte chambers  pinacoderm  mesohyl | throughout ontogeny | ISH | 3 |
| *Podocoryne carnea* (Cnidaria, Hydrozoa) | *Cniwi* | epitheliomuscular cells | epiderm | throughout ontogeny | ISH | 4, 5 |
| *Hydra vulgaris* (Cnidaria, Hydrozoa) | *Hywi*  *Hyli* | i-cells  nematoblasts  epidermal cells  gastrodermal cells | body column | throughout ontogeny | ISH, IB | 6 |
| *Hydra magnipapillata* (Cnidaria, Hydrozoa) | *Hywi*  *Hyli* | i-cells  epidermal cells | body column | throughout ontogeny | ISH, IB | 6 |
| *Nanomia bijuga* (Cnidaria, Hydrozoa, Siphonophora) | *piwi* | i-cells (epiderm and gastroderm) | siphonosomal horn  buds  young zooids | throughout ontogeny | ISH | 7 |
| *Clytia hemisphaerica* (Cnidaria, Hydrozoa) | *piwi* | nematoblasts | tentacle bulb | throughout ontogeny | ISH | 8, 5 |
| *Hydractinia echinata* (Cnidaria, Hydrozoa) | *piwi* | i-cells | stolon and polyp | throughout ontogeny | ISH | 9 |
| *Pleurobrachia pileus* (Ctenophora) | *PpiPiwi1* | progenitors of colloblasts  muscle cell progenitors  cells of the forming combs  cells of the aboral sense organ | tentacle roots  tentacle roots  comb rows  aboral sense organ | throughout ontogeny | ISH | 10 |
| *Isodiametra pulchra* (Acoelomorpha) | *Ipiwi* | neoblasts | whole animals | throughout ontogeny | ISH | 11 |
| *Macrostomum lignano* (Platyhelminthes, Rhabditophora) | *Macpiwi-1* | neoblasts | whole animals | throughout ontogeny | ISH | 12 |
|  | *Macpiwi-2* | neoblasts  γ-radiation-resistant cells | whole animals  regeneration blastema | throughout ontogeny  during regeneration | ISH | 12, 13 |
| *Schmidtea mediterranea* (Platyhelminthes, Rhabditophora) | *Smedwi-1* | neoblasts | whole animals | throughout ontogeny | ISH, FACS | 14 |
|  | *Smedwi-2* | neoblasts | whole animals | throughout ontogeny | ISH, FACS, | 14, 15 |
|  | *Smedwi-3* | neoblasts | whole animals | throughout ontogeny | ISH, FACS | 15 |
| *Dugesia japonica* (Platyhelminthes, Rhabditophora) | *DjPiwi-1* | neoblasts of the dorsal parenchyma | whole animals | throughout ontogeny | ISH, RNAseq | 16, 17 |
|  | *DjPiwi-2*  *DjPiwi-3* | neoblasts | whole animals | throughout ontogeny | ISH, RNAseq | 17 |
|  | *DjPiwi-A (Djpiwi-4)* | neoblasts and central nervous system (CNS) (cytoplasm) | whole animals | throughout ontogeny | ISH | 18, 19 |
|  | *DjPiwi-B* | neoblasts and their descendants (nucleus) | whole animals | throughout ontogeny | ICC, ISH | 19 |
|  | *DjPiwi-C* | neoblasts (cytoplasm) | whole animals | throughout ontogeny | ICC, ISH | 19 |
|  | *Djago-2* | neoblasts, brain, intestine | whole animals | throughout ontogeny | ISH | 18 |
| *Schistosoma mansoni* (Platyhelminthes, Neodermata, Trematoda) | *Smago2-1* | neoblasts | whole animals | throughout ontogeny | ISH, iRNA | 20 |
| *Platynereis dumerilii* (Annelida, Polychaeta) | *Pdu-piwiA*  *Pdu-piwiB* | proliferating, undifferentiated cells of the growth zone and blastema | posterior growth zone  blastema | metamorphosing larva  throughout ontogeny (posterior elongation)  regeneration  metamorphosing larva | ICC, ISH | 21,  22, 23 |
| *Alitta virens* (Annelida, Polychaeta) | *Avi-Piwil1* | proliferating, undifferentiated cells of the growth zone and blastema | posterior growth zone  blastema | metamorphosing larva  throughout ontogeny (posterior elongation)  regeneration | ISH | 24 |
|  | *Avi-Piwil2* | proliferating, undifferentiated cells of the blastema | blastema | regeneration | ISH | 24 |
| *Capitella teleta* (Annelida, Polychaeta) | *Ct-piwi1*  *Ct-piwi2* | proliferating, undifferentiated cells of the growth zone and blastema | posterior growth zone  blastema | metamorphosing larva  throughout ontogeny (posterior elongation)  regeneration | ISH | 25 |
| *Pristina leidyi* (Annelida, Oligochaeta) | *PRIle-piwi1* | proliferating, undifferentiated cells of the growth zone and fission zone  blastema | posterior growth zone  fission zone  blastema | metamorphosing larva  throughout ontogeny (posterior elongation) asexual reproduction  regeneration  metamorphosing larva | ISH | 26 |
| *Lymnaea stagnalis* (Mollusca, Gastropoda) | *Piwil1*  *Piwil2* | not reported | muscle  lung  brain | throughout ontogeny | qPCR | 27 |
| *Aplysia californica* (Mollusca, Gastropoda) | *piwi* | not reported | CNS, heart | throughout ontogeny | NB, ICC | 28 |
| *Crassostrea gigas* (Mollusca, Bivalvia) | *Piwil1*  *Piwil2* | not reported | labial palps  gills  adductor muscle  mantle | throughout ontogeny | qPCR | 27 |
| *Lytechinus variegatus* (Echinodermata, Echinoidea) | *Piwi* | tube feet epithelium  epithelium of the spines | tube feet  spines | throughout ontogeny | ICC, qPCR | 29 |
| *Botryllus schlosseri* (Chordata, Tunicata) | *Bs-piwi* | phagocytes near the endostyle (Ab)  tunic cells (Ab)  stomach cells (Ab) | haemolymph, tunic | throughout ontogeny and astogeny | ISH, IHC; iRNA | 30 |
| *Botrylloides leachii* (Chordata, Tunicata) | *Bl-piwi* | activated dormant cells lining the vasculature epithelium  cell islands | colonial vasculature | during whole body regeneration (WBR) | IHC; iRNA | 31 |
| *Botrylloides violaceus* (Chordata, Tunicata) | *piwi* | endostyle, haemocytes  haemocytes, few epithelial cells | filtering zooids    regenerating buds | throughout ontogeny    buds from stage 3–6 | IHC with commercial Abs | 32 |
| *Botrylloides diegensis* (Chordata, Tunicata) | *piwi2* | haemoblast | colonial vasculature | throughout ontogeny | ISH | 33 |
| *Ciona intestinalis* (Chordata, Tunicata) | *piwi-like (1,2)* | stem cells | gut epithelium  vessels of the branchial sac  basal stalk  cell clusters in the siphon walls  lymph nodes in pharynx, endostyle, atrial epithelium | continuous turnover of cells  forming body growth zone | IHC with commercial Abs | 34 |
| *Styela plicata* (Chordata, Tunicata) | *piwi* | haemoblasts | intestine submucosa | adults | IHC with commercial Abs | 35 |
| ***DEAD and DEAH-box-containing helicases*** | | | | | | |
| *Ephydatia fluviatilis* (Porifera, Demospongiae) | *Ef-vasa*  *Ef-pl10*  *Ef-ddx6* | archeocytes | mesohyl | throughout ontogeny | ISH, scRNAseq | 2 |
| *Sycon ciliatum* (Porifera, Calcarea) | *Sci-vasaB*  *Sci-pl10B* | choanocytes | choanocyte chambers | throughout ontogeny | ISH | 36 |
| *Oscarella lobularis* (Porifera, Homoscleromorpha) | *Ol-vasa*  *Ol-pl10* | choanocytes  pinacocytes  vacuolar cells type 1 | choanocyte chambers  pinacoderm  mesohyl | throughout ontogeny  during wound healing  throughout ontogeny | ISH | 3 |
| *Hydra magnipapillata* (Cnidaria, Hydrozoa) | *Cn-vas1*  *Cn-vas2* | i-cells (+)  epidermal cells | whole animals  body column | throughout ontogeny | ISH | 37 |
|  | *Cn-pl10* | i-cells  nematoblasts  epidermal cells | whole animals  whole animals  body column | throughout ontogeny | ISH | 37 |
| *Hydractinia echinata* (Cnidaria, Hydrozoa) | *vasa* | i-cells | epidermis of stolon and polyp | throughout ontogeny | ISH | 9, 5 |
| *Podocoryne carnea* (Cnidaria, Hydrozoa) | *vasa* | nematoblasts | tentacle bulbs and manubrium | medusa | ISH | 5 |
| *Nanomia bijuga* (Cnidaria, Hydrozoa, Siphonophora) | *vasa-1*  *pl10* | i-cells (epiderm and gastroderm) | siphonosomal horn  buds  young zooids | throughout ontogeny | ISH | 7 |
| *Pleurobrachia pileus* (Ctenophora) | *Ppi-vasa*  *Ppi pl10* | progenitors of colloblasts  muscle cell progenitors  cells of the forming combs  cells of the aboral sense organ | tentacle roots  tentacle roots  comb rows  aboral sense organ | whole adult animals | ISH | 10 |
| *Macrostomum lignano* (Platyhelminthes, Rhabditophora) | *Mac-vasa* | neoblasts (Ab + ISH)) | body parenchyma | whole animals | ICC, ISH | 38 |
| *Dugesia japonica* (Platyhelminthes, Rhabditophora) | *Dj-vas-1* | neoblasts | body parenchyma | whole animals | ISH | 18, 39 |
|  | *Dj-vlgA* | neoblasts and CNS | body parenchyma | regenerating animals | ISH, iRNA | 18, 39, 39 |
|  | *Dj-vlgB* | neoblasts | body parenchyma | whole animals | ISH, RNAseq | 17, 40 |
|  | *Dj-tud-1* | neoblasts | body parenchyma | ontogeny | ISH | 18 |
|  | *Dj-dhx-8c* | neoblasts and CNS | body parenchyma and brain | whole animals | ISH | 18 |
|  | *Dj-cbc-1 (ddx6)* | neoblasts and CNS | body parenchyma and brain | throughout ontogeny | ISH | 6, 18 |
| *Schmidtea mediterranea* (Platyhelminthes, Rhabditophora) | *Smed-vasa-1* | neoblasts | body parenchyma | adult animals | ICC, ISH, iRNA | 39, 38 |
|  | *Smed-vasa-2* | neoblasts | body parenchyma | adult animals | ISH, iRNA, NB | 39, 40 |
|  | *Smed-tud1A*  *Smed-tud1B* | neoblasts | body parenchyma | throughout ontogeny | ISH | 41 |
|  | *Smed-tdrd1L2* | neoblasts | body parenchyma | throughout ontogeny | ISH, scqPCR, cell transplantation | 42 |
| *Platynereis dumerilii* (Annelida, Polychaeta) | *Pdu-vasa*  *Pdu-pl10* | proliferating, undifferentiated cells of the growth zone and blastema | posterior growth zone  blastema | metamorphosing larva  throughout ontogeny (posterior elongation)  regeneration | ICC, ISH | 21–23 |
| *Alitta virens* (Annelida, Polychaeta) | *Avi-vasa*  *Avi-pl10* | proliferating, undifferentiated cells of the growth zone and blastema | posterior growth zone  blastema | metamorphosing larva  throughout ontogeny (posterior elongation)  regeneration | ISH | 24 |
| *Capitella teleta* (Annelida, Polychaeta) | *CapI-vasa* | proliferating, undifferentiated cells of the growth zone and blastema | posterior growth zone  blastema | metamorphosing larva  throughout ontogeny (posterior elongation)  regeneration  metamorphosing larva | ISH | 43 |
| *Pristina leidyi* (Annelida, Oligochaeta) | *Prile-vasa* | proliferating, undifferentiated cells of the growth zone and fission zone  blastema | posterior growth zone  fission zone | metamorphosing larva  throughout ontogeny (posterior elongation) asexual reproduction  regeneration  metamorphosing larva | ISH | 26 |
| *Enchytraeus japonensis* (Annelida, Oligochaeta) | *Ej-vlg2* | neoblasts and N-cells (only for mesoderm) | posterior surface of septa (N-cells dorsal to neoblasts) | during asexual reproduction by autotomy throughout | ISH | 44 |
| *Polyascus polygenea* (Cirripedia, Rhizocephala) | *Pp-vlg* | stem cells | stolons and buds of the asexual organism | asexual reproduction | ISH | 45 |
|  | *Pp-drh1* | stem cells | stolons and buds of *interna* | *interna* asexual reproduction | ISH | 45 |
| *Eriocheris sinensis* (Crustacea, Decapoda) | *ddx6* | cells of various tissues | heart, stomach muscle, haemocytes, thorax, intestine | throughout ontogeny | IHC with commercial Abs | 46 |
| *Lytechinus variegatus* (Echinodermata) | *vasa* | tube feet epithelium  epithelium of the spines  oesophagus epithelium  neurons  coelomocytes | tube feet (RNA and Ab)  spines (RNA and Ab)  oesophagus (Ab)  radial nerve (Ab)  coelom | throughout ontogeny | ICC, qPCR | 29 |
| *Botryllus schlosseri* (Chordata, Tunicata) | *Bs-vasa* | epithelial cells  phagocytes in cell islands (Ab)  stomach cells (Ab) | bud tissues  haemolymph  tissues of filtering zooids | throughout astogeny  throughout ontogeny and astogeny | ISH, IHC | 30 |
|  | *Bs-pl10* | epithelial cells  some blood cells,  phagocytes in cell islands (Ab)  stomach cells (Ab) | bud tissues  haemolymph  tissues of filtering zooids | throughout astogeny  throughout ontogeny and astogeny | ISH, IHC, ICC | 30, 47 |
|  | *Bs-ddx1* | cell islands | haemolymph | throughout ontogeny and astogeny | IHC, ISH | 48 |
| *Botrylloides violaceus* (Chordata, Tunicata) | *Bv-vasa* | haemocytes in vasculature (3–6%)  cells outside the vessels in proximity of the tunic | haemolymph | throughout ontogeny and astogeny | ISH | 49 |
| *Botrylloides diegensis* (Chordata, Tunicata) | *vasa* | haemoblasts | colonial vasculature | throughout ontogeny | ISH | 33 |
| ***tudor* domain-containing proteins** | | | | | | |
| *Ephydatia fluviatilis* (Porifera, Demospongiae) | *Ef-tudor9*  *Ef-tdrKH*  *Ef-tdrd1*  *Ef-tdrd5* | archeocytes | mesohyl | throughout ontogeny | ISH, scRNAseq | 2 |
| *Oscarella lobularis* (Porifera, Homoscleromorpha) | *Ol-tudor1* | choanocytes  pinacocytes  vacuolar cells type 1 | choanocyte chambers  pinacoderm  mesohyl | throughout ontogeny | ISH | 3 |
| *Hydra magnipapillata* (Cnidaria, Hydrozoa) | *Hm-tdrD9* | i-cells | body column | throughout ontogeny | ICC | 50 |
| *Hydra vulgaris* (Cnidaria, Hydrozoa) | *Hv-tdrd5* | i-cells | body column | throughout ontogeny | ISH, scRNAseq | 2 |
| *Schmidtea mediterranea* (Platyhelminthes, Rhabditophora) | *Sm-tdrd5* | neoblasts | whole animals | throughout ontogeny | ISH, scRNAseq | 2 |
| *Schmidtea polychroa* (Platyhelminthes, Rhabditophora) | *Spol-tud-1* | neoblasts  central nervous system | whole animals | throughout ontogeny | ISH | 41 |
| *Platynereis dumerilii* (Annelida, Polychaeta) | *Pdu-tdrd1*  *Pdu-tdrd2*  *Pdu-tdrd3* | proliferating, undifferentiated cells of the growth zone | posterior growth zone | metamorphosing larva  throughout ontogeny (posterior elongation) | ISH | 22 |
| ***PUF* family proteins** | | | | | | |
| *Oscarella lobularis* (Porifera, Homoscleromorpha) | *pumilio* | choanocytes  pinacocytes  vacuolar cells type 1 | choanocyte chambers  pinacoderm  mesohyl | throughout ontogeny  throughout ontogeny  throughout ontogeny | ISH | 3 |
| *Dugesia japonica* (Platyhelminthes, Rhabditophora) | *Dj-pum* | neoblasts | whole animals | throughout ontogeny | ISH | 18, 51 |
| *Schmidtea mediterranea* (Platyhelminthes, Rhabditophora) | *Smed-pumilio* | neoblasts | whole animals | throughout ontogeny | ISH | 41 |
| *Platynereis dumerilii* (Annelida, Polychaeta) | *Pdu-pumilio*  *Pdu-pufa*  *Pdu-pufb* | proliferating, undifferentiated cells of the growth zone | posterior growth zone | metamorphosing larva  throughout ontogeny (posterior elongation) | ISH | 22 |
| ***nanos* family proteins** | | | | | | |
| *Oscarella lobularis* (Porifera, Homoscleromorpha) | *Ol-nanos* | choanocytes  pinacocytes  vacuolar cells type 1 | choanocyte chambers  pinacoderm  mesohyl | throughout ontogeny  throughout ontogeny  throughout ontogeny | ISH | 3 |
| *Hydra magnipapillata* (Cnidaria, Hydrozoa) | *Cn-nos1* | i-cells | whole animals | throughout ontogeny | ISH | 52 |
|  | *Cn-nos2* | endodermal epithelial cells | hypostome | throughout ontogeny | ISH, ICC | 52, 53 |
| *Hydractinia echinata* (Cnidaria, Hydrozoa) | *nanos2* | nematoblasts, maturing nematocytes  i-cells | whole animals | throughout ontogeny | ISH, ICC | 53 |
| *Nanomia bijuga* (Cnidaria, Hydrozoa, Siphonophora) | *nanos-1*  *nanos-2* | i-cells (epiderm and gastroderm) | siphonosomal horn  buds  young zooids | throughout ontogeny | ISH | 7 |
| *Dugesia japonica* (Platyhelminthes, Rhabditophora) | *Dj-nos* | neoblasts | asexual and sexual individuals  mesenchyme | throughout ontogeny | ISH | 54 |
| *Schmidtea mediterranea* (Platyhelminthes, Rhabditophora) | *Smed-nos* | eye precursor cells | body parenchyma | during regeneration | ISH | 55 |
| *Schistosoma mansoni* (Platyhelminthes, Neodermata, Trematoda) | *Sm-nanos-2* | neoblasts | body parenchyma | throughout ontogeny | ISH, iRNA | 20 |
| *Platynereis dumerilii* (Annelida, Polychaeta) | *Pdu-nanos* | proliferating, undifferentiated cells of the growth zone and blastema | posterior growth zone  blastema | metamorphosing larva  throughout ontogeny (posterior elongation)  regeneration | ICC, ISH | 21–23 |
| *Capitella teleta* (Annelida, Polychaeta) | *CapI-nanos* | proliferating, undifferentiated cells of the growth zone and blastema | posterior growth zone  blastema | metamorphosing larva  throughout ontogeny (posterior elongation)  regeneration  metamorphosing larva | ISH | 43 |
| *Botryllus primigenus* (Chordata, Tunicata) | *Bp-nos* | weak staining of pharyngeal epithelia of the developing budlets | pharyngeal epithelia | blastogenesis (budlets stages 1–6) | ISH, IHC | 56 |
| ***Mago nashi* family proteins** | | | | | | |
| *Ephydatia fluviatilis* (Porifera, Demospongiae) | *Ef-mago-nashi* | archeocytes | mesohyl | throughout ontogeny | ISH, scRNAseq | 2 |
| *Lubomirskia baicalensis* (Porifera, Demospongiae) | *mago-nashi* | ? | top of the branches | throughout ontogeny | ISH | 57 |
| **RNA recognition motif (RRM)-containing proteins** | | | | | | |
| *Ephydatia fluviatilis* (Porifera, Demospongiae) | *Ef-bruno* | archeocytes | mesohyl | throughout ontogeny | ISH, ICC | 2 |
|  | *Ef-lmsiA* | archeocytes | mesohyl | throughout ontogeny | ISH, scRNAseq | 58 |
| *Oscarella lobularis* (Porifera, Homoscleromorpha) | *Ol-boule*  *Ol-bruno*  *Ol-brunoB* | choanocytes  pinacocytes  vacuolar cells type 1 | choanocyte chambers  pinacoderm  mesohyl | throughout ontogeny  during wound healing  throughout ontogeny | ISH | 3 |
| *Nematostella vectensis* (Cnidaria, Anthozoa) | *Nv-msi* | neuronal progenitors | tentacle ectoderm | young polyp | ISH | 63 |
| *Pleurobrachia pileus* (Ctenophora) | *Ppi-bruno* | progenitors of colloblasts  muscle cell progenitors  cells of the forming combs  cells of the aboral sense organ | tentacle roots  tentacle roots  comb rows  aboral sense organ | whole animals | ISH | 10 |
| *Schmidtea mediterranea* (Platyhelminthes, Rhabditophora) | *Smed-bruli* | neoblasts, CNS | body parenchyma and brain | throughout ontogeny | ISH | 59, 60 |
|  | *Smed-khd-1*  *Smed-cip29* | neoblasts | body parenchyma | throughout ontogeny | ISH | 37 |
|  | *Smed-smB* | neoblasts | body parenchyma | throughout ontogeny | ISH, iRNA, FACS | 61 |
|  | *Smed-mbnl-1* | neoblasts  differentiated cells | body parenchyma | throughout ontogeny | ISH | 58 |
|  | *Smed-mbnl-like-1*  *Smed-mbnl-like-2*  *Smed-mbnl-like-3* | epidermis  gut tissues | whole animals | throughout ontogeny | ISH | 59 |
| *Dugesia japonica* (Platyhelminthes, Rhabditophora) | *Dj-tial-1*  *Dj-tial-2*  *Dj-tial-3*  *Dj-tial-4*  *D-jtial-5* | neoblasts | body parenchyma | throughout ontogeny | ISH | 18 |
|  | *Dj-bruli*  *Dj-pabpc-2*  *Dj-edc-4*  *Dj-cnot-6*  *Dj-gemin-5*  *Dj-dicer-1*  *Dj-lsm-14*  *Dj-sgm-7*  *Dj-rbm-18*  *Dj-fmrp-1* | neoblasts and CNS | body parenchyma and brain | throughout ontogeny | ISH | 18 |
|  | *Dj-xrn-1*  *Dj-g3bp*  *Dj-cnot-7*  *D-jdcp-1L*  *Dj-upf-1* | neoblasts, brain, intestine | whole animals | throughout ontogeny | ISH | 18 |
|  | *Dj-dmlg* | neoblasts  neural precursors and additional cell types | body parenchyma | throughout ontogeny | ISH, FACS, scqPCR | 62 |
| *Platynereis dumerilii* (Annelida, Polychaeta) | *Pdu-bruno*  *Pdu-smb* | proliferating, undifferentiated cells of the growth zone | posterior growth zone | metamorphosing larva  throughout ontogeny (posterior elongation) | ISH | 22 |
|  | *Pdu-musashi* | proliferating, undifferentiated cells of the growth zone  ventral nerve cord  brain | posterior growth zone | metamorphosing larva  throughout ontogeny (posterior elongation) | ISH | 22 |
| *Botryllus schlosseri* (Chordata, Tunicata) | *Bs-dazap1* | all tissues | buds | during blastogenesis | ISH | 64 |
| **Signal transduction pathways** | | | | | | |
| ***Wnt*** | | | | | | |
| *Sycon ciliatum* (Porifera, Calcarea) | *Sci-bra1* | choanocytes | choanocyte chambers | throughout ontogeny | ISH | 36 |
| *Hydra magnipapillata* (Cnidaria, Hydrozoa) | *wnt* | epidermis and gastrodermis | hypostome  apical end of buds  apical end of regenerating animals | throughout ontogeny  budding  during regeneration | ISH | 65 |
| *Hydractinia echinata* (Cnidaria, Hydrozoa) | *wnt3* | i-cells, nematoblasts | epidermis and gastrodermis | polyp | ISH | 66 |
| *Nematostella vectensis* (Cnidaria, Anthozoa) | *wnt* | epidermis and gastrodermis | apical end of the animal | throughout ontogeny | ISH | 67 |
| *Schmidtea mediterranea* (Platyhelminthes, Rhabditophora) | *Smed-junl-1*  *Smed-tcf15* | neoblasts | body parenchyma | throughout ontogeny | ISH | 39 |
| *Branchiostoma lanceolatum* (Chordata, Cephalochordata) | *wnt5* | cells of the blastema | regenerating tail | throughout ontogeny, regeneration | ISH | 68 |
| *Botryllus schlosseri* (Chordata, Tunicata) | *wnt2B* | all the tissues | secondary buds | stages 1–3 | ISH | 69 |
|  | *wnt5A* | mesenchymal cells | developing gonads | primary buds | ISH | 69 |
|  | *wnt9A* | all the tissues | secondary buds | emerging secondary buds | ISH | 69 |
| *Botrylloides diegensis* (Chordata, Tunicata) | *frizzled5/8*  *β-catenin*  *dishevelled* | cycling haemoblasts | colonial vasculature | during WBR | ISH | 33 |
| ***TGF-β/BMP*** | | | | | | |
| *Sycon ciliatum* (Porifera, Calcarea) | *smad1/5* | choanocytes (weak) | choanocyte chambers | throughout ontogeny | ISH | 36 |
|  | *smad4* | choanocytes (weak)  mesohyl cells (weak) | choanocyte chambers  mesohyl | throughout ontogeny  throughout ontogeny | ISH | 36 |
| *Schmidtea mediterranea* (Platyhelminthes, Rhabditophora) | *Smed-smad-6/7* | neoblasts | body parenchyma | throughout ontogeny | ISH, scqPCR, cell transplantation | 42 |
| *Branchiostoma lanceolatum* (Chordata, Cephalochordata) | *chordin* | cells of the regenerating notochord | regenerating tail | throughout ontogeny, regeneration | ISH | 68, 70, 71 |
| *Branchiostoma japonicum* (Chordata, Cephalochordata) | *bmp2/4* | cells around wound edge | regenerating tail | throughout ontogeny, regeneration | ISH, IHC, RNAseq | 71, 72 |
| *Botryllus schlosseri* (Chordata, Tunicata) | *Bs smad1/5/8* | phagocytes | haemocytes | throughout ontogeny | IHC, ISH | 48 |
| ***Notch*** | | | | | | |
| *Platynereis dumerilii* (Annelida, Polychaeta) | *Pdu-delta*  *Pdu-notch* | proliferating, undifferentiated cells of the growth zone | posterior growth zone | metamorphosing larva | ISH | 74 |
|  | *Pdu-hes4*  *Pdu-hes5*  *Pdu-hes6*  *Pdu-hes8* | proliferating, undifferentiated cells of the growth zone | posterior growth zone | metamorphosing larva  throughout ontogeny (posterior elongation) | ISH | 75 |
| *Botrylloides diegensis* (Chordata, Tunicata) | *notch1*  *notch2*  *hes1* | cycling haemoblasts | colonial vasculature | during WBR | ISH | 33 |
| ***Hedgehog*** | | | | | | |
| *Sycon ciliatum* (Porifera, Calcarea) | *Sci-gli* | choanocytes | choanocyte chambers | throughout ontogeny | IHC, iRNA | 31 |
| **Kinases** | | | | | | |
| *Schmidtea mediterranea* (Platyhelminthes, Rhabditophora) | *Smed-nlk-1*  *Smed-fgfr-1*  *Smed-fgfr-4* | neoblasts | body parenchyma | throughout ontogeny | ISH | 39 |
| *Schistosoma mansoni* (Platyhelminthes, Neodermata, Trematoda) | *Sm-fgfrA* | neoblasts | body parenchyma | throughout ontogeny | ISH, iRNA | 20 |
| *Polyandrocarpa misakiensis* (Chordata, Tunicata) | *Pm-rack1* | atrial epithelium  undifferentiated mesenchymal cells associated with epidermis  pharynx epithelium | developing buds      whole zooids | during dedifferentiation      throughout ontogeny | ISH, IHC | 76 |
| **Pair rule and segment polarity genes** | | | | | | |
| *Platynereis dumerilii* (Annelida, Polychaeta) | *Pdu-hunchback* | proliferating, undifferentiated cells of the growth zone and blastema | posterior growth zone | metamorphosing larva  throughout ontogeny (posterior elongation) | ISH | 22 |
|  | *Pdu-runt* | proliferating, undifferentiated cells of the growth zone and blastema | posterior growth zone  blastema | metamorphosing larva  throughout ontogeny (posterior elongation)  regeneration | ISH | 22, 23 |
| *Branchiostoma japonicum* (Chordata, Cephalochordata) | *runx* | distal cells of the regenerating oral cirrus | regenerating cirrus | throughout ontogeny, regeneration | ISH | 73 |
| **Transcription factors** | | | | | | |
| **Homeobox-containing proteins** | | | | | | |
| *Hydractinia echinata* (Cnidaria, Hydrozoa) | *pln (pou protein)* | i-cells | stolons | throughout ontogeny | ISH | 77 |
| *Isodiametra pulchra* (Acoelomorpha) | *Ip-pitx*  *Ip-six 1/2* | neoblasts  musculature | whole animals | throughout ontogeny | ISH | 78 |
| *Schmidtea mediterranea* (Platyhelminthes, Rhabditophora) | *Smed-prox-1* | neoblasts | body parenchyma | throughout ontogeny | ICC, ISH | 38 |
|  | *Smed-pbx-1*  *Smed-nkx2.2* | neoblasts | body parenchyma | throughout ontogeny | ISH | 49 |
|  | *Smed-pax3/7* | differentiating sensory neurons |  |  | ISH | 49 |
| *Platynereis dumerilii* (Annelida, Polychaeta) | *Pdu-cdx*  *Pdu-hox3* | proliferating, undifferentiated cells of the growth zone and blastema | posterior growth zone | metamorphosing larva  throughout ontogeny (posterior elongation) | ISH | 22 |
|  | *Pdu-evx* | proliferating, undifferentiated cells of the growth zone and blastema | posterior growth zone | metamorphosing larva  throughout ontogeny (posterior elongation) | ISH | 23 |
| *Branchiostoma lanceolatum* (Chordata, Cephalochordata) | *pax3/7* | cells of the blastema and regenerating nerve cord | tail regeneration | throughout ontogeny, regeneration | ISH, IHC | 68, 70 |
|  | *msx* | cells of the blastema | regenerating tail | throughout ontogeny, regeneration | ISH | 68, 70 |
| *Botryllus schlosseri* (Chordata, Tunicata) | *Bs-pitx* | cells of the peribranchial epithelium  inner wall of the oral siphon and tentacles; forming cerebral ganglion, developing gut | budlets (stage 1–3); left peribranchial chamber (stage 4–6)  zooids at stage 8–9 | during budding and blastogenesis | ISH, qPCR | 79, 80 |
|  | *Bs-oct-4* | epithelial cells (Ab) | branchial sac | throughout ontogeny and astogeny | ISH, IHC | 30 |
|  | *Bs-pou-3* | few cells of the proximal side of the bud | atrial epithelium | bud at stage 3 | ISH | 81 |
| *Botrylloides diegensis* (Chordata, Tunicata) | *pou3* | haemoblast | colonial vasculature | throughout ontogeny | ISH | 33 |
| ***Sox* family proteins** | | | | | | |
| *Clytia hemisphaerica* (Cnidaria, Hydrozoa) | *Che-sox1,*  *Che-sox3,*  *Che- sox10*  *Che-soc12* | i-cells | tentacle bulb | medusa | ISH | 82 |
| *Schmidtea mediterranea* (Platyhelminthes, Rhabditophora) | *Smed-soxB-1* | neoblasts | body parenchyma | throughout ontogeny | IHC, ISH | 48, 83 |
|  | *Smed-soxP-1* | neoblasts | body parenchyma | throughout ontogeny | ISH | 39 |
|  | *Smed-soxP-3* | neoblasts | body parenchyma | throughout ontogeny | ISH | 39 |
| *Branchiostoma lanceolatum* (Chordata, Cephalochordata) | *soxB2* | cells of the regenerating nerve cord | tail regeneration | throughout ontogeny,  regeneration | ISH | 68, 70 |
| *Branchiostoma japonicum* (Chordata, Cephalochordata) | *soxE* | distal cells of the regenerating oral cirrus | cirrus regeneration | throughout ontogeny, regeneration | ISH | 71, 73 |
| ***Fox* family proteins** | | | | | | |
| *Hydra vulgaris* (Cnidaria, Hydrozoa) | *foxO* | i-cells | polyp | polyp | ISH | 84 |
| *Isodiametra pulchra* (Acoelomorpha) | *Ip-fox A1*  *Ip-fox A2*  *Ip-fox C* | neoblasts  musculature | whole animals | throughout ontogeny | ISH | 78 |
| **Zinc finger proteins** | | | | | | |
| *Sycon ciliatum* (Porifera, Calcarea) | *Sci-gata* | choanocytes | choanocyte chambers | throughout ontogeny | ISH | 36 |
| *Hydra* (Cnidaria, Hydrozoa) | *Hy-myc1* | proliferating i-cells  nematoblast  gland cells | whole animals | polyp | ISH | 85, 86 |
|  | *Hy-myc2* | proliferating i-cells  epidermal cells  gastrodermal cells | whole animals | polyp | ISH | 85, 86 |
| *Hydractinia echinata* (Cnidaria, Hydrozoa) | *myc2* | i-cells | stolon | polyp | ISH | 5 |
| *Isodiametra pulchra* (Acoelomorpha) | *Ip-gata456* | neoblasts  musculature | whole animals | throughout ontogeny | ISH | 78 |
| *Schmidtea mediterranea* (Platyhelminthes, Rhabditophora) | *Smed-gata4/5/6* | neoblasts | body parenchyma | throughout ontogeny | IHC, ISH | 48 |
|  | *Smed-zfmym-1*  *Smed-zf207-1*  *Smed-fhl-1*  *Smed-zfp-1*  *Smed-egr-1* | neoblasts | body parenchyma | throughout ontogeny | ISH | 39 |
| *Platynereis dumerilii* (Annelida, Polychaeta) | *Pdu-myc* | proliferating, undifferentiated cells of the growth zone | posterior growth zone | metamorphosing larva  throughout ontogeny (posterior elongation) | ISH | 22 |
| *Holothuria glaberrima* (Echinodermata, Holothuroidea) | *myc* | coelomic epithelium, intestinal cells, neuroepithelial and glial cells | regenerating intestine and radial nerve cord | during regeneration | ISH | 87 |
| *Botryllus schlosseri* (Chordata, Tunicata) | *BsGATA4/5/6* | atrial epithelium of the bud | posterior side of the budlet | bud at stage 3 | ISH | 81 |
| *Botryllus primigenus* (Chordata, Tunicata) | *myc* | cells of the branchial epithelia  circulating haemocytes | growing palleal and vascular buds  haemocoel of developing zooids | strong signal during blastogenesis, in budlets (stages 1–6) and weak signal in the early primary buds. | ISH | 88, 89 |
| *Polyandrocarpa misakiensis* (Chordata, Tunicata) | *myc* | cells of the atrial epithelium and fibroblast-like cells involved in organogenesis | developing bud | more than one day before dedifferentiation | ISH | 90 |
| **Helix-Loop-Helix domain-containing proteins** | | | | | | |
| *Isodiametra pulchra* (Acoelomorpha) | *Ip-twist 1*  *Ip-twist 2* | neoblasts  musculature | whole animals | throughout ontogeny | ISH | 78 |
| **Initiation factors** | | | | | | |
| *Dugesia japonica* (Platyhelminthes, Rhabditophora) | *Dj-eif-2a*  *Dj-eif-3A*  *Dj-eif-4e*  *Dj-eif-4g*  *Dj-eif-5a*  *Dj-eif-4a3* | neoblasts | body parenchyma | throughout ontogeny | ISH | 18 |
| **T-box proteins** | | | | | | |
| *Sycon ciliatum* (Porifera, Calcarea) | *Sci-bra2* | choanocytes | choanocyte chambers | throughout ontogeny | ISH | 36 |
| **Other transcription factors** | | | | | | |
| *Dugesia japonica* (Platyhelminthes, Rhabditophora) | *Dj-ekf-2a* | neoblasts | body parenchyma | throughout ontogeny | ISH | 18 |
|  | *Dj-elk-3*  *D-jprohibitin-2*  *Dj-ctbp1* | neoblasts | body parenchyma | throughout ontogeny | ISH, RNAseq | 17 |
| *Platynereis dumerilii* (Annelida, Polychaeta) | *Pdu-id*  *Pdu-gcm* | proliferating, undifferentiated cells of the growth zone | posterior growth zone | metamorphosing larva  throughout ontogeny (posterior elongation) | ISH | 22 |
|  | *Pdu-ap2* | proliferating, undifferentiated cells of the growth zone and blastema | posterior growth zone  blastema | metamorphosing larva  throughout ontogeny (posterior elongation)  regeneration | ISH | 22, 23 |
| **Chromatin modification/cell cycle** | | | | | | |
| **Transcriptional silencers** | | | | | | |
| *Schmidtea mediterranea* (Platyhelminthes, Rhabditophora) | *Smed-bcl11a* | neoblasts | body parenchyma | throughout ontogeny | iRNA, FACS  ISH | 91, 92 |
|  | *Smed-sirt* | neoblasts | body parenchyma | throughout ontogeny | ISH | 75 |
|  | *Smed-cbx1* | neoblasts | body parenchyma | throughout ontogeny | ISH, FACS | 14, 93 |
| **Proteins involved in methylation–demethylation** | | | | | | |
| *Dugesia japonica* (Platyhelminthes, Rhabditophora) | *Dj-hrjda*  *Dj-hrjda* | neoblasts  differentiated cells | whole animals | throughout ontogeny | ISH | 94 |
| *Schmidtea mediterranea* (Platyhelminthes, Rhabditophora) | *Smed-sedt8* | neoblasts | body parenchyma | throughout ontogeny | ISH | 83 |
|  | *Sm-hrjda*  *Sm-hrjdb* | neoblasts  differentiated cells | whole animals | throughout ontogeny | ISH | 94 |
|  | *Smed-setd8-1* | neoblasts | body parenchyma | throughout ontogeny | ISH | 39, 95 |
|  | *Smed-nsd-1*  *Smed-mrg-1*  *Smed-rbbp4-1* | neoblasts | body parenchyma | throughout ontogeny | ISH | 39 |
|  | *Smed-brg1l* | neoblasts | body parenchyma | throughout ontogeny | ISH | 83 |
| **Proteins involved in acetylation–deacetylation** | | | | | | |
| *Dugesia japonica* (Platyhelminthes, Rhabditophora) | *Dj-taf-1-beta* | neoblasts | body parenchyma | throughout ontogeny | ISH, RNAseq | 17 |
|  | *dj-rbap48* | neoblasts | body parenchyma | throughout ontogeny | ISH, RNAseq | 17, 96 |
| *Schmidtea mediterranea* (Platyhelminthes, Rhabditophora) | *Smed-ash2l*  *Smed-prmt5* | neoblasts | body parenchyma | throughout ontogeny | ISH | 83 |
|  | *Smed-hdac1* | neoblasts | body parenchyma | throughout ontogeny | ISH, FACS | 14, 93 |
| **Histones** | | | | | | |
| *Dugesia japonica* (Platyhelminthes, Rhabditophora) | *Dj-h2az*  *Dj-rbp4*  *Dj-cip-29*  *Dj-hp1* | neoblasts | body parenchyma | throughout ontogeny | ISH, RNAseq | 17 |
| *Schmidtea mediterranea* (Platyhelminthes, Rhabditophora) | *Smed-xrn1*  *Smed-smarcc2*  *Smed-ssrp1* | neoblasts | body parenchyma | throughout ontogeny | ISH | 83 |
| *Branchiostoma lanceolatum* (Chordata, Cephalochordata) | *p-h3* | cells of the blastema and regenerating nerve cord and notochord (many pax3/7+ [paired box gene 3/7]) | tail regeneration | throughout ontogeny, regeneration | IHC | 70 |
| *Branchiostoma japonicum* (Chordata, Cephalochordata) | *p-h3* | isolated cells in the regenerating oral cirrus | oral cirrus regeneration | regeneration | IHC | 73 |
| *Botryllus schlosseri* (Chordata, Tunicata) | *p-h3* | budlet and primary buds  zooidal stomach | adults, buds | throughout ontogeny and astogeny | IHC with commercial Abs | 97 |
| *Botrylloides diegensis* (Chordata, Tunicata) | *p-h3* | haemoblasts | colonial vasculature | throughout ontogeny | ISH | 33 |
| *Styela plicata* (Chordata, Tunicata) | *p-h3* | haemoblasts | intestine submucosa | adults | putative ASC | 35 |
| ***Polycomb* group proteins** | | | | | | |
| *Schmidtea mediterranea* (Platyhelminthes, Rhabditophora) | *Smed-ezh* | neoblasts | body parenchyma | throughout ontogeny | ISH | 39 |
|  | *Smed-ezh2* | neoblasts | body parenchyma | throughout ontogeny | ISH, IHC | 76 |
|  | *Smed-sz12-1* | neoblasts | body parenchyma | throughout ontogeny | ISH | 39 |
|  | *Smed-eed-1* | neoblasts | body parenchyma | throughout ontogeny | ISH | 39, 83 |
|  | *Smed-bmi1*  *Smed-rnf2*  *Smed-suz12* | neoblasts | body parenchyma | throughout ontogeny | ISH | 83 |
| **Control of transcription** | | | | | | |
| *Schmidtea mediterranea* (Platyhelminthes, Rhabditophora) | *Smed-hcf1*  *Smed-leo1*  *Smed-ctr9* | neoblasts | body parenchyma | throughout ontogeny | ISH | 83 |
|  | *Smed-thoc4* | neoblasts | body parenchyma | throughout ontogeny | ISH | 93, 96 |
|  | *Smed-rrm2-1* | neoblasts | body parenchyma | throughout ontogeny | ISH | 93 |
|  | *Smed-chd4* | neoblasts | body parenchyma | throughout ontogeny | ISH, IHC | 98 |
| **Proliferation markers** | | | | | | |
| *Hymeniacidon perleve* (Porifera, Demospongiae) | *pcna* | archaeocytes | mesohyl | cultured cells | ICC with commercial Ab | 99 |
| *Ephydatia fluviatilis* (Porifera, Demospongiae) | *pcna*  *Ef-mcm2*  *Ef-ccnb1* | archaeocytes | mesohyl | throughout ontogeny | ISH, scRNAseq | 2 |
| *Hydra vulgaris* (Cnidaria, Hydrozoa) | *Hv-pcna*  *Hv-mcm2*  *Hv-ccnb1* | i-cells | body column | throughout ontogeny | ISH, scRNAseq | 2 |
| *Dugesia japonica* (Platyhelminthes, Rhabditophora) | *Dj-mcm2* | neoblasts  polymorphic large cells | whole animals  macerates of tissues excised just below wound | proliferating cells after X-ray irradiation  proliferating cells of intact and regenerating planaria 30–60 min after wound infliction | ISH, RNAseq | 17, 100 |
|  | *Dj-pcna* | neoblasts | body parenchyma | proliferating cells after X-ray irradiation | IHC | 101 |
|  | *Dj-kif-3a*  *Dj-kif-3b*  *Dj-kif-19b* | neoblasts and CNS | body parenchyma and brain | throughout ontogeny | ISH | 18 |
| *Schmidtea mediterranea* (Platyhelminthes, Rhabditophora) | *Smed-rb* | neoblasts | body parenchyma | throughout ontogeny | IHC, ISH | 48 |
|  | *Smed-pcna*  *Smed-mcm2* | neoblasts | body parenchyma | throughout ontogeny | ISH, scRNAseq | 2, 83 |
|  | *Smed-cyclinB* | neoblasts | body parenchyma | throughout ontogeny | ISH | 2, 93 |
|  | *Smed-p53* | neoblasts | body parenchyma | throughout ontogeny | IHC, ISH | 48 |
|  | *Smed-pp32a*  *Smed-prohibitin1* | neoblasts | body parenchyma | throughout ontogeny | ISH | 93 |
| *Macrostomum lignano* (Platyhelminthes, Rhabditophora) | *DUF2366/TIM29* | neoblasts | body parenchyma |  | ISH, iRNA, RNAseq | 102 |
| *Platynereis dumerilii* (Annelida, Polychaeta) | *Pdu-pcna* | proliferating, undifferentiated cells of the growth zone and blastema | posterior growth zone  blastema | metamorphosing larva  throughout ontogeny (posterior elongation)  regeneration | ISH | 22, 23 |
|  | *Pdu-cycb1*  *Pdu-cycb3* | proliferating, undifferentiated cells of the growth zone and blastema | posterior growth zone  blastema | metamorphosing larva  throughout ontogeny (posterior elongation)  regeneration | ISH | 23 |
| *Ptychodera flava* (Hemichordata, Enteropneusta) | *pcna* | cells of blastema (Ab) | regeneration blastema | throughout ontogeny | IHC with commercial Abs | 103 |
| *Polyandrocarpa misakiensis* (Chordata, Tunicata) | *Pm-pcna* | cells of the atrial epithelium  cells associated with epithelia | developing buds | during dedifferentiation | IHC | 104 |
| *Botrylloides violaceus* (Chordata, Tunicata) | *pcna* | haemocytes | regenerating buds, during WBR | buds from stage 4 | IHC with commercial Abs | 32 |
| *Botrylloides diegenesis* (Chordata, Tunicata) | *cyclin b* | haemoblasts | colonial vasculature | during WBR | ISH | 33 |
| **Cytostactic proteins** | | | | | | |
| *Polyandrocarpa misakiensis* (Chordata, Tunicata) | *tc14-1 (lectin)* | atrial epithelial cells  haemoblasts | bud | primordial bud stage  growing buds | IHC | 105 |
|  | *tc14-3 (lectin)* | haemocytes, atrial epithelium | adults, buds | throughout ontogeny and astogeny in haemocytes, only in growing buds for epithelium | IHC | 106 |
| **Regulators of mitochondrial dynamics** | | | | | | |
| *Dugesia japonica* (Platyhelminthes, Rhabditophora) | *Dj-sam68* | neoblasts | body parenchyma | throughout ontogeny | ISH, RNAseq | 17 |
| *Schmidtea mediterranea* (Platyhelminthes, Rhabditophora) | *Smed-armc1* | neoblasts | body parenchyma | throughout ontogeny | ISH | 39 |
| **Telomere protection** | | | | | | |
| *Hymeniacidon perleve* (Porifera, Demospongiae) | *telomerase* | archaeocytes | mesohyl | cultured cells | PCR | 99 |
|  | *telomerase reverse transcriptase* | archaeocytes | mesohyl | cultured cells | activity | 99 |
| *Ephydatia fluviatilis* (Porifera, Demospongiae) | *Ef-rtel1* | archaeocytes | mesohyl | throughout ontogeny | ISH, scRNAseq | 2 |
| *Hydra vulgaris* (Cnidaria, Hydrozoa) | *Hv-rtel1* | i-cells | body column | throughout ontogeny | ISH, scRNAseq | 2 |
| *Schmidtea mediterranea* (Platyhelminthes, Rhabditophora) | *Sm-rtel1* | neoblasts | body parenchyma | throughout ontogeny | ISH, scRNAseq | 2 |
|  | *Smed-tert* (telomerase reverse transcriptase) | neoblasts | body parenchyma | throughout ontogeny | ISH | 107 |
|  | *Smed-ob1* | all cells | whole body | throughout ontogeny | ISH | 108 |
| *Enchytraeus japonensis* (Anellida, Oligochaeta) | *telomerase* | neoblasts and N- cells (only for mesoderm) | posterior surface of the septa | during asexual reproduction by autotomy | ISH | 44 |
| *Botryllus schlosseri* (Chordata, Tunicata) | *pot1* | multipotent epithelia | budlets | throughout blastogenesis | ISH | 81 |
|  | *telomerase* | earliest asexual bud | budlets | throughout blastogenesis | ISH | 109 |
| **Other nucleic acid binding-proteins** | | | | | | |
| *Schmidtea mediterranea* (Platyhelminthes, Rhabditophora) | *Smed-pairbp-1*  *Smed-hmg-1*  *Smed-hmg-2* | neoblasts | body parenchyma | throughout ontogeny | ISH | 93 |
| **Markers of post-mitotic cells** | | | | | | |
| *Schmidtea mediterranea* (Platyhelminthes, Rhabditophora) | *Smed-prog-1*  *Smed-prog-2*  *Smed-porcn-a*  *Smed-mhc1* | neoblasts | body parenchyma | throughout ontogeny | ISH | 39, 42 |
| **Other genes** | | | | | | |
| *Schmidtea mediterranea* (Platyhelminthes, Rhabditophora) | *Smed-hnf4* | neoblasts | body parenchyma | throughout ontogeny | IHC, ISH | 48 |
| **Proteins involved in autophagy** | | | | | | |
| *Polyandrocarpa misakiensis* (Chordata, Tunicata) | *Pm-atg7* | atrial epithelium | developing buds | during dedifferentiation | ISH | 110 |
| **Control of differentiation** | | | | | | |
| *Dugesia japonica* (Platyhelminthes, Rhabditophora) | *Dj-jy1* | neoblasts, neurons | body parenchyma and brain | throughout ontogeny | ISH | 18 |
|  | *Dj-hsp60*  *Dj-ahnak* | neoblasts | body parenchyma | throughout ontogeny | ISH, RNAseq | 17 |
| *Schmidtea mediterranea* (Platyhelminthes, Rhabditophora) | *Smed-mhc1* | neoblasts | body parenchyma | throughout ontogeny | ISH | 43 |
|  | *cbp-2* | ubiquitous | body tissues | throughout ontogeny | ISH, iRNA | 111 |
| *Botrylloides leachii* (Chordata, Tunicata) | *Bl-raldh* | circulating phagocytes | haemolymph | throughout ontogeny and astogeny | ISH | 112 |
|  | *if-b* | atrial epithelium of buds | budlets | buds at stage 1–3 | ISH | 81 |
|  | *Bs-raldh* | inner epithelium of the bud | posterior side of the budlet | bud at stage 3 | ISH | 81 |
| ***myocyte enhancer factor-2* (Mef2)** | | | | | | |
| *Isodiametra pulchra* (Acoelomorpha) | *Ip-mef2* | neoblasts, musculature | whole animals | throughout ontogeny | ISH | 78 |
| **Niche interaction** | | | | | | |
| *Ephydatia fluviatilis* (Porifera) | *Ef-annexin* | choanocytes | choanocyte chambers | throughout ontogeny | ISH | 1 |
| *Schmidtea mediterranea* (Platyhelminthes, Rhabditophora) | *Smed-inx-13* | neoblasts | body parenchyma | throughout ontogeny | IHC, ISH | 48 |
| *Botryllus schlosseri* (Chordata, Tunicata) | *Bs cadherin* | aggregates of haemoblast  aggregates of phagocytes near the endostyle  bud epithelia | ampullae  cell islands  buds, cell islands | throughout ontogeny and astogeny    buds at stage 1–5 | ISH, IHC | 113 |
|  | *Bs-cd133* | budding ampullar epithelium  some haemocytes | circulation and vasculature | during vasculature regeneration | ISH, FACS | 114 |
| *Botrylloides diegensis* | *integrin alpha-6* | haemoblast | colonial vasculature | throughout ontogeny | ISH | 33 |
| **Others** | | | | | | |
| *Polyandrocarpa misakiensis* (Chordata, Tunicata) | *Pm-pumpA* | atrial epithelium | developing buds | during dedifferentiation | ISH | 110 |
| **miRNA** | | | | | | |
| *Schmidtea mediterranea* (Platyhelminthes, Rhabditophora) | *let-7a*  *mir-71b*  *mir-756*  *mir-13*  *mir-752* | neoblasts | body parenchyma | throughout ontogeny | differential expression after irradiation | 115, 116 |
|  | *let-7b*  *mir-2160* | neoblasts | body parenchyma | throughout ontogeny | differential expression after irradiation | 115 |
|  | *mir-36b*  *mir-2a*  *mir-2d* | neoblasts | body parenchyma | throughout ontogeny | differential expression after irradiation | 116 |

**Ab**, antibody; **FACS**, fluorescence-activated cell sorter; **IB**, immunoblot; **ICC**, immunocytochemistry; **IHC**, immunohistochemistry; **iRNA**, RNA interference; **ISH**, *in situ* hybridization hybridization; **NB**, northern blot; **qPCR**, quantitative PCR; **RNA seq**, RNA sequencing; **scRNAseq**, single cell RNA sequencing; **scqPCR**, single cell qPCR; WBR, whole body regeneration.

**References**

1. Funayama N, Nakatsukasa M, Mohri K, Masuda Y, Agata, K. 2010. Piwi expression in archeocytes and choanocytes in demosponges: Insights into the stem cell system in demosponges. Evol Dev. 12:275-287.

2. Alié A, Hayashi T, Sugimura I, Manuel M, Sugano W, Mano A, Satoh N, Agata K, Funayama N. 2015. The ancestral gene repertoire of animal stem cells. Proc Natl Acad Sci USA. 112(51):E7093-100.

3. Fierro-Constaín L, Schenkelaars Q, Gazave E, Haguenauer A, Rocher C, Ereskovsky A, Borchiellini C, Renard E. 2017. The conservation of the germline multipotency program, from sponges to vertebrates: a stepping stone to understanding the somatic and germline origins. Genome Biol Evol. 9:474-488.

4. Seipel K, Yanze N, Schmid V. 2004. The germ line and somatic stem cell gene Cniwi in the jellyfish *Podocoryne carnea*. Int J Dev Biol. 48:1-7.

5. Plickert G, Frank U, Müller WA. 2012. Hydractinia, a pioneering model for stem cell biology and reprogramming somatic cells to pluripotency. Int J Dev Biol. 56:519-534.

6. Juliano CE, Reich A, Liu N, Götzfried J, Zhong M, Uman S, Reenan RA, Wessel GM, Steele RE, Lin H. 2014. PIWI proteins and PIWI-interacting RNAs function in *Hydra* somatic stem cells. Proc Natl Acad Sci USA. 111:337-342.

7. Siebert S, Goetz FE, Church SH, Bhattacharyya P, Zapata F, Haddock SH, Dunn CW. 2015. Stem cells in *Nanomia bijuga* (Siphonophora), a colonial animal with localized growth zones. EvoDevo. 6:22.

8. Denker E, Manuel M, Leclere L, Le Guyader H, Rabet N. 2008. Ordered progression of nematogenesis from stem cells through differentiation stages in the tentacle bulb of *Clytia hemisphaerica* (Hydrozoa, Cnidaria). Dev Biol. 315:99–113.

9. Rebscher N, Volk C, Teo R, Plickert G. 2008. The germ plasm component Vasa allows tracing of the interstitial stem cells in the cnidarian *Hydractinia echinata*. Dev Dyn. 237:1736-1745.

10. Alié A, Leclère L, Jager M, Dayraud C, Chang P, Le Guyader H, Quéinnec E, Manuel M. 2011. Somatic stem cells express Piwi and Vasa genes in an adult ctenophore: ancient association of "germline genes" with stemness. Dev Biol. 350:183-197.

11. Egger B, Steinke D, Tarui H, De Mulder K, Arendt D, Borgonie G, Funayama N, Gschwentner R, Hartenstein V, Hobmayer B, Hooge M, Hrouda M, Ishida S, Kobayashi C, Kuales G, Nishimura O, Pfister D, Rieger R, Salvenmoser W, Smith J, Technau U, Tyler S, Agata K, Salzburger W, Ladurner P. 2009*b*. To be or not to be a flatworm: the acoel controversy. PLoS ONE. 4:e5502.

12. Pfister D, De Mulder K, Philipp I, Kuales G, Hrouda M, Eichberger P, Borgonie G, Hartenstein V, Ladurner P. 2007. The exceptional stem cell system of *Macrostomum* *lignano*: screening for gene expression and studying cell proliferation by hydroxyurea treatment and irradiation. Front Zool. 4:9.

13. Zhou X, Battistoni G, El Demerdash O, Gurtowski J, Wunderer J, Falciatori I Ladurner P, Schatz MC, Hannon GJ, Wasik KA. 2015. Dual functions of Macpiwi1 in transposon silencing and stem cell maintenance in the flatworm *Macrostomum lignano*. RNA. 21:1885-1897.

14. Reddien PW, Oviedo NJ, Jennings JR, Jenkin JC, Sánchez Alvarado A. 2005. SMEDWI-2 is a PIWI-like protein that regulates planarian stem cells. Science. 310:1327-1330.

15. Palakodeti D, Smielewska M, Lu YC, Yeo GW, Graveley BR. 2008. The PIWI proteins SMEDWI-2 and SMEDWI-3 are required for stem cell function and piRNA expression in planarians. RNA. 14:1174-1186.

16. Rossi L, Salvetti A, Lena A, Batistoni R, Deri P, Pugliesi C, Loreti E, Gremigni V. 2006. DjPiwi-1, a member of the PAZ-Piwi gene family, defines a subpopulation of planarian stem cells. Dev Genes Evol. 216:335-346.

17. Rossi L, Salvetti A, Marincola FM, Lena A, Deri P, Mannini L, Batistoni R, Wang E, Gremigni V. 2007. Deciphering the molecular machinery of stem cells: a look at the neoblast gene expression profile. Genome Biol. 8:R62.

18. Rouhana L, Shibata N, Nishimura O, Agata K. 2010. Different requirements for conserved post-transcriptional regulators in planarian regeneration and stem cell maintenance. Dev Biol. 341:429-443.

19. Shibata N, Kashima M, Ishiko T, Nishimura O, Rouhana L, Misaki K, Yonemura S, Saito K, Siomi H Siomi MC Agata K. 2016. Inheritance of a nuclear PIWI from pluripotent stem cells by somatic descendants ensures differentiation by silencing transposons in planarian. Dev Cell. 7:226-237.

20. Collins JJ 3rd, Wang B, Lambrus BG, Tharp ME, Iyer H, Newmark PA. 2013. Adult somatic stem cells in the human parasite *Schistosoma mansoni*. Nature. 494:476-479.

21. Rebscher N, Zelada-González F, Banisch TU, Raible F, Arendt D. 2007. Vasa unveils a common origin of germ cells and of somatic stem cells from the posterior growth zone in the polychaete *Platynereis dumerilii*. Dev Biol. 306:599-611.

22. Gazave E, Behague J, Laplane L, Guillou A, Demilly A, Balavoine G, Vervoort M. 2013. Posterior elongation in the annelid *Platynereis dumerilii* involves stem cells molecularly related to primordial germ cells. Dev Biol. 382:246-267.

23. Planques A, Malem J, Parapar J, Vervoort M, Gazave E. 2019. Morphological, cellular and molecular characterization of posterior regeneration in the marine annelid *Platynereis dumerilii*. Dev Biol. 445:189-210.

24 Kozin VV, Kostyuchenko RP, 2015. Vasa, pl10, and piwi gene expression during caudal regeneration of the polychaete annelid *Alitta virens*. Dev Genes Evol. 225:129-138.

25. Giani VC, Yamaguchi E, Boyle MJ, Seaver EC. 2011. Somatic and germline expression of piwi during development and regeneration in the marine polychaete annelid *Capitella teleta*. EvoDevo. 2:10.

26. Özpolat BD, Bely AE. 2015. Gonad establishment during asexual reproduction in the annelid *Pristina leidyi*. Dev Biol. 405:123-136.

27. Jehn J, Gebert D, Pipilescu F, Stern S, Kiefer JST, Hewel C, Rosenkranz D. 2018. PIWI genes and piRNAs are ubiquitously expressed in mollusks and show patterns of lineage-specific adaptation. Commun Biol. 1:137.

28. Rajasethupathy P, Antonov I, Sheridan R, Frey S, Sander C, Tuschl T, Kandel ER. 2012. A role for neuronal piRNAs in the epigenetic control of memory-related synaptic plasticity. Cell. 149:693-707.

29. Reinardy HC, Emerson CE, Manley JM, Bodnar AG. 2015. Tissue regeneration and biomineralization in sea urchins: role of Notch signaling and presence of stem cell markers. PLoS ONE. 10:e0133860.

30. Rosner A, Moiseeva E, Rinkevich Y, Lapidot Z, Rinkevich B. 2009. Vasa and the germ line lineage in a colonial urochordate. Dev Biol. 331:113-128.

31. Rinkevich Y, Rosner A, Rabinowitz C, Lapidot Z, Moiseeva E, Rinkevich B. 2010. Piwi positive cells that line the vasculature epithelium, underlie whole body regeneration in a basal chordate. Dev Biol. 345:94-104.

32. Brown FD, Keeling EL, Le AD, Swalla BJ. 2009. Whole body regeneration in a colonial ascidian, *Botrylloides violaceus*. J Exp Zool. 312B:885-900.

33. Kassmer SH, Langerbacher A, De Tomaso AW. (2020). Integrin-alpha-6+ candidate stem cells are responsible for whole body regeneration in the invertebrate chordate *Botrylloides diegensis*. Nat Commun. 11:4435.

34. Jeffery, WR. 2015*a*. Distal regeneration involves the age dependent activity of branchial sac stem cells in the ascidian *Ciona intestinalis*. Regeneration. 2:1-18.

35. Jiménez-Merino J, Santos de Abreu I, Hiebert LS, Allodi S, Tiozzo S, De Barros C, Brown FD. 2019. Putative stem cells in the hemolymph and the intestinal submucosa of the solitary ascidian *Styela plicata*. EvoDevo. 10:31.

36. Leininger S, Adamski M, Bergum B, Guder C, Liu J, Laplante M, Bråte J, Hoffmann F, Fortunato S, Jordal S, Rapp HT, Adamska M. 2014. Developmental gene expression provides clues to relationships between sponge and eumetazoan body plans. Nat Commun. 5:3905.

37. Mochizuki K, Nishimiya-Fujisawa C, Fujisawa T. 2001. Universal occurrence of the vasa-related genes among metazoans and their germline expression in *Hydra*. Dev Genes Evol. 211:299-308.

38. Pfister D, De Mulder K, Hartenstein V, Kuales G, Borgonie G, Marx F, Morris J, Ladurner P. 2008. Flatworm stem cells and the germ line: developmental and evolutionary implications of macvasa expression in *Macrostomum lignano*. Dev Biol. 319:146-159.

39. Wagner DE, Ho JJ, Reddien PW. 2012. Genetic regulators of a pluripotent adult stem cell system in planarians identified by RNAi and clonal analysis. Cell Stem Cell. 10:299-311.

40. Shibata N, Umesono Y, Orii H, Sakurai T, Watanabe K, Agata K. 1999. Expression of vasa(vas)-related genes in germline cells and totipotent somatic stem cells of planarians. Dev Biol. 206:73-87.

41. Solana J, Lasko P, Romero R. 2009. Spoltud-1 is a chromatoid body component required for planarian long-term stem cell self-renewal. Dev Biol. 328:410-421.

42. Van Wolfswinkel JC, Wagner DE, Reddien PW. 2014. Single-cell analysis reveals functionally distinct classes within the planarian stem cell compartment. Cell Stem Cell. 15:326-339.

43. Dill KK, Seaver EC. 2008. Vasa and nanos are coexpressed in somatic and germ line tissue from early embryonic cleavage stages through adulthood in the polychaete *Capitella* sp. I. Dev Genes Evol. 218:453-463.

44. Sugio M, Yoshida-Noro C, Ozawa K, Tochinai S. 2012. Stem cells in asexual reproduction of *Enchytraeus japonensis* (Oligochaeta, Annelida): proliferation and migration of neoblasts. Dev Growth Differ. 54:439-450.

45. Shukalyuk AI, Golovnina KA, Baiborodin SI, Gunbin KV, Blinov AG, Isaeva VV. 2007. vasa-related genes and their expression in stem cells of colonial parasitic rhizocephalan barnacle *Polyascus polygenea* (Arthropoda: Crustacea: Cirripedia: Rhizocephala). Cell Biol Int. 31:97-108.

46. Li Q, Wang YL, Xie J, Sun WJ, Zhu M, He L, Wang Q. 2015. Characterization and expression of DDX6 during gametogenesis in the Chinese mitten crab *Eriocheir* *sinensis*. Genet Mol Res. 14:4420-4437.

47. Rosner A, Paz G, Rinkevich B. 2006. Divergent roles of the DEAD-box protein BS-PL10, the urochordate homologue of human DDX3 and DDX3Y proteins, in colony astogeny and ontogeny. Dev Dyn. 235:1508-1521.

48. Rosner A, Moiseeva E, Rabinowitz C, Rinkevich B. 2013. Germ lineage properties in the urochordate *Botryllus schlosseri* - from markers to temporal niches. Dev Biol. 384:356-374.

49. Brown FD, Swalla BJ. 2007. Vasa expression in a colonial ascidian, *Botrylloides violaceus*. Evol Dev. 9:165-177.

50. Lim RS, Anand A, Nishimiya-Fujisawa C, Kobayashi S, Kai T. 2014. Analysis of Hydra PIWI proteins and piRNAs uncover early evolutionary origins of the piRNA pathway. Dev Biol. 386:237-251.

51. Salvetti A, Rossi L, Lena A, Batistoni R, Deri P, Rainaldi G, Locci MT, Evangelista M, Gremigni V. 2005. DjPum, a homologue of *Drosophila* Pumilio, is essential to planarian stem cell maintenance. Development. 132:1863-1874.

52. Mochizuki K, Sano H, Kobayashi S, Nishimiya-Fujisawa C, Fujisawa T. 2000. Expression and evolutionary conservation of nanos-related genes in *Hydra*. Dev Genes Evol. 210:591-602.

53. Kanska J, Frank U. 2013. New roles for Nanos in neural cell fate determination revealed by studies in a cnidarian. J Cell Sci. 126:3192-3203.

54. Sato K, Shibata N, Orii H, Amikura R, Sakurai T, Agata K, Kobayashi S, Watanabe K. 2006. Identification and origin of the germline stem cells as revealed by the expression of nanos-related gene in planarians. Dev Growth Differ. 48:615-628.

55. Handberg-Thorsager M, Saló E. 2007. The planarian nanos-like gene Smednos is expressed in germline and eye precursor cells during development and regeneration. Dev Genes Evol. 217:403-411.

56. Sunanaga T, Satoh M, Kawamura K. 2008. The role of Nanos homologue in gametogenesis and blastogenesis with special reference to male germ cell formation in the colonial ascidian, *Botryllus primigenus*. Dev Biol. 324:31-40.

57. Wiens M, Belikov SI, Kaluzhnaya OV, Krasko A, Schröder HC, Perovic-Ottstadt S, Müller WE. 2006. Molecular control of serial module formation along the apical–basal axis in the sponge *Lubomirskia baicalensis*: silicateins, mannose-binding lectin and mago nashi. Dev Genes Evol. 216:229-242.

58. Okamoto K, Nakatsukasa M, Alié A, Masuda Y, Agata K, Funayama N. 2012. The active stem cell specific expression of sponge Musashi homolog EflMsiA suggests its involvement in maintaining the stem cell state. Mech Dev. 129:24-37,

59. Solana J, Irimia M, Ayoub S, Rodriguez Orejuela M, Zywitza V, Jens M, Tapial Jr, Ray D, Morris Q, Hughes TR, Blencowe BJ, Rajewsky N. 2016. Conserved functional antagonism of CELF and MBNL proteins controls stem cell-specific alternative splicing in planarians. eLife. 5:e16797.

60. Guo T, Peters AH, Newmark PA. 2006. A Bruno-like gene is required for stem cell maintenance in planarians. Dev Cell. 11:159-169.

61. Fernandéz-Taboada E1, Moritz S, Zeuschner D, Stehling M, Schöler HR, Saló E, Gentile L. 2010. Smed-SmB, a member of the LSm protein superfamily, is essential for chromatoid body organization and planarian stem cell proliferation. Development. 137:1055-1065.

62. Higuchi S, Hayashi T, Tarui H, Nishimura O, Nishimura K, Shibata N, Sakamoto H, Agata K. 2008. Expression and functional analysis of musashi-like genes in planarian CNS regeneration. Mech Dev. 125:631-645.

63. Marlow HQ, Srivastava M, Matus DQ, Rokhsar D, Martindale MQ. 2009. Anatomy and development of the nervous system of *Nematostella vectensis*, an anthozoan cnidarian. Dev Neurobiol. 69:235-254.

64. Gasparini F, Shimeld SM, Ruffoni E, Burighel P, Manni L. 2011. Expression of a Musashi-like gene in sexual and asexual development of the colonial chordate *Botryllus schlosseri* and phylogenetic analysis of the protein group. J Exp Zool. 316B:562-573.

65. Lengfeld T, Watanabe H, Simakov O, Lindgens D, Gee L, Law L, Schmidt HA, Ozbek S, Bode H, Holstein TW. 2009. Multiple Wnts are involved in *Hydra* organizer formation and regeneration. Dev Biol. 330:186-199.

66. Müller W, Frank U, Teo R, Mokady O, Guette C, Plickert G. 2007. Wnt signaling in hydroid development: ectopic heads and giant buds induced by GSK-3beta inhibitors. Int J Dev Biol. 51:211-220.

67. Kusserow A, Pang K, Sturm C, Hrouda M, Lentfer J, Schmidt HA, Technau U, von Haeseler A, Hobmayer B, Martindale MQ, Holstein TW. 2005. Unexpected complexity of the Wnt gene family in a sea anemone. Nature. 433:156-160.

68. Somorjai IML. 2017. Amphioxus regeneration: evolutionary and biomedical implications. Int J Dev Biol. 61:689-696.

69. Di Maio A, Setar L, Tiozzo S, De Tomaso AW. 2015. Wnt affects symmetry and morphogenesis during post-embryonic development in colonial chordates. EvoDevo. 6:17.

70. Somorjai IM, Somorjai RL, Garcia-Fernàndez J, Escrivà H. 2012*b*. Vertebrate-like regeneration in the invertebrate chordate amphioxus. Proc Natl Acad Sci USA. 109:517-522.

71. Ferrario C, Sugni M, Somorjai IML, Ballarin L. 2020. Beyond adult stem cells: dedifferentiation as a unifying mechanism underlying regeneration in invertebrate deuterostomes. Front Cell Dev Biol. 8:587320.

72. Liang Y, Rathnayake D, Huang S, Pathirana A, Xu Q, Zhang S. 2019. BMP signaling is required for amphioxus tail regeneration. Development. 146(4):dev166017.

73. Kaneto S, Wada H. 2011. Regeneration of amphioxus oral cirri and its skeletal rods: implications for the origin of the vertebrate skeleton. J Exp Zool. 316B:409-17.

74. Gazave E, Lemaître Q, Balavoine G. 2017. The Notch pathway in the annelid *Platynereis*: insights into chaetogenesis and neurogenesis processes. Open Biol. 7:160242.

75. Gazave E, Guillou A, Balavoine G. 2014. History of a prolific family: the Hes/Hey-related genes of the annelid *Platynereis*. EvoDevo. 5:29.

76. Tatzuke Y, Sunanaga T, Fujiwara S, Kawamura K. 2012. RACK1 regulates mesenchymal cell recruitment during sexual and asexual reproduction of budding tunicates. Dev Biol. 368:393-403.

77. Millane RC, Kanska J, Duffy DJ, Seoighe C, Cunningham S, Plickert G, Frank U. 2011. Induced stem cell neoplasia in a cnidarian by ectopic expression of a POU domain transcription factor. Development. 138:2429-2439.

78. Chiodin M, Børve A, Berezikov E, Ladurner P, Martinez P, Hejnol A. 2013. Mesodermal gene expression in the acoel *Isodiametra pulchra* indicates a low number of mesodermal cell types and the endomesodermal origin of the gonads. PLoS ONE. 8.e55499.

79. Tiozzo S, Christiaen L, Deyts C, Manni L, Joly JS, Burighel P. 2005. Embryonic versus blastogenetic development in the compound ascidian *Botryllus schlosseri*: insights from Pitx expression patterns. Dev Dyn. 232:468-478.

80. Tiozzo S, De Tomaso AW. 2009. Functional analysis of Pitx during asexual regeneration in a basal chordate. Evol Dev. 11:152-162.

81. Ricci L, Chaurasia A, Lapébie P, Dru P, Helm RR, Copley RR, Tiozzo S. 2016. Identification of differentially expressed genes from multipotent epithelia at the onset of an asexual development. Sci Rep. 6:27357.

82. Jager M, Quéinnec E, Le Guyader H, Manuel M. 2011. Multiple Sox genes are expressed in stem cells or in differentiating neuro-sensory cells in the hydrozoan *Clytia* *hemisphaerica*. EvoDevo. 2:12.

83. Onal P, Grün D, Adamidi C, Rybak A, Solana J, Mastrobuoni G, Wang Y, Rahn HP, Chen W, Kempa S, Ziebold U, Rajewsky N. 2012. Gene expression of pluripotency determinants is conserved between mammalian and planarian stem cells. EMBO J. 31:2755-2769.

84. Boehm AM, Khalturin K, Anton-Erxleben F, Hemmrich G, Klostermeier UC, Lopez-Quintero JA, Oberg HH, Puchert M, Rosenstiel P, Wittlieb J, Bosch TC. 2012. FoxO is a critical regulator of stem cell maintenance in immortal *Hydra*. Proc Natl Acad Sci USA. 109:19697-19702.

85. Hartl M, Glasauer S, Valovka T, Breuker K, Hobmayer B, Bister K. 2014. *Hydra* myc2, a unique pre-bilaterian member of the myc gene family, is activated in cell proliferation and gametogenesis. Biol Open. 3:397-407.

86. Hobmayer B, Jenewein M, Eder D, Eder MK, Glasauer S, Gufler S, Hartl M, Salvenmoser W. 2012. Stemness in *Hydra* - A current perspective. Int J Dev Biol. 56:509-517.

87. Mashanov VS, Zueva OR, Garcia-Arrarás JE. 2015*a*. Expression of pluripotency factors in echinoderm regeneration. Cell Tissue Res. 359:521-536.

88. Kawamura K, Tachibana M, Sunanaga T. 2008*b*. Cell proliferation dynamics of somatic and germline tissues during zooidal life span in the colonial tunicate *Botryllus* *primigenus*. Dev Dyn. 237:1812-1825.

89. Kawamura K, Sunanaga T. 2011. Role of Vasa, Piwi, and Myc-expressing coelomic cells in gonad regeneration of the colonial tunicate, *Botryllus primigenus*. Mech Dev. 128:457-470.

90. Fujiwara S, Isozaki T, Mori K, Kawamura K. 2011. Expression and function of myc during asexual reproduction of the budding ascidian *Polyandrocarpa misakiensis*. Growth Differ. 53:1004-1014.

91. Resch AM, Palakodeti D, Lu YC, Horowitz M, Graveley BR. 2012. Transcriptome analysis reveals strain-specific and conserved stemness genes in *Schmidtea* *mediterranea*. PLoS ONE. 7:e34447.

92. Trost T, Haines J, Dillon A, Mersman B, Robbins M, Thomas P, Hubert A. 2018. Characterizing the role of SWI/SNF-related chromatin remodeling complexes in planarian regeneration and stem cell function. Stem Cell Res. 32:91-103.

93. Eisenhoffer GT, Kang H, Sánchez Alvarado A. 2008. Molecular analysis of stem cells and their descendents during cell turnover and regeneration in the planarian *Schmidtea mediterranea*. Cell Stem Cell. 3:327-339.

94. Cao PL, Kumagai N, Inoue T, Agata K, Makino T. 2019. JmjC domain-encoding genes are conserved in highly regenerative metazoans and are associated with planarian whole-body regeneration. Genome Biol Evol. 11:552-564.

95. Torre C, Abnave P, Tsoumtsa LL, Mottola G, Lepolard C, Trouplin V, Gimenez G, Desrousseaux J, Gempp S, Levasseur A, Padovani L, Lemichez E, Ghigo E. 2017. *Staphylococcus aureus* promotes Smed-PGRP-2/Smed-setd8-1 methyltransferase signalling in planarian neoblasts to sensitize anti-bacterial gene responses during re-infection. EBioMedicine. 20:150-160.

96. Bonuccelli L, Rossi L, Lena A, Scarcelli V, Rainaldi G, Evangelista M, Iacopetti P, Gremigni V, Salvetti A. 2010. An RbAp48-like gene regulates adult stem cells in planarians. J Cell Sci. 123:690-698.

97. Rosner A, Alfassi G, Moiseeva E, Paz G, Rabinowitz C, Lapidot Z, Douek J, Haim A, Rinkevich B. 2014. The involvement of three signal transduction pathways in botryllid ascidian astogeny, as revealed by expression patterns of representative genes. Int J Dev Biol. 58:677-692.

98. Scimone ML, Meisel J, Reddien PW. 2010. The Mi-2-like Smed-CHD4 gene is required for stem cell differentiation in the planarian *Schmidtea mediterranea*. Development. 137:1231-1241.

99. Sun L, Song Y, Qu Y, Yu X, Zhang W. 2007. Purification and *in vitro* cultivation of archaeocytes (stem cells) of the marine sponge *Hymeniacidon perleve* (Demospongiae). Cell Tissue Res. 328:223-237.

100. Salvetti A, Rossi L, Deri P, Batistoni R. 2000. An MCM2-related gene is expressed in proliferating cells of intact and regenerating planarians. Dev Dyn. 218:603-614.

101. Orii H, Sakurai T, Watanabe K. 2005. Distribution of the stem cells (neoblasts) in the planarian *Dugesia japonica*. Dev Genes Evol. 215:143-157.

102. Mouton S, Ustyantsev K, Beltman F, Glazenburg L, Berezikov E. 2021. Tim29 is required for stem cell activity during regeneration in the flatworm *Macrostomum* *lignano*. Sci Rep. 11:1166.

103. Rychel AL, Swalla BJ. 2008. Anterior regeneration in the hemichordate *Ptychodera flava*. Dev Dyn 237:3222-3232.

104. Kawamura K, Kitamura S, Sekida S, Tsuda M, Sunanaga T. 2012. Molecular anatomy of tunicate senescence: reversible function of mitochondrial and nuclear genes associated with budding cycles. Development 139:4083-4093.

105. Kawamura K, Fujiwara S, Sugino, YM. 1991. Budding-specific lectin induced in epithelial cells is an extracellular matrix component for stem cell aggregation in tunicates. Development 113:995-1005.

106. Matsumoto J, Nakamoto C, Fujiwara S, Yubisui T, Kawamura K. 2001. A novel C-type lectin regulating cell growth, cell adhesion and cell differentiation of the multipotent epithelium in budding tunicates. Development. 128:3339-3347.

107. Tan TC, Rahman R, Jaber-Hijazi F, Felix DA, Chen C, Louis EJ, Aboobaker A. 2012. Telomere maintenance and telomerase activity are differentially regulated in asexual and sexual worms. Proc Natl Acad Sci USA. 109:4209-4214.

108. Yin S, Huang Y, Zhangfang Y, Zhong X, Li P, Huang J, Liu D2, Songyang Z. 2016. SmedOB1 is required for planarian homeostasis and regeneration. Sci Rep. 6:34013.

109. Laird DJ, Weissman IL. 2004. Telomerase maintained in self-renewing tissues during serial regeneration of the urochordate *Botryllus schlosseri*. Dev Biol. 273:185-194.

110. Kawamura K, Yoshida T, Sekida S. 2018. Autophagic dedifferentiation induced by cooperation between TOR inhibitor and retinoic acid signals in budding tunicates. Dev Biol. 433:384-393.

111. Fraguas S, Cárcel1 S, Vivancos C, Molina MD, Ginés J, Mazariegos J, Sekaran T, Bartscherer K, Romero R, Cebrià F. 2021. Planarian CREB-binding protein (CBP) gene family regulates stem cell maintenance and differentiation. Dev Biol. 476:53-67.

112. Rinkevich Y, Paz G, Rinkevich B, Reshef R. 2007. Systemic bud induction and retinoic acid signaling underlie whole body regeneration in the urochordate *Botrylloides* *leachi*. PLoS Biol. 5:e71.

113. Rosner A, Rabinowitz C, Moiseeva E, Voskoboynik A, Rinkevich B. 2007. BS-cadherin in the colonial urochordate *Botryllus schlosseri*: one protein, many functions. Dev Biol. 304:687-700.

114. Braden BP, Taketa DA, Pierce JD, Kassmer S, Lewis DD, De Tomaso AW. 2014. Vascular regeneration in a basal chordate is due to the presence of immobile, bi-functional cells. PLoS ONE. 9:e95460.

115. Lu YC, Smielewska M, Palakodeti D, Lovci MT, Aigner S, Yeo GW, Graveley BR. 2009. Deep sequencing identifies new and regulated microRNAs in *Schmidtea* *mediterranea*. RNA. 15:1483-1491.

116. Friedländer MR, Adamidi C, Han T, Lebedeva S, Isenbarger TA, Hirst M, Marra M, Nusbaum C, Lee WL, Jenkin JC, Sánchez Alvarado A, Kim JK, Rajewsky N. 2009. High-resolution profiling and discovery of planarian small RNAs. Proc Natl Acad Sci USA. 106:11546-11551.
