# Supplementary material for: A pan‐metazoan concept for adult stem cells: the wobbling Penrose landscape
Source: Biol Rev Camb Philos Soc. 2021 Oct 6;97(1):299–325. doi: 10.1111/brv.12801 (PMC9292022; doi:10.1111/brv.12801)
Supplement: Supplementary file 5 — Table S3. Suggested stem cell niches (SCNs) present in invertebrates. [file BRV-97-299-s002.docx]

**Table S3.** Suggested stem cell niches (SCNs) present in invertebrates.

| **Phylum/species** | **Suggested niche** | **Evidence** | **Body location** | **Putative cellular components in niche** | **Gene activities associated with the niche** | **Reference** |
| --- | --- | --- | --- | --- | --- | --- |
| **PORIFERA** |  |  |  |  |  |  |
| *Asbestopluma hypogea* | Speculated presence of a niche in the peduncle. | Indirect evidence during the regeneration process in carnivorous species. Presence of high number of BrdU/EdU cells in the area (Martinand-Mari *et al*., 2012) | Peduncle, during post-digestion processes | No specific cell types described, besides some undefined migratory cells | No activities associated with the putative niche. Only Brdu/EdU activities reported in the area. Niches are ‘inferred’ | 1 |
| **CNIDARIA** |  |  |  |  |  |  |
| *Hydra*/*Hydractinia* (Hydrozoa) | Some evidence of presence of regulatory factors in the area surrounding interstitial cells (whole body) | Still mostly indirect. Putative involvement of the whole body. Complex environments surrounding the interstitial cells in ***Hydra*** described, suggesting active involvement in the maintenance of the stem cell state (David & Plotnick, 1980; Bode, 1996). Molecular data show the presence of regulatory factors (Wnts, peptides or the interaction with ECM) as necessary to maintain the proliferative/differentiation balance of the interstitial cells. ***Hydractinia*** may use a similar mechanism to regulate the stem cell state of interstitial cells (Gahan *et al*., 2016) | Interstitial cells are in contact with epithelial cells and the mesoglea  (Bosch, 2009) | Interstitial stem cells are always localized near, or even in physical contact with, both epithelial cells and the mesoglea. The whole body column is composed of continuously proliferating cells and may be considered a stem cell niche. Nerve cell density influences interstitial cell proliferation | Integrin and cadherin signalling (Bosch *et al*., 2010) from the matrix and Wnt and Notch pathway from the microenvironment. Takahashi *et al*. (2000) show that a peptide, Hym-355, expressed in surrounding (interstitial) cells directly affects interstitial cell differentiation by inhibiting commitment or migration of nerve precursor cells. Khalturin *et al.* (2007) show that the regulation of Wnt/beta-catenin signalling is important for maintaining the balance of proliferation *versus* differentiation in the interstitial cell system | 2–7, 32 |
| **CTENOPHORA** |  |  |  |  |  |  |
| *Pleurobrachia pileus* |  | Indirect evidence. stem cells are only located in very few areas of the adult. In adult *Pleurobrachia pileus* there are pools of stem cells associated with different structures (somatic stem cells). These cells are spatially restricted (Alié *et al*., 2011) | Cellular components of the putative niche are unknown |  |  | 8 |
| *Mnemiopsis leidyi* |  | No evidence in adults. indirect evidence in embryos. Cells surrounding the EdU-positive stem cells in *Mnemiopsis* (only in embryos) are thought to be working as functional niches (Presnell & Browne, 2019) | Putative stem cell niches identified within the aboral region of the pharynx and the median ridge of developing tentacle bulbs (but only in embryos) |  | MleKlf5a and MleKlf5b may play a role in the maintenance of the median ridge tentacular stem cell niche (knockdown experiments). Klfs promote cell proliferation within stem cell niches. The identity of the cells being regulated is not known (in embryogenesis) | 9 |
| **PLATYHELMINTHES** |  |  |  |  |  |  |
| *Schmidtea mediterranea* | Evidence of the involvement of intestinal cells in regulating proliferation/differentiation. The whole gut may provide a niche for neoblasts. Muscles might also be involved since stem cell proliferation is regulated by enzymes (metalloproteinases) derived from muscular cells | Gene activity in neoblast neighbourhood contributes to proliferation/  differentiation of neoblasts. There is evidence of environmental regulation of neoblast proliferation and specification (reviewed, for example in: Rossi & Salvetti, 2019). Knockdown of the *nkx-2.2* gene in intestine/phagocytes prevents proliferation of neoblasts (Forsthoefel *et al.*, 2012). Mitotically active neoblasts are closely associated with the intestine in uninjured animals and cluster around the severed tips of intestinal branches near the plane of amputation after injury (Newmark & Sánchez Alvarado, 2000; Wenemoser & Reddien, 2010). Knockdown of the metalloproteinase mt-mmp, a homolog of mammalian mmp19, expressed in a unique subset of muscle cells situated next to large populations of neoblasts, decreases the proliferation of stem cells in response to injury (Dingwall & King, 2016). Miller & Newmark (2012) provide evidence that an insulin-like peptide (ilp-1) RNAi phenocopies the mt-mmpB RNAi exhibiting parallel decreases in the number of mitotic cells despite normal feeding behaviour. The participation of muscles in providing positional information to neoblasts has suggested a key role for musculature in regulating the fate and identity of neoblasts along the body (Witchley *et al*., 2013), becoming an important part of their microenvironment | Neoblasts in contact with phagocytic cells of the gut and muscle cells (distributed along the whole body) | Phagocytic cells of the gut (Newmark & Sánchez Alvarado, 2000; Wenemoser & Reddien, 2010; Forsthoefel *et al.*, 2012). Dingvall & King (2016) suggest that dorso-ventral muscle cells are involved in the regulation of proliferation of neighbouring stem cells (neoblasts). Ilp-1 expressed in neural tissue as well as in spermatocytes and spermatids (Miller & Newmark, 2012) might contribute to stabilize the neoblast niche | Egf signalling (Lei *et al*., (2016): Egf provided by the surrounding gut cells is required for the proliferation/differentiation of neoblasts. Dingwall & King (2016) provide evidence that the metalloproteinase mt-mmpB is involved in the regulation of stem cell proliferation. Ilp-1 RNAi shows decreases in number of mitotic cells despite normal feeding behaviour | 10–17 |
| **ANNELIDA** |  |  |  |  |  |  |
| *Capitella teleta* | Nothing known. Indirect evidence | Only evidence (indirect) for the germ line. Data originating from the study of PGCs. The cluster of putative PGCs is present as a single structure. Different observations are more consistent with referring to this structure as a PGC niche (Giani *et al.*, 2011) | The niche appears to be a permanent structure that provides a source of new PGCs through the animal’s life, and is located separate from the gonads | No data on cellular composition |  | 18 |
| *Platynereis dumerilii* | Nothing known. Indirect evidence | No direct evidence. only systemic regulators of stem cells in regeneration. It has been speculated the pygidial nerve ring and the blood sinus provide a niche signal for the SAZ (segment addition zone; where the stem cells are found). However this has not been investigated in detail. (Planques *et al.*, 2019) |  |  | Activity of a brain hormone – nereidin – was found to repress sexual maturation and to promote growth of the trunk and posterior regeneration. This is systemic regulation, and not necessarily a niche (Hauenschild, 1966; Schenk *et al.*, 2016) | 19–21 |
| **MOLLUSCA** |  |  |  |  |  |  |
| *Crassostrea gigas* | Speculated presence of a niche in the gonads | Presence of cells surrounding the germline; myoid type (Cherif-Feildel *et al*., 2019) | Germinal epithelium | Myoid cells in contact with gametes | Immunolabelling with BMP2/4 surrounding germ cells and their precursors | 22 |
| **ARTHROPODA** |  |  |  |  |  |  |
| *Pacifastacus leniusculus*  (Crustacea; crayfish) | Nothing known. Indirect evidence in haematopoietic cells | No evidence. Assumed based on the location of haematopoietic cells. Haematopoietic tissue confined to a specific domain, producing haemocytes |  | Haematopoietic tissue (HPT) from *P. leniusculus* can be easily isolated and studied *in vitro*, either as intact tissue or by isolating and culturing individual cells. HPT is composed of a series of ovoid lobules that collectively form a thin sheet on the dorsal part of the foregut Each lobule is surrounded by connective tissue and contains, among other cell types, the stem cells. To date, studies have only investigated the hematopoietic stem cells. (Lin *et al.*, 2011) | Cytokines (astakines). Only haematopoiesis has been explored | 23 |
| *Parhyale hawaiensis* (Crustacea; amphipod) | Nothing known. Indirect proof | Tentative identification of progenitors in the epithelia. (Alwes *et al.*, 2016) |  |  |  | 24 |
| **ECHINODERMATA** |  |  |  |  |  |  |
| *Antedon mediterranea* (Crinoidea) | Nothing known. Indirect evidence | Not clear. inferred niche from stem cell location. Amoebocytes in contact with the nerve cord (spatial location) move to the blastema under stress/autotomy. Niche defined by position (where the amoebocytes reside) (Candia-Carnevale *et al.*, 1997) |  | Connective tissue is in contact with amoebocytes but no proof of direct interaction |  | 25 |
| **UROCHORDATA** |  |  |  |  |  |  |
| *Ciona robusta* | No evidence yet. Branchial vessels suggested as putative niche | Circumstantial evidence. only indirect; the niche is assumed to reside in the location where progenitors are generated | Stem cells are located in branchial vessels of the adult branchial basket (Jeffery, 2019) | No clear data on the role of branchial vessel cells that border the niche | EdU activities and alkaline phosphatase and PIWI markers reported in the area (Jeffery, 2015*a*) | 26, 27 |
| *Styela plicata* | No evidence yet. Intestinal wall suggested as putative niche | Undifferentiated cycling cells in their neighbourhood. Haemoblast aggregations (Jiménez-Merino *et al*., 2019) | Putative stem cells appear in intestinal wall |  |  | 28 |
| *Botryllus schlosseri* | Well-characterized. In the endostyle and cell islands | The endostyle niche is well characterized (Voskboynik *et al*., 2008; Rosental *et al*., 2018). Rosental *et al.* (2020) propose that the haematopoietic bone marrow and the *B. schlosseri* endostyle niche evolved from a common origin.  PGC-associated in cell islands, attached to the epithelial cells of the digestive system and blood vessels and ampullae (temporary seasonal niches). | Stem cells appear in the connective tissues close to endostyle and in cell islands (Rinkevich *et al.*, 2013). | No clear data on which cells may contribute to the activities of the niche (except the stem cells themselves) | Many genes expressed in the endostyle niche (Voskoboynik *et al.*., 2008). Many identified genes are also expressed in human haematopoietic cells (Rosental *et al*., 2018). | 29–31, 33 |

BMP, bone morphogenetic protein; BrdU, bromodeoxyuridine; ECM, extracellular matrix; EdU, ethynyldeoxyuridine; EGF, epidermal growth factor; HPT, haematopoietic tissue; Hym-355, *Hydra* myoactive neuropeptide; Ilp, insulin-like peptide; Klf5a/b, Kruppel-like factor 5a/b; mt-mmp, metalloproteinase homolog of mammalian mmp19; mmp, homolog of mammalian mmp19; PGC, primordial germ cell; Piwi, P-element induced wimpy testis in *Drosophila*; RNAi, RNA interference; SAZ, segment addition zone; Wnt, wingless/integrated.

**References**

1. Martinand-Mari C, Vacelet J, Nickel M, Wörheide G, Mangeat P, Baghdiguian S. 2012. Cell death and renewal during prey capture and digestion in the carnivorous sponge *Asbestopluma hypogea* (Porifera: Poecilosclerida). J Exp Biol. 215(Pt 22):3937-43
2. Bode HR. 1996. The interstitial cell lineage of *Hydra*: a stem cell system that arose early in evolution. J Cell Sci. 109:1155-1164.
3. Bosch TC. 2009. *Hydra* and the evolution of stem cells. Bioessays. 31(4):478-86.
4. David CN, Plotnick I. 1980. Distribution of interstitial stem cells in *Hydra*. Dev Biol. 76:175-184.
5. Gahan JM, Bradshaw B, Flici H, Frank U. 2016. The interstitial stem cells in *Hydractinia* and their role in regeneration. Curr Opin Genet Dev. 40:65-73.
6. Khalturin K, Anton-Erxleben F, Milde S, Plötz C, Wittlieb J, Hemmrich G, Bosch TC. 2007. Transgenic stem cells in *Hydra* reveal an early evolutionary origin for key elements controlling self-renewal and differentiation. Dev Biol. 309(1):32-44.
7. Takahashi T, Koizumi O, Ariura Y, Romanovitch A, Bosch TC, Kobayakawa Y, Mohri S, Bode HR, Yum S, Hatta M, *et al.*. 2000. A novel neuropeptide, Hym-355, positively regulates neuron differentiation in *Hydra*. Development 127(5):997-1005.
8. Alié A, Leclère L, Jager M, Dayraud C, Chang P, Le Guyader H, Quéinnec E, Manuel M. 2011. Somatic stem cells express *Piwi* and *Vasa* genes in an adult ctenophore: ancient association of "germline genes" with stemness. Dev Biol. 350(1):183-97.
9. Presnell JS, Browne WE. 2019. Krüppel-like factor gene function in the ctenophore *Mnemiopsis* suggests an ancient role in promoting cell proliferation in metazoan stem cell niches. Biorxiv. p. 527002. doi: <https://doi.org/10.1101/527002>
10. Dingwall, CB and King, RS. 2016. Muscle-derived matrix metalloproteinase regulates stem cell proliferation in planarians. Dev Dyn. 245(9):963-70.
11. Forsthoefel DJ, James NP, Escobar DJ, Stary JM, Vieira AP, Waters FA, Newmark PA. 2012. An RNAi screen reveals intestinal regulators of branching morphogenesis, differentiation, and stem cell proliferation in planarians. Dev Cell. 23(4):691–704.
12. Lei K, Thi-Kim Vu H, Mohan RD, McKinney SA, Seidel CW, Alexander R, Gotting K, Workman JL, Sánchez Alvarado A. 2016. Egf signaling directs neoblast repopulation by regulating asymmetric cell division in planarians. Dev Cell. 38(4):413-29.
13. Miller CM, Newmark PA. 2012. An insulin-like peptide regulates size and adult stem cells in planarians. Int J Dev Biol. 56(1-3):75-82.
14. Newmark PA, Sánchez Alvarado A. 2000. Bromodeoxyuridine specifically labels the regenerative stem cells of planarians. Dev Biol. 220(2):142-53.
15. Rossi L, Salvetti A. 2019. Planarian stem cell niche, the challenge for understanding tissue regeneration. Semin Cell Dev Biol. 87:30-36.
16. Wenemoser D, Reddien PW. 2010. Planarian regeneration involves distinct stem cell responses to wounds and tissue absence. Dev Biol. 344(2):979-91.
17. Witchley JN, Mayer M, Wagner DE, Owen JH, Reddien PW. 2013. Muscle cells provide instructions for planarian regeneration. Cell Rep. 4(4):633-41.
18. Giani VC Jr, Yamaguchi E, Boyle MJ, Seaver EC. 2011. Somatic and germline expression of piwi during development and regeneration in the marine polychaete annelid *Capitella teleta*. EvoDevo. 2:10.
19. Hauenschild, C. 1966. Der hormonale Einfluss des Gehirns auf die sexuelle Entwicklung bei dem Polychaeten *Platynereis dumerilii*. Gen Comp Endocrinol. 6:26-73.
20. Planques A, Malem J, Parapar J, Vervoort M, Gazave E. 2019. Morphological, cellular and molecular characterization of posterior regeneration in the marine annelid *Platynereis dumerilii*. Dev Biol. 445(2):189-210.
21. Schenk S, Krauditsch C, Frühauf P, Gerner C, Raible F. 2016. Discovery of methylfarnesoate as the annelid brain hormone reveals an ancient role of sesquiterpenoids in reproduction. eLife. 5:e17126.
22. Cherif-Feildel M, Kellner K, Goux D, Elie N, Adeline B, Lelong C, Heude Berthelin C. 2019. Morphological and molecular criteria allow the identification of putative germ stem cells in a lophotrochozoan, the Pacific oyster *Crassostrea gigas*. Histochem Cell Biol. 151(5):419-433.
23. Lin X, Söderhäll I. Crustacean hematopoiesis and the astakine cytokines. 2011. Blood. 117(24):6417-24.
24. Alwes F, Enjolras C, Averof M. 2016. Live imaging reveals the progenitors and cell dynamics of limb regeneration. Elife. 5:e19766.
25. Candia Carnevali MD, Bonasoro F, Biale A. 1997. Pattern of bromodeoxyuridine incorporation in the advanced stages of arm regeneration in the feather star *Antedon mediterranea*. Cell Tissue Res. 289(2):363-74.
26. Jeffery WR. 2015*a*. Distal regeneration involves the age dependent activity of branchial sac stem cells in the ascidian *Ciona intestinalis*. Regeneration. 2(1):1-18.
27. Jeffery WR. 2019. Progenitor targeting by adult stem cells in *Ciona* homeostasis, injury, and regeneration. Dev Biol. 448(2):279-290.
28. Jiménez-Merino J, Santos de Abreu I, Hiebert LS, Allodi S, Tiozzo S, De Barros CM, Brown FD. 2019. Putative stem cells in the hemolymph and in the intestinal submucosa of the solitary ascidian *Styela plicata*. EvoDevo 10:31.
29. Rinkevich Y, Voskoboynik A, Rosner A, Rabinowitz C, Paz G, Oren M, Douek J, Alfassi G, Moiseeva E, Ishizuka KJ. *et al.*. 2013. Repeated, long-term cycling of putative stem cells between niches in a basal chordate. Dev Cell. 24(1):76-88.
30. Rosental B, Kowarsky M, Seita J, Corey DM, Ishizuka KJ, Palmeri KJ, Chen SY, Sinha R, Okamoto J, Mantalas G, *et al.*. 2018. Complex mammalian-like haematopoietic system found in a colonial chordate. Nature 564(7736):425-429.
31. Voskoboynik A, Soen Y, Rinkevich Y, Rosner A, Ueno H, Reshef R, Ishizuka KJ, Palmeri KJ, Moiseeva E, Rinkevich B, *et al.*. 2008. Identification of the endostyle as a stem cell niche in a colonial chordate. Cell Stem Cell 3(4):456-64.
32. Bosch, T.C.G, Anton‐Erxleben, F., Hemmrich, G., Khalturin, K. 2010. The *Hydra* polyp: nothing but an active stem cell community. Development, Growth & Differentiation 52; 15–25.
33. Rosental, B., Raveh, T., Voskoboynik, A., Weissman, I.L. 2020. Evolutionary perspective on the hematopoietic system through a colonial chordate: allogeneic immunity and hematopoiesis. Current Opinion in Immunology 62: 91-98.
