# Supplementary material for: A pan‐metazoan concept for adult stem cells: the wobbling Penrose landscape
Source: Biol Rev Camb Philos Soc. 2021 Oct 6;97(1):299–325. doi: 10.1111/brv.12801 (PMC9292022; doi:10.1111/brv.12801)
Supplement: Supplementary file 6 — Table S4. Overview of the involvement of adult stem cell (ASCs) and progenitors during homeostasis in metazoans. [file BRV-97-299-s009.docx]

# Table S4. Overview of the involvement of adult stem cells (ASCs) and progenitors during homeostasis in metazoans. Cell types and putative ASCs or progenitors involved in the processes underlying homeostasis, their level of differentiation and potency, as well as expressed ‘stemness’ gene families are reported for metazoan phyla. Although every effort was made to include all representative data, the literature is not exhaustive, particularly for vertebrates and model ecdysozoans. Red font: high levels of confidence with respect to ASC involvement in homeostatic processes. Orange font: middle levels of confidence. Black font: reported, but no level of confidence established. ASCs can be characterized as undifferentiated (U) or differentiated (D) cell types. ? = uncertain data.

| **Phylum** | **Species** | **Physiological process** | **Cellular contribution** | | | | | **Cellular process – dedifferentiation, transdifferentiation, proliferation** | **Reference** |
| --- | --- | --- | --- | --- | --- | --- | --- | --- | --- |
|  |  |  | **Cell type** | **Origin** | **‘Stemness’ gene family expression** | **Putative ASC or progenitor?** | **Potency** |  |  |
| **PORIFERA** |  |  |  |  |  |  |  |  |  |
| Demospongiae | *Ephydatia fluviatilis* | adult cell turnover | 1. archaeocytes; 2. choanocytes | U D | 1. *piwi*, *vasa/ddx*, *bruno*, *pl10*, *Tudor*; 2. *piwi* | 1.ASC; 2. ASC | totipotent/  pluripotent | 1. differentiation; 2. transdifferentiation /dedifferentiation? | 1–7 |
|  | *Halisarca caerulea* and other species | adult cell turnover | choanocytes | D | unknown | Asc? | pluripotent | unknown | 8–10 |
| Homoscleromorpha | *Oscarella lobularis* | adult cell turnover | 1. choanocytes; 2. type 2 vacuolar cells (archeocyte-like cells); 3. both cell types plus pinacocytes | D | *1. piwi*; *2. vasa*, *boule*, *bruno*; *3. piwi*, *ago*, *pl10*, *nanos*, *bruno*, *pumilio*, *tudor1* | 1.ASC; 2. ASC?; 3. ASC? | multipotent/ pluripotent? | transdifferentiation? | 11 |
| Calcarea | *Sycon ciliatum* | adult cell turnover | choanocytes | D | *myc* | ASC? | totipotent/ pluripotent | transdifferentiation | 12–14 |
| Hexactinellida | *Aphrocallistes vastus*, *Oopsacas minuta*, *Rhabdocalyptus dawsoni* | adult cell turnover | archeocytes | U? | unknown | unknown | pluripotent | differentiation | 15 |
| **PLACOZOA** |  |  |  |  |  |  |  |  |  |
|  | *Trichoplax adhaerens* | adult cell turnover | marginal cell? | D | unknown |  | unknown | unknown | 16, 17 |
| **CNIDARIA** |  |  |  |  |  |  |  |  |  |
| Hydrozoa | *Hydra attenuate*,  *Hydra* spp. | adult cell turnover | 1. interstitial cells; 2. epithelial ectodermal, epithelial endodermal | U D | *myc*, *Sox?* | 1. ASC; 2. ASC | 1. multipotent; 2. multipotent | 1. proliferation, self-renewal, asymmetric division, differentiation; 2. proliferation, self-renewal, differentiation | 18–22 |
|  | *Hydra vulgaris*, *Hydra magnipapillata*, *Hydra AEP* and *Hydra oligactis* | adult cell turnover | 1.interstitial cells; 2. epithelial  ectodermal, epithelial endodermal | U D D | unknown | 1. ASC; 2. ASC | 1. multipotent; 2. multipotent? multipotent? | 1. proliferation, differentiation; 2.proliferation, differentiation, transdifferentiation | 23–32 |
|  | *Hydra attenuata* | adult neurogenesis | interstitial cells | U | unknown | ASC? | multipotent | differentiation | 33 |
|  | *Clytia hemisphaerica* | production of stinging cells (nematocytes), neurogenesis | interstitial cells? | U | *Piwi*, *Sox* in stem and differentiated cells | ASC or early committed nematoblasts | multipotent | differentiation? | 34, 35 |
|  | *Hydractinia echinata* | clonal growth, tissue homeostasis | interstitial cells | U | *Nanos*, *vasa*, *myc*, *piwi*, *pou* | ASC? | multipotent? | proliferaation, differentiation | 36–39 |
|  | *Podocoryne carnea* | *in vitro* transdifferentiation | mononucleated epithelial striated muscle cells | D | unknown | ASC? | multipotent | transdifferentiation | 40, 41 |
| Scyphozoa | *Aurelia* sp.1 | cnidoblast development through the life cycle | epitheliomuscular cells | D | unknown | progenitor? | unipotent??? | unknown | 42 |
| Cubozoa | *Tripedalia cystophora* | adult rhopalia cell turnover | unknown | unknown | unknown | unknown | unknown | proliferation | 43, 44 |
| Hexacorallia | *Nematostella vectensis* | adult neurogenesis | unknown | unknown | unknown | unknown | unknown | unknown | 33 |
| **CTENOPHORA** |  |  |  |  |  |  |  |  |  |
| Cydippida | *Pleurobrachia pileus* | adult cell turnover | somatic cells of tentacle root, comb rows, aboral sensory complex | U | *Vasa*, *Piwi*, *PL10*, *Bruno*, *Sox* | stem cells/ undifferentiated progenitors | unknown | proliferation, differentiation? | 45 |
| Lobata | *Mnemiopsys leidyi* | adult cell turnover | unknown | unknown | unknown | unknown | unknown | unknown | 46 |
| **ACOELOMORPHA** |  |  |  |  |  |  |  |  |  |
|  | *Isodiametra pulchra* | adult cell turnover | neoblast | U | *piwi* | ASC | totipotent or pluripotent | proliferation, differentiation | 47, 48 |
|  | *Convolutriloba longifissura* | adult cell turnover | neoblasts, two types | U | unknown | ASC? | pluripotent | proliferation, differentiation | 49 |
|  | *Hofstenia miamia* | adult cell turnover | neoblasts | U | unknown | ASC? | unknown | unknown | 50 |
| **PLATYHELMINTHES** |  |  |  |  |  |  |  |  |  |
| Tricladida | *Schmidtea mediterranea* | adult cell turnover | sigma neoblast, zeta- and gamma-neoblasts | U | *piwi* | ASC | pluripotent? | 1. self-renewal, differentiation; 2. differentiation | 51–56 |
| Macrostomorpha | *Macrostomum* sp. *Macrostomum lignano* | parenchymal cell turnover | neoblasts | U | unknown | ASC? | pluripotent??? | proliferation, apoptosis | 57, 58 |
| **NEMERTEA** |  |  |  |  |  |  |  |  |  |
| Heteronemertea | *Micrura alaskensis* (*Maculaura alaskensi*) | pilidium growth, normal growth | unknown | unknown | unknown | unknown | unknown | unknown | 59, 60 |
| **ANNELIDA** |  |  |  |  |  |  |  |  |  |
| Sedentaria | *Capitella* sp. I | special structure in the coelomic cavity of segments 5 and 6 | uncharacterized cells | U? | *vasa*, *nanos, piwi* | unknown | thought to be multipotent | unknown | 61, 62 |
|  | **Enchytraeus japonensis* | Adult animal growth | neoblasts, mesodermal N-cell | U,D | unknown | ASC? | thought to be multipotent | proliferation | 63 |
|  | *Arenicola marina* | maintenance of coelomic fluid cells | juvenile cells and young amoebocytes | U, D | unknown | progenitor? | unipotent | proliferation, differentiation | 64 |
| Errantia | *Dorvillea bermudensis* | Adult animal cell turnover | unknown | unknown | unknown | unknown | unknown | proliferation | 65 |
|  | *Platynereis dumerilii* | adult posterior elongation | posterior ectodermal and mesodermal cells, teloblast-like cells | D? | *piwi*, *vasa*, *pl10*, *nanos*, *myc* | unknown | supposed to be multipotent | proliferation | 66 |
| **BRACHIOPODA** |  |  |  |  |  |  |  |  |  |
|  | no data |  |  |  |  |  |  |  |  |
| **PHORONIDA** |  |  |  |  |  |  |  |  |  |
|  | no data |  |  |  |  |  |  |  |  |
| **ECTOPROCTA** |  |  |  |  |  |  |  |  |  |
|  | *Bugula neritina* | body growth | unknown | unknown | unknown | unknown | unknown | proliferation | 67 |
| **GASTROTRICHA** |  |  |  |  |  |  |  |  |  |
|  | no data |  |  |  |  |  |  |  |  |
| **MOLLUSCA** |  |  |  |  |  |  |  |  |  |
| Bivalvia | *Pinctada fucata* | enlargement of the body surface and shell | uncharacterized cells | unknown | unknown | unknown | unknown | proliferation | 68 |
|  | *Crenomytilus grayanus* | myodifferentiation | satellite-like cells | U | unknown | unknown | unknown | unknown | 69 |
|  | *Mytilus galloprovincialis* | cell replacement in the digestive gland | epithelial cells of the stomach | D | unknown | unknown | unknown | proliferation | 70 |
|  | *Tapes philippinarum* | haematopoesis | circulating haemoblasts | U | unknown | progenitor? | unknown | proliferation, differentiation | 71 |
| Cephalopoda | *Octopus vulgaris*, *O. briareus*, *Sepia officinalis*, *Euprymna tasmanica* | haematopoesis | leukoblasts (haemocytes) | U? | unknown | unknown | unknown | differentiation? | 72 |
| Gastropoda | *Littorina littorea* | haematopoesis | circulating haemoblast precursors | U | unknown | progenitor? | unknown | proliferation | 72, 73 |
| **ENTOPROCTA** |  |  |  |  |  |  |  |  |  |
|  | no data |  |  |  |  |  |  |  |  |
| **CHAETOGNATHA** |  |  |  |  |  |  |  |  |  |
|  | *Spadella cephaloptera* | neurogenesis in adult? | epidermal cells, corona ciliata in adult | D | unknown | unknown | unknown | proliferation | 74 |
| **ROTIFERA** |  |  |  |  |  |  |  |  |  |
|  | *Brachionus calycifloruss* | adult cell turnover | unknown | unknown | unknown | unknown | unknown | unknown | 75 |
| **ARTHROPODA** |  |  |  |  |  |  |  |  |  |
| Crustacea | *Procambarus clarkii* | continuous production of new neurons | neurogenic stem cells | unknown | unknown | stem cells? | unknown | migration, proliferation | 76, 100 |
|  | *Astacus astacus* | heart and skeletal musculature | satellite cells | U | unknown | unknown | unknown | unknown | 77 |
|  | *Scylla paramamosain* | intestinal tract functioning (hepatopancreas) | E-cells | U? | unknown | unknown | multipotent? | unknown | 78 |
|  | *Pacifastacus leniusculus* | haematopoesis | haematopoietic stem cells | U | unknown | unknown | unknown | proliferation | 79 |
| Pycnogonida | *Pseudopallene* sp. | adult neurogenesis | ectodermal cells | D? | unknown | unknown | unknown | proliferation, migration | 81 |
| **ONYCHOPHORA** |  |  |  |  |  |  |  |  |  |
|  | *Euperipatoides rowelli* | cell turnover in the genital tract | unknown | unknown | unknown | unknown | unknown | proliferation, apoptosis | 80 |
| **TARDIGRADA** |  |  |  |  |  |  |  |  |  |
|  | *Hypsibius dujardini* | homeostatic cell turnover | midgut epithelium cells | D? | unknown | unknown | unknown | proliferation | 82 |
| **NEMATODA** |  |  |  |  |  |  |  |  |  |
|  | *Caenorhabditis elegans* | somatic tissue | adult postmitotic cells | D? | unknown | no somatic stem cells only germ stem cells | unknown | no proliferation | 83 |
| **PRIAPULIDA** |  |  |  |  |  |  |  |  |  |
|  | no data |  |  |  |  |  |  |  |  |
| **XENOTURELLIDA** |  |  |  |  |  |  |  |  |  |
|  | no data |  |  |  |  |  |  |  |  |
| **ECHINODERMATA** |  |  |  |  |  |  |  |  |  |
| Holothuroidea | *Holothuria glaberrima* | physiological regeneration of digestive tube | 1. vesicular enterocytes of the luminal epithelium; 2. myoepithelial cells of the mesothelium | D | *Myc* | specialised progenitors | multipotent? | 1.proliferation, dedifferentiation;  2. dedifferentiation, migration, proliferation | 84–86 |
|  | *Eupentacta fraudatrix* | physiological regeneration of digestive tube | vesicular enterocytes of the luminal epithelium | D | unknown | unknown | unknown | proliferation | 87 |
|  | *Eupentacta fraudatrix Apostichopus japonicus* | maintenance of coelomocyte population | small motile cells in the connective tissue of several organs | U | unknown | undifferentiated progenitor | multipotent? | proliferation, differentiation | 88 |
|  | *Holothuria glaberrima* | adult neurogenesis | ciliated epithelial cells of radial glia | D | *Sox*, *Pou*, *Klf*, *Myc*, *Piwi* | specialised progenitor | unipotent? | proliferation, dedifferentiation? | 89–91 |
| Echinoidea | *Lytechinus variegatus* | homeostatic cell turnover | cells of oesophagus, radial nerve, and a sub-population of coelomocytes | D | *Vasa* | unknown | multipotent? | unknown | 92 |
| Ophiuroidea | *Amphiura filiformis* | normal non-regenerating arms | cells of epidermis, podia, radial water canal in normal non-regenerating arms | D | unknown | unknown | unknown | proliferation | 93 |
| Crinoidea | *Antedon mediterranea* | homeostatic cell turnover | amoebocytes, coelomocytes | U | unknown | undifferentiated progenitor? | multipotent? | unknown | 94 |
| Asteroidea | *Asterias rubens*, *Asterias amurensis* | maintenance of coelomocyte population | small coelomic epithelial cells, cells of axial organ and Tiedemann body | U | unknown | undifferentiated progenitor? | unipotent? | proliferation, differentiation | 95–100 |
| **HEMICHORDATA** |  |  |  |  |  |  |  |  |  |
| Enteropneusta | *Saccoglossus mereschkowskii* | homeostatic cell turnover | cells of skin and intestinal epithelium | D | unknown | unknown | unknown | proliferation | 101 |
| **UROCHORDATA** |  |  |  |  |  |  |  |  |  |
| Stolidobranchia | *Botryllus schlosseri* | weekly replacement of old generation of zooids with a new generation of asexual developing buds | 1. epithelial cells; 2. haemoblasts in endostyle niche | U U | *vasa*, *piwi*, *pl10*, *Pou* | 2. ASC | multipotent? | 2. proliferation | 102–105 |
| **CEPHALOCHORDATA** |  |  |  |  |  |  |  |  |  |
|  | no data |  |  |  |  |  |  |  |  |
| **VERTEBRATA** |  |  |  |  |  |  |  |  |  |
| Cyprinidae | *Danio rerio* | adult neurogenesis | adult neural glial stem cells (ANSCs) | D | unknown | ASC | unknown | self-renewal, transdifferentiation?, asymmetric division | 106, 107 |
| Amphibia | *Ambystoma mexicanum* | uninjured spinal cord | ependymoglial cells | D | unknown | unknown | unknown | proliferation | 108 |
|  | *Ambystoma mexicanum* | uninjured adult limb | connective tissue (CT) cells – skeleton, periskeleton, tendon, dermis, interstitial fibroblasts | D | unknown | progenitor | multipotent? | dedifferentiation, proliferation, re-differentiation | 109 |
|  | *Xenopus laevis* | adult small intestinal epithelium | columnar and goblet cells? | D | unknown | unknown | unknown | proliferation | 110 |

**References**

1. Simpson TL. 1984. The cell biology of sponges. New York (NY): Springer.
2. Ereskovsky A. 2010. The comparative embryology of sponges. Dordrecht: Springer.
3. Funayama N. 2018. The cellular and molecular bases of the sponge stem cell systems underlying reproduction, homeostasis and regeneration. Int J Dev Biol. 62: 513-525.
4. Funayama N, Nakatsukasa M, Mohri K, Masuda Y, Agata K. 2010. Piwi expression in archeocytes and choanocytes in demosponges: Insights into the stem cell system in demosponges. Evol Dev. 12: 275-287.
5. Okamoto K, Nakatsukasa M, Alié A, Masuda Y, Agata K, Funayama N. 2012. The active stem cell specific expression of sponge Musashi homolog EflMsiA suggests its involvement in maintaining the stem cell state. Mech Develop. 129: 24-37.
6. Alié A, Hayashi T, Sugimura I, Manuel M, Sugano W, Mano A, Satoh N, Agata K, Funayama N. 2015. The ancestral gene repertoire of animal stem cells. Proc Natl Acad Sci USA. 112(51):E7093-100.
7. Funayama N. 2013. The stem cell system in demosponges: Suggested involvement of two types of cells: archeocytes (active stem cells) and choanocytes (food-entrapping flagellated cells). Dev Genes Evol. 223(1-2):23–38.
8. De Goeij JM, De Kluijver A, Van Duyl FC, Vacelet J, Wijffels RH, De Goeij A FPM, Cleutjens JPM, Schutte B. 2009. Cell kinetics of the marine sponge *Halisarca caerulea* reveal rapid cell turnover and shedding. J Exp Biol. 212:3892-3900.
9. Alexander BE, Liebrand K, Osinga R, van der Geest HG, Admiraal W, Cleutjens JP, Schutte B, Verheyen F, Ribes M, van Loon E, de Goeij JM. 2014. Cell turnover and detritus production in marine sponges from tropical and temperate benthic ecosystems. PLoS ONE. 9(10):e109486.
10. Kenny NJ, de Goeij JM, de Bakker DM, Whalen CG, Berezikov E, Riesgo A. 2018. Towards the identification of ancestrally shared regenerative mechanisms across the Metazoa: A transcriptomic case study in the demosponge *Halisarca caerulea*. Mar Genomics. 37:135-147.
11. Fierro-Constaín L, Schenkelaars Q, Gazave E, Haguenauer A, Rocher C, Ereskovsky A, Borchiellini C, Renard E. 2017. The conservation of the germline multipotency program, from sponges to vertebrates: A stepping stone to understanding the somatic and germline origins. Genome Biol Evol. 9(3):474-488.
12. Adamska M, Degnan BM, Green K, Zwafink C. 2011. What sponges can tell us about the evolution of developmental processes. Zoology (Jena). 114:1–10.
13. Adamska M. 2016. Sponges as models to study emergence of complex animals. Curr Opin Genet Dev. 39:21-28.
14. Fortunato SAV, Vervoort M, Adamski M, Adamska M. 2016. Conservation and divergence of bHLH genes in the calcisponge *Sycon ciliatum*. EvoDevo. 7:23.
15. Leys SP, Mackie GO, Reiswig HM. 2007. The biology of glass sponges. Adv Mar Biol. 52:1-145.
16. Jakob W, Sagasser S, Dellaporta S, Holland P, Kuhn K, Schierwater B. 2004. The Trox-2 Hox/ParaHox gene of Trichoplax (Placozoa) marks an epithelial boundary. Dev Genes Evol. 214:170-175.
17. Guidi L, Eitel M, Cesarini E, Schierwater B, Balsamo M. 2011. Ultrastructural analyses support different morphological lineages in the Placozoa Grell, 1971. J Morphol. 272:371-378.
18. Bosch TC, Anton-Erxleben F, Hemmrich G, Khalturin K. 2010. The hydra polyp: nothing but an active stem cell community. Dev Growth Differ. 52:15-25.
19. Watanabe H, Hoang VT, Mattner R, Holstein TW. 2009. Immortality and the base of multicellular life: lessons from cnidarian stem cells. Semin Cell Dev Biol. 20:1114-1125.
20. Hobmayer B, Jenewein M, Eder D, Eder MK, Glasauer S, Gufler S, Hartl M, Salvenmoser W. 2012. Stemness in hydra – a current perspective. Int J Dev Biol. 56:509-517.
21. Bode HR, Flick KM, Smith GS. 1976. Regulation of interstitial cell differentiation in *Hydra attenuata*. I. Homeostatic control of interstitial cell population size. J Cell Sci. 20: 29-46.
22. David C N, Plotnick I. 1980. Distribution of interstitial stem cells in *Hydra*. Dev Biol. 76(1):175-184
23. Gierer A, Berking S, Bode H, David CN, Flick K, Mhansmann G, Schaller H, Trenkner E. 1972. Regeneration of Hydra from reaggregated cells. Nat New Biol. 239:98-101.
24. Sugiyama T, Fujisawa T. 1978. Genetic analysis of developmental mechanisms in *Hydra*. II. Isolation and characterization of an interstitial cell-deficient strain. J Cell Sci. 29:35-52.
25. David CN, Murphy S. 1977. Characterization of interstitial stem cells in *Hydra* by cloning. Dev Biol. 58:372-383.
26. Schmidt T, David CN. 1986. Gland cells in Hydra: cell cycle kinetics and development. J Cell Sci. 85:197-215.
27. Bosch TC, David CN. 1987. Stem cells of *Hydra magnipapillata* can differentiate into somatic cells and germ line cells. Dev Biol. 121:182-191.
28. Siebert S, Anton-Erxleben F, Bosch TCG. 2008. Cell type complexity in the basal metazoan *Hydra* is maintained by both stem cell based mechanisms and transdifferentiation. Dev Biol. 313:13-24.
29. Buzgariu W, Crescenzi M, Galliot B. 2014. Robust G2 pausing of adult stem cells in *Hydra*. Differentiation. 87:83-99.
30. David CN. 2012. Interstitial stem cells in *Hydra*: multipotency and decision-making. Int J Dev Biol. 56:489-497.
31. Buzgariu W, Al Haddad S, Tomczyk S, Wenger Y, Galliot B. 2015. Multifunctionality and plasticity characterize epithelial cells in *Hydra*. Tissue Barriers. 3(4):e1068908.
32. Siebert S, Farrell J, Jack F. J, Abeykoon Y, Primack A, Schnitzler C, Juliano C. 2019. Stem cell differentiation trajectories in *Hydra* resolved at single-cell resolution. Science. 365:6451.
33. Rentzsch F, Layden M, Manuel M. 2017. The cellular and molecular basis of cnidarian neurogenesis. Wiley Interdiscip Rev Dev Biol. 6(1):e257.
34. Denker E, Manuel M, Leclère L, Le Guyader H, Rabet N. 2008. Ordered progression of nematogenesis from stem cells through differentiation stages in the tentacle bulb of *Clytia hemisphaerica* (Hydrozoa, Cnidaria). Dev Biol. 315:99-113.
35. Jager M, Quéinnec E, Le Guyader H, Manuel M. 2011. Multiple Sox genes are expressed in stem cells or in differentiating neuro-sensory cells in the hydrozoan *Clytia hemisphaerica*. EvoDevo. 2:12.
36. Duffy DJ, Plickert G, Ku¨ nzel T, Tilmann W, Frank U. 2010. Wnt signalling promotes oral but suppresses aboral structures in *Hydractinia* metamorphosis and regeneration. Development. 137:3057-3066.
37. Müller WA, Teo R, Frank U. 2004. Totipotent migratory stem cells in a hydroid. Dev Biol. 275(1):215-24.
38. Gahan JM, Bradshaw B, Flici H, Frank U. 2016. The interstitial stem cells in *Hydractinia* and their role in regeneration. Curr Opin Genet Dev. 40:65-73.
39. Plickert G, Frank U, Müller WA. 2012. *Hydractinia*, a pioneering model for stem cell biology and reprogramming somatic cells to pluripotency. Int J Dev Biol. 56(6-8):519-34.
40. Schmid V, Wydler M, Alder H. 1982. Transdifferentiation and regeneration *in vitro*. Dev Biol. 92:476-488.
41. Schmid V, Alder H, Plickert G, Weber C. 1988. Transdifferentiation from striated muscle of medusae in vitro. Cell Differ Dev. 25:137-146.
42. Gold DA, Lau CLF, Fuong H, Kao G, Hartenstein V, Jacobs DK. 2019. Mechanisms of cnidocyte development in the moon jellyfish *Aurelia*. Evol Dev. 21(2):72‐81.
43. Gurska, D, Garm A. 2014. Cell proliferation in cubozoan jellyfish *Tripedalia cystophora* and *Alatina moseri*. PLoS ONE. 9:e102628.
44. Skogh C, Garm A, Nilsson DE, Ekström. 2006. Bilaterally symmetrical rhopalial nervous system of the box jellyfish *Tripedalia cystophora*. J Morphol. 267(12):1391-405.
45. Alié A, Leclère L, Jager M, Dayraud C, Chang P, Le Guyader H, Quéinnec E, Manuel M. 2011. Somatic stem cells express Piwi and Vasa genes in an adult ctenophore: Ancient association of “germline genes” with stemness. Dev Biol. 350:183-197.
46. Schnitzler C, Simmons D, Pang K, Martindale M, Baxevanis A. 2014. Expression of multiple Sox genes through embryonic development in the ctenophore *Mnemiopsis leidyi* is spatially restricted to zones of cell proliferation. EvoDevo. 5:15.
47. De Mulder K, Kuales G, Pfister D, Willems M, Egger B, Salvenmoser W, Thaler M, Gorny AK, Hrouda M, Borgonie G et al. 2009*a*. Characterization of the stem cell system of the acoel *Isodiametra pulchra*. BMC Dev Biol. 9:69.
48. Egger B, Steinke D, Tarui H, De Mulder K, Arendt D, Borgonie G, Funayama N, Gschwentner R, Hartenstein V, Hobmayer B, et al. 2009*b*. To be or not to be a flatworm: the acoel controversy. PLoS ONE. 4:e5502.
49. Gschwentner R, Ladurner P, Nimeth K, Rieger R. 2001. Stem cells in a basal bilaterian. S-phase and mitotic cells in *Convolutriloba longifissura* (Acoela, Platyhelminthes). Cell Tissue Res. 304(3):401-408.
50. Srivastava M, Mazza-Curll KL, van Wolfswinkel JC, Reddien PW. 2014. Whole-body acoel regeneration is controlled by Wnt and BmpAdmp signaling. Curr Biol. 24:1107-1113.
51. van Wolfswinkel JC, Wagner DE, Reddien PW. 2014. Single-cell analysis reveals functionally distinct classes within the planarian stem cell compartment. Cell Stem Cell. 15(3):326-339.
52. Pellettieri J, Sánchez Alvarado A. 2007. Cell turnover and adult tissue homeostasis: from humans to planarians. Annu Rev Genet. 41:83-105.
53. Lai AG, Kosaka N, Abnave P, Sahu S, Aboobaker AA. 2018. The abrogation of condensin function provides independent evidence for defining the self-renewing population of pluripotent stem cells. Dev Biol. 433:218-226.
54. Newmark PA, Sanchez Alvarado A. 2002. Not your father’s planarian: a classic model ´enters the era of functional genomics. Nat Rev Genet. 3:210–19.
55. Reddien PW, Oviedo NJ, Jennings JR, Jenkin JC, Sanchez Alvarado A. 2005. SMEDWI-2 is a PIWI-like protein that regulates planarian stem cells. Science. 310:1327-30.
56. Reddien PW, Sanchez Alvarado A. 2004. Fundamentals of planarian regeneration. Ann Rev Cell Dev Biol. 20:725-57.
57. Nimeth K, Ladurner P, Gschwentner R, Salvenmoser W, Rieger R. 2002. Cell renewal and apoptosis in *Macrostomum sp*. [Lignano]. Cell Biol Int. 26(9):801-15.
58. Mouton S, Wudarski J, Grudniewska M, Berezikov E. 2018. The regenerative flatworm *Macrostomum lignano*, a model organism with high experimental potential. Int J Dev Biol. 62:551-558.
59. Bely A, Zattara E, Sikes J. 2014. Regeneration in spiralians: evolutionary patterns. Int J Dev Biol. 58: 623-634.
60. Bird A, von Dassow G, Maslakova S. 2014. How the pilidium larva grows. EvoDevo. 5:13.
61. Giani VC Jr, Yamaguchi E, Boyle MJ, Seaver EC. 2011. Somatic and germline expression of piwi during development and regeneration in the marine polychaete annelid *Capitella teleta*. EvoDevo. 2:10.
62. Dill KK, Seaver EC. 2008. Vasa and nanos are coexpressed in somatic and germ line tissue from early embryonic cleavage stages through adulthood in the polychaete *Capitella sp*. I. Dev Genes Evol. 218:453-463.
63. Sugio M, Yoshida-NoroC, Ozawa K and Tochinai S. 2012. Stem cells in asexual reproduction of *Enchytraeus japonensis* (Oligochaeta, Annelid): Proliferation and migration of neoblasts. Dev Growth Differ. 54:439-450.
64. Persinina M, Chaga O. 1995. Renewal and differentiation of coelomic fluid cells in polychaeta *Arenicola marina*. III. Autoradiographic analysis. Cytology (Russ). 37:101-105.
65. Paulus T, Müller MCM. 2006. Cell proliferation dynamics and morphological differentiation during regeneration in *Dorvillea bermudensis* (Polychaeta, Dorvilleidae) J Morphol. 267(4):393-403.
66. [Gazave E, Béhague J, Laplane L, Guillou A, Préau L, Demilly A, Guillaume Balavoine G, Vervoort M. 2013. Posterior elongation in the annelid P*latynereis dumerilii* involves stem cells molecularly related to primordial germ cells. Dev Biol. 382(1):246-67.](https://www.ncbi.nlm.nih.gov/pubmed/?term=Posterior+elongation+in+the+annelid+Platynereis+dumerilii+involves+stem+cells+molecularly+related+to+primordial+germ+cell)
67. Fuchs J, Martindale M, and Hejnol A. 2011. Gene expression in bryozoan larvae suggest a fundamental importance of pre-patterned blastemic cells in the bryozoan life-cycle. EvoDevo. 2:13.
68. Fang Z, Feng Q, Chi Y, Xie L, Zhang R. 2008. Investigation of cell proliferation and differentiation in the mantle of *Pinctada fucata* (Bivalve, Mollusca). Mar Biol. 153:745-754.
69. Martynova M G. 2004. Proliferation and differentiation processes in the heart muscle elements in different phylogenetic groups. Int Rev Cytol. 235:215-250.
70. Zaldibar B, Cancio I, Marigómez I. 2004. Circatidal variation in epithelial cell proliferation in the mussel digestive gland and stomach. Cell Tissue Res. 318:395-402.
71. Matozzo V, Marin M G, Cima F, Ballarin L. 2008. First evidence of cell division in circulating haemocytes from the Manila clam *Tapes philippinarum*. Cell Biol Int. 32:865-868.
72. Pila EA, Sullivan JT, Wu XZ, Fang J, Rudko SP, Gordy MA, Hanington PC. 2016. Haematopoiesis in molluscs: a review of haemocyte development and function in gastropods, cephalopods and bivalves. Dev Comp Immunol. 58:119-128.
73. Gorbushin AM, Yakovleva NV. 2006. Haemogram of *Littorina littorea*. J Mar Biol Assoc UK. 86:1175.
74. Perez Y, Rieger V, Martin E, Müller CH G, Harzsch S. 2013. Neurogenesis in an early protostome relative: Progenitor cells in the ventral nerve center of chaetognath hatchlings are arranged in a highly organized geometrical pattern. J Exp Zool B Mol Dev Evol. 320(3):179-193.
75. Paraskevopoulou S, Dennis AB, Weithoff G, Hartmann S, Tiedemann R. 2019. Within species expressed genetic variability and gene expression response to different temperatures in the rotifer *Brachionus calyciflorus sensu stricto*. PLoS ONE. 14(9):e0223134.
76. Zhang Y, Allodi S, Sandeman DC Beltz BS. 2009. Adult neurogenesis in the crayfish brain: proliferation, migration and possible origin of precursor cells. Dev Neurobiol. 69(7):415-436.
77. Martynova MG. 1993. Satellite cells in the crayfish heart muscle functions as stem cells and are characterized by molt dependent behaviour. Zool Anz. 230:181-190.
78. Zeng H, Ye H, Li S, Wang G, Huang J. 2010. Hepatopancreas cell cultures from mud crab, *Scylla paramamosain*. In Vitro Cell Dev Biol Anim. 46:431-37.
79. Söderhäll I, Bangyeekhun E, Mayo S, Söderhäll K. 2003. Hemocyte production and maturation in an invertebrate animal; proliferation and gene expression in hematopoietic stem cells of *Pacifastacus leniusculus*. Dev Comp Immunol. 27:661-672.
80. Treffkorn S, Hernández-Lagos O, and Mayer G. 2019. Evidence for cell turnover as the mechanism responsible for the transport of embryos towards the vagina in viviparous onychophorans (velvet worms) Front Zool. 16:16.
81. Brenneis G, Scholtz G. 2014. The ‘ventral organs’ of Pycnogonida (Arthropoda) are neurogenic niches of late embryonic and post-embryonic nervous system development. PLoS ONE. 9(4): e95435.
82. Gross V, Bährle R, Mayer G. 2018. Detection of cell proliferation in adults of the water bear *Hypsibius dujardini* (Tardigrada) via incorporation of a thymidine analog. Tissue Cell. 51:77-83.
83. Kipreos E, van den Heuvel S. 2019. Developmental control of the cell cycle: Insights from *Caenorhabditis elegans*. Genetics. 211:797-829.
84. Mashanov VS, García-Arrarás JE. 2011. Gut regeneration in holothurians: A snapshot of recent developments. Biol Bull. 221:93-109.
85. Mashanov, VS, Zueva O, Garcı́a-Arrarás J E. 2014*a*. Postembryonic organogenesis of the digestive tube: why does it occur in worms and sea cucumbers but fail in humans? Curr Top Dev Biol. 108:185-216.
86. Mashanov V, Zueva O, Mashanova D, García-Arrarás JE. 2017. Expression of stem cell factors in the adult sea cucumber digestive tube. Cell Tissue Res. 370(3):427-440.
87. Mashanov V, Frolova L, Dolmatov I. 2004. Structure of the digestive tube in the holothurian *Eupentacta fraudatrix* (Holothuroidea:Dendrochirota). Russ J Mar Biol. 30:314–322.
88. Eliseikina MG, Magarlamov TY, Dolmatov IY. 2010. Stem cells of holothuroid coelomocytes. In: Harris LG, Bottger SA, Walker CW, Lesser MP, editors. Echinoderms: Durham. Boca Raton: CRC Press. p. 163-166.
89. Mashanov VS, Zueva OR, Garcı́a-Arrarás JE. 2015*b*. Heterogeneous generation of new cells in the adult echinoderm nervous system. Front Neuroanat. 9:123.
90. Mashanov V, Zueva O. 2019. Radial glia in Eehinoderms. Dev Neurobiol. 79(5):396-405.
91. Mashanov V, Zueva O, Garcia-Arraras LE. 2014*b*. Transcriptomic changes during regeneration of the central nervous system in an echinoderm. BMC Genomics. 15:357.
92. Reinardy HC, Emerson CE, Manley JM Bodnar AG. 2015. Tissue regeneration and biomineralization in sea urchins: Role of Notch signaling and presence of stem cell markers. PLoS ONE 10(8):e0133860.
93. Czarkwiani A, Ferrario C, Dylus DV, Sugni M, Oliveri P. 2016. Skeletal regeneration in the brittle star *Amphiura filiformis*. Front Zool. 13:18.
94. Di Benedetto C, Parma L, Barbaglio A, Sugni M, Bonasoro F, Carnevali MD. 2014. Echinoderm regeneration: an *in vitro* approach using the crinoid *Antedon mediterranea*. Cell Tissue Res. 358(1):189-201.
95. Sharlaimova N, Pinaev G, Petukhova O. 2010. Comparative analysis of behavior and proliferative activity in culture of cells of coelomic fluid and of cells of various tissues of the sea star *Asterias rubens L.* isolated from normal and injured animals. Cell Tissue Biol. 4:280-288.
96. Sharlaimova NS, Petukhova OA. 2012. Characteristics of populations of the coelomic fluid and coelomic epithelium cells from the starfish *Asterias rubens L.* able to attach to and spread on various substrates. Cell Tissue Biol. 6:176-188.
97. Sharlaimova N, Shabelnikov S, Petukhova O. 2014. Small coelomic epithelial cells of the starfish *Asterias rubens L*. that are able to proliferate *in vivo* and *in vitro*. Cell Tissue Res. 356:83-64.
98. Sharlaimova N S, Petukhova OA. 2016. The small cells of coelomic fluid and coelomic epithelium isolated from starfish *Asterias rubens* and *Asterias amurensis* (Echinodermata: Asteroidea): comparative analysis of cell morphology and proliferative activity in vivo and in vitro. Russ J Mar Biol. 42(2):199-203.
99. Holm K, Dupont S, Skold H, Stenius A, Thorndyke M, Hernroth B. 2008. Induced cell proliferation in putative haematopoietic tissues of the sea star, *Asterias rubens (L*.). J Exp Biol. 211:2551-2558.
100. Vogt G. 2012. Hidden treasures in stem cells of indeterminately growing bilaterian invertebrates. Stem Cell Rev Rep. 8:305-317.
101. Stolyarova M, Valkovich E. 2016. The mechanism of physiological regeneration in the skin and intestinal epithelia of *Saccoglossus mereschkowskii* (Enteropneusta, Hemichordata) Zh Evol Biokhim Fiziol. (Russ). 52(1):84-86.
102. Manni L, Zaniolo G, Cima F, Burighel P, Ballarin L. 2007. *Botryllus schlosseri*: a model ascidian for the study of asexual reproduction. Dev Dyn. 236:335-352.
103. Voskoboynik A, Soen Y, Rinkevich Y, Rosner A, Ueno H, Reshef R, Ishizuka KJ, Palmeri KJ, Moiseeva E, Rinkevich B, Weissman IL. 2008. Identification of the endostyle as a stem cell niche in a colonial chordate. Cell Stem Cell. 3(4):456-64.
104. Brown FD, Tiozzo S, Roux MM, Ishizuka K, Swalla BJ, Anthony W. De Tomaso AW. 2009*b*. Early lineage specification of long-lived germline precursors in the colonial ascidian *Botryllus schlosseri*. Development. 136:3485-3494.
105. Rinkevich Y, Voskoboynik A, Rosner A, Rabinowitz C, Paz G, Oren M, Douek J, Alfassi G, Moiseeva E, Ishizuka K, et al. 2013. Repeated, long-term cycling of putative stem cells between niches in a basal chordate. Dev Cell. 24(1):76-88.
106. Barbosa JS, Sanchez-Gonzalez R, Di Giaimo R, Baumgart E V, Theis FJ, Gotz M, Ninkovic J. 2015. Neurodevelopment. Live imaging of adult neural stem cell behavior in the intact and injured zebrafish brain. Science. 348:789-793.
107. Dray N, Bedu S, Vuillemin N, Alunni A, Coolen M, Krecsmarik M, Supatto W, Beaurepaire E, Bally-Cuif, L. 2015. Large-scale live imaging of adult neural stem cells in their endogenous niche. Development. 142(20):3592-3600.
108. Freitas P, Lovely A, Monaghan J. 2019. Investigating Nrg1 signaling in the regenerating axolotl spinal cord using multiplexed FISH. Dev Neurobiol. 79(5):453-467.
109. Gerber T, Murawala P, Knapp D, Masselink W, Schuez M, Hermann S, Gac-Santel M, Nowoshilow S, Kageyama J, Khattak S. et al. 2018. Single-cell analysis uncovers convergence of cell identities during axolotl limb regeneration. Science. 362:6413.
110. McAvoy JW, Dixon KE. 1977. Cell proliferation and renewal in the small intestinal epithelium of metamorphosing and adult *Xenopus laevis*. J Exp Zool. 202:129-138.
