# Supplementary material for: A pan‐metazoan concept for adult stem cells: the wobbling Penrose landscape
Source: Biol Rev Camb Philos Soc. 2021 Oct 6;97(1):299–325. doi: 10.1111/brv.12801 (PMC9292022; doi:10.1111/brv.12801)
Supplement: Supplementary file 7 — Table S5. Overview of the involvement of adult stem cell (ASCs) and progenitors in regeneration processes in metazoans. [file BRV-97-299-s001.docx]

**Table S5.** Overview of the involvement of adult stem cells (ASCs) and progenitors in regeneration processes in metazoans. The presence and type of regeneration, the cell types and putative ASCs or progenitors involved, their level of differentiation and potency, as well as expressed ‘stemness’ gene families are reported for metazoan phyla. Regeneration modes include whole-body regeneration (WBR), antero/posterior axis regeneration (A or P), appendage regeneration and organ regeneration. Although every effort was made to include all representative data for each phylum, the literature is not exhaustive, particularly for vertebrates and model ecdysozoans. Red font: high levels of confidence with respect to ASC contribution. Orange font: middle levels of confidence. Black font: reported, but no level of confidence established. ASCs can be characterized as undifferentiated (U) or differentiated (D) cell types. ? = uncertain data.

| **Phylum** | **Species** | **Regeneration mode – WBR, A/P, appendage, organ** | **Cellular contributions** | | | | | **Cellular process - dedifferentiation, transdifferentiation** | **Reference** |
| --- | --- | --- | --- | --- | --- | --- | --- | --- | --- |
|  |  |  | **Cell types** | **Origin** | **"Stemness" gene expression** | **putative ASC or progenitor cells?** | **Potency** |  |  |
| **PORIFERA** |  |  |  |  |  |  |  |  |  |
| Demospongiae | *Halisarca dujardini* | WBR | archaeocytes, choanocytes | U, D | unknown | yes = archaeocytes, choanocytes | thought to be pluripotent | transdifferentiation | 1, 2 |
|  | *Hymeniacidon heliophila* | WBR | unknown | unknown | unknown | unknown | unknown | unknown | 3 |
|  | *Aplysina cavernicola* | WBR | archaeocytes, choanocytes | U, D | unknown | yes = archaeocytes, choanocytes | unknown | dedifferentiation | 4 |
|  | *Spongilla lacustris* | osculum | unknown | unknown | unknown | unknown | unknown | unknown | 5 |
| Calcarea | *Leucosolenia complicata* | WBR | choanocytes,  pinacocytes (?) | D | unknown | yes = choanocytes | thought to be pluripotent | transdifferentiation | 6,2 |
|  | *Leucosolenia cf. variabilis* | WBR | choanocytes,  pinacocytes | D | unknown | yes = choanocytes | thought to be pluripotent | transdifferentiation | 7 |
| Homoscleromorpha | *Oscarella lobularis* | WBR | choanocytes (?),  vacuolar cells, type 2- archaeocyte-like (?) | D | *piwi*, *vasa*, *pl10*, *boule*, *nanos*, *bruno*, *pumilio*, *tudor*, *ago* | yes = choanocytes? | unknown | transdifferentiation | 8, 9, 2 |
| Hexactinellida | *Aphrocallistes vastus* | WBR | unknown | unknown | unknown | unknown | unknown | unknown | 10 |
| **PLACOZOA** |  |  |  |  |  |  |  |  |  |
|  | *Trichoplax adhaerens* | WBR | unknown | unknown | unknown |  | unknown | unknown | 11 |
| **CNIDARIA** |  |  |  |  |  |  |  |  |  |
| Hydrozoa | *Hydra* spp. | WBR | i-cells | U | *nanos*, *piwi*, *myc*, *max* | yes = i-cells | multipotent for some  i-cell populations (not all) | unknown | 12, 13 |
|  | *Hydra* spp. | WBR | ectodermal stem cells | D | unknown | yes = ectodermal stem cells | multipotent | unknown | 14–16 |
|  | *Hydra* spp. | WBR | endodermal stem cells | D | unknown | yes = endodermal stem cells | multipotent | unknown | 14–16 |
|  | *Hydractinia echinata* | oral | i-cells | U | *vasa*, *piwi*, *nanos*, *pou* | yes = i-cells | thought to be pluripotent | unknown | 17, 13 |
|  | *Clytia hemisphaerica* | manubrium | i-cells | U | *piwi*, *nanos*, *vasa*, *pl10* | yes = i-cells | thought to be at least multipotent | unknown | 18, 19, 16 |
|  | *Podocoryne carnea* | manubrium | muscle cell? | D | unknown | unknown | unknown | transdifferentiation | 20 |
|  | *Turritopsis dohrnii* | manubrium/ umbrella | i-cells | U | unknown | yes = i-cells | multipotent | unknown | 21 |
| Scyphozoa | *Aurelia aurita* | WBR | unknown | unknown | unknown | unknown | unknown | transdifferentiation? | 22, 23 |
|  | *Cassiopea xamachana* | sensory organ | unknown | unknown | unknown | unknown | unknown | unknown | 24 |
| Cubozoa | *Tripedalia cystophora* | rhopalia (visual structure) | unknown | unknown | unknown | unknown | unknown | unknown | 25 |
| Staurozoa | *Haliclystus* sp. | peduncle, calyx | unknown | unknown | unknown | unknown | unknown | unknown | 26 |
| Hexacorallia | *Nematostella vectensis* | WBR | epithelial cells? | D | unknown | unknown | unknown | unknown | 27 |
|  | *Tubastraea coccinea* and  T. *tagusensis* | WBR | unknown | unknown | unknown | unknown | unknown | unknown | 28 |
|  | *Pocillopora damicornis* | WBR | unknown | unknown | unknown | unknown | unknown | unknown | 29 |
|  | *Acropora hyacinthus* | WBR | unknown | unknown | unknown | unknown | unknown | unknown | 29 |
| Octocorallia | *Gersemia rubiformis* | WBR | unknown | unknown | unknown | unknown | unknown | unknown | 30 |
| **CTENOPHORA** |  |  |  |  |  |  |  |  |  |
| Lobata | *Mnemiopsys leidyi* | WBR, comb plate | unknown | unknown | unknown | unknown | unknown | unknown | 31, 32, 33, 34 |
| Platyctenida | *Vallicula multiformis* | apical organ | unknown | unknown | unknown | unknown | unknown | unknown | 35 |
| Cydippida | *Pleurobrachia pileus* | comb plate | unknown | unknown | unknown | unknown | unknown | unknown | 32 |
| **ACOELOMORPHA** |  |  |  |  |  |  |  |  |  |
|  | *Hofstenia miamia* | WBR | neoblast | U | *piwi* | yes = neoblast | thought to be pluripotent | unknown | 36 |
|  | *Symsagittifera roscoffensis* | A and P | neoblast | U | unknown | yes = neoblast | thought to be pluripotent | unknown | 37, 38 |
|  | *Isodiametra pulchra* | P | neoblast | U | *piwi* | yes = neoblast | thought to be pluripotent | unknown | 39, 40 |
|  | *Convolutriloba retrogemma* | WBR | neoblast? | U? | unknown | yes = neoblast? | thought to be pluripotent | unknown | 41, 42 |
|  | *Convolutriloba longifissura* | WBR | neoblast | U | unknown | yes = neoblast? | thought to be pluripotent | unknown | 43 |
| **PLATYHELMINTHES** |  |  |  |  |  |  |  |  |  |
| Tricladida | *Schmidtea mediterranea* | WBR | c-neoblast | U | *vasa*, *piwi*, *tudor*,*nanos*, *bruno*, *smb*, *pumilio* | yes = neoblast | pluripotent | unknown | 44–47 |
|  | *Dugesia japonica* | WBR | neoblast | U | *vasa*, *piwi*, *nanos*, *pumilio*, *bruno*, *ago*, *tudor* | yes = neoblast | thought to be pluripotent | unknown | 48, 49 |
|  | *Dugesia tahitiensis* | WBR | neoblast | U | unknown | yes = neoblast? | unknown | unknown | 50 |
| Macrostopmorpha | *Macrostomum lignano* | A and P | neoblast | U | *vasa*, *piwi* | yes = neoblast | thought to be pluripotent | unknown | 51–53 |
|  | *Microstomum lineare* | A | neooblast | U? | unknown | yes = neoblast? | unknown | unknown | 54, 55 |
|  | *Stenostomum leucops* | A | neooblast | U | unknown | yes = neoblast? | unknown | unknown | 55 |
| **NEMERTEA** |  |  |  |  |  |  |  |  |  |
| Palaeonemertea | *Tubulanus ruber* | A and P | unknown | unknown | unknown | unknown | unknown | unknown | 56, 57 |
|  | *Cephalothrix spiralis* | P | unknown | unknown | unknown | unknown | unknown | unknown | 56, 57 |
| Hoplonemertea | *Prostoma graecense* | A and P | unknown | unknown | unknown | unknown | unknown | unknown | 56, 57 |
|  | *Prostoma cf. eilhardi* | P | unknown | unknown | unknown | unknown | unknown | unknown | 56, 57 |
| Pilidiophora | *Lineus sanguineus* | WBR | unknown | unknown | unknown | unknown | unknown | unknown | 56, 57 |
|  | *Lineus lacteus* | P | unknown | unknown | unknown | unknown | unknown | unknown | 56, 57 |
| **ANNELIDA** |  |  |  |  |  |  |  |  |  |
| Sedentaria | *Capitella teleta* | P | multipotent progenitor cell clusters / neoblast-like | U? | *vasa*, *piwi*, *nanos*, *myc* | yes = neoblast-like | thought to be multipotent | unknown | 58–60 |
|  | *Pristina leydi* | WBR | neoblast? (sliders) | U? | unknown | unknown | unknown | unknown | 61, 59, 62 |
|  | *Enchytraeus japonensis* | WBR | neoblast-like, N cells, epidermal and intestinal cells? | U, D | *vasa*, *piwi* | yes = neoblast-like | to be multipotent | dedifferentiation for endodermal and ectodermal tissues | 63, 64, 65, 59 |
|  | *Enchytraeus buchholzi* | A and P | unknown | D | unknown | unknown | unknown | dedifferentiation | 66 |
|  | *Eisenia fetida* | A and P | unknown | unknown | *sox* | unknown | unknown | unknown | 67, 68 |
|  | *Lumbriculus variegatus* | WBR | unknown | unknown | unknown | unknown | unknown | unknown | 69, 70 |
|  | *Aeolosoma viride* | A and P | unknown | unknown | unknown | unknown | unknown | unknown | 71 |
|  | *Pomatoceros lamarkii* | head appendage | unknown | unknown | unknown | unknown | unknown | unknown | 72 |
| Errantia | *Platynereis dumerilii* | P | unknown | D | *piwi*, *vasa*, *pl10*, *nanos*, *myc* | unknown | unknown | dedifferentiation? | 73, 74 |
|  | *Alitta virens* | P | unknown | unknown | *pl10*, *vasa*, *piwi* | unknown | unknown | unknown | 75, 76, 59 |
|  | *Typosyllis antoni* | P | unknown | unknown | unknown | unknown | unknown | unknown | 77 |
|  | *Syllis gracilis* | A and P | unknown | unknown | unknown | unknown | unknown | unknown | 78 |
|  | *Dorvillea bermudensis* | WBR | unknown | unknown | unknown | unknown | unknown | unknown | 79 |
| **BRACHIOPODA** |  |  |  |  |  |  |  |  |  |
|  | *Lingula anatina* | pedicle | unknown | unknown | unknown | unknown | unknown | unknown | 80 |
| **PHORONIDA** |  |  |  |  |  |  |  |  |  |
|  | *Phoronis psammophila* | A | unknown | unknown | unknown | unknown | unknown | unknown | 81 |
|  | *Phoronis vancouverensis* | WBR | unknown | unknown | unknown | unknown | unknown | unknown | 82 |
| **ECTOPROCTA** |  |  |  |  |  |  |  |  |  |
|  | *Cryptosula pallasiana* | WBR | unknown | unknown | unknown | unknown | unknown | unknown | 83 |
| **GASTROTRICHA** |  |  |  |  |  |  |  |  |  |
|  | *Turbanella* sp. | unclear | unknown | unknown | unknown | unknown | unknown | unknown | 84 |
| **MOLLUSCA** |  |  |  |  |  |  |  |  |  |
| Cephalopoda | *Sepia*, *Octopus* | appendage: arm | unknown | unknown | unknown | unknown | unknown | dedifferentiation | 85 |
|  | *Octopus vulgaris* | nerve | unknown | unknown | unknown | unknown | unknown | unknown | 86 |
|  | *Nautilus pompilius* | shell | unknown | unknown | unknown | unknown | unknown | unknown | 87 |
| Gastropoda | *Limax valentianus* | tentacle | unknown | unknown | unknown | unknown | unknown | unknown | 88, 56 |
|  | *Hydrobia ulvae* | head | unknown | unknown | unknown | unknown | unknown | unknown | 89 |
|  | *Melampus bidentatus* | nervous system | unknown | unknown | unknown | unknown | unknown | unknown | 90, 91 |
|  | *Melampus bidentatus* | penial complex | unknown | unknown | unknown | unknown | unknown | unknown | 92 |
|  | *Melampus bidentatus* | visual system | unknown | unknown | unknown | unknown | unknown | unknown | 93, 56 |
|  | *Aplysia californica* | axon | unknown | unknown | unknown | unknown | unknown | unknown | 94, 95 |
| Bivalvia | *Amiantis purpurata* | siphon | unknown | unknown | unknown | unknown | unknown | unknown | 96, 56 |
|  | *Hyriopsis cumingii* | mantle | unknown | unknown | unknown | unknown | unknown | unknown | 97, 56 |
| **ENTOPROCTA** |  |  |  |  |  |  |  |  |  |
|  | *Pedicellinidae Barentsiidae* | A | unknown | unknown | unknown | unknown | unknown | unknown | 98 |
|  | *Barentsia discreta* | WBR | unknown | unknown | unknown | unknown | unknown | unknown | 99 |
| **CHAETOGNATHA** |  |  |  |  |  |  |  |  |  |
|  | *Spadella cephaloptera* | P | unknown | unknown | unknown | unknown | unknown | unknown | 100 |
|  | *Sagitta* spp. | A | unknown | unknown | unknown | unknown | unknown | unknown | 100 |
| **ROTIFERA** |  |  |  |  |  |  |  |  |  |
|  | *Apsilus vorae* | unclear but reported | unknown | unknown | unknown | unknown | unknown | unknown | 101 |
| **ARTHROPODA** |  |  |  |  |  |  |  |  |  |
| Crustacea | *Parhyale hawaïensis* | Appendage: Leg | satellite-like cells | U | unknown | yes = satellite-like cells | unipotent | unknown | 102 |
|  | *Asellus aquaticus* | Appendage: Limb antennae | unknown | unknown | unknown | unknown | unknown | unknown | 103, 104 |
|  | *Uca pugilator* | Appendage: Limb | unknown | unknown | unknown | unknown | unknown | unknown | 105 |
|  | various species | eye | unknown | unknown | unknown | unknown | unknown | unknown | 106 |
|  | *Procambarus fallax* f. *virginalis* | Appendage: leg | unknown | unknown | unknown | unknown | unknown | unknown | 107 |
| **ONYCHOPHORA** |  |  |  |  |  |  |  |  |  |
| Whole taxon | _ | no WBR reported |  |  |  |  |  |  | 108 |
| **TARDIGRADA** |  |  |  |  |  |  |  |  |  |
| Whole taxon | _ | no WBR reported |  |  |  |  |  |  | 108 |
| **NEMATODA** |  |  |  |  |  |  |  |  |  |
| Whole taxon | *Enoplus communis* | P, but never reported again – unclear | unknown | unknown | unknown | unknown | unknown | unknown | 101 |
| **PRIAPULIDA** |  |  |  |  |  |  |  |  |  |
|  | _ | no WBR reported |  |  |  |  |  |  | 108 |
| **XENOTURBELLIDA** |  |  |  |  |  |  |  |  |  |
|  | *Xenoturbella bocki* | limited – unclear | unknown | unknown | unknown | unknown | unknown | unknown | 109 |
| **ECHINODERMATA** |  |  |  |  |  |  |  |  |  |
| Crinoidea | *Himerometra robustipinna* | gut | unknown | D | unknown | unknown | unknown | transdifferentiation | 110 |
|  | *Antedon mediterranea* | appendage: arm | amoebocytes? coelomocytes? | U | unknown | yes = amoebocytes/ coelomocytes? | thought to be multipotent | unknown | 111–113 |
| Asteroidea | *Asterias rubens*, *Leptasterias hexactis*, *Echinaster sepositus*, *Linckia guildinguii* | WBR /  appendage: arm | coelomic epithelium/  coelomocytes? | D/U | unknown | unknown | unknown | dedifferentiation | 114, 115 |
|  | *Allostichaster capensis* | appendage: arm | unknown | unknown | unknown | unknown | unknown | unknown | 116 |
|  | *Coscinasterias tenuispina* | appendage: arm | unknown | unknown | unknown | unknown | unknown | unknown | 117 |
| Ophiuroidea | *Amphiura filiformis* | WBR /  appendage: arm | unknown | unknown | unknown | unknown | unknown | unknown | 118–120 |
| Holothuroidea | *Eupentacta fraudatrix* | gut | coelomic epithelium (mesothelium) | D | *myc* | yes = coelomic epithelial cells | unknown | dedifferentiation? | 111, 121–125 |
|  | *Holothuria glaberrima* | body wall | unknown | D | unknown | unknown | unknown | unknown | 126 |
|  | *Holothuria glaberrima* | radial nerve cord | radial glial cells | D | *sox* | yes= radial glial cells | thought to be multipotent | dedifferentiation/  transdiffferentiation | 127, 124 |
| Echinoidea | *Lytechinus variegatus* | appendage: tube feet | unknown | D | *vasa*, *piwi* | unknown | unknown | unknown | 128 |
| **HEMICHORDATA** |  |  |  |  |  |  |  |  |  |
| Enteropneusta | *Ptychodera flava* | A and P | mesenchymal cells? | D? | *Sox* | unknown | unknown | dedifferentiation? | 129–131 |
|  | *Balanoglossus simodensis* | WBR | unknown | unknown | unknown | unknown | unknown | unknown | 132 |
| Pterobranchia | _ | unknown | _ | _ | _ | unknown | _ | _ | 133 |
| **UROCHORDATA** |  |  |  |  |  |  |  |  |  |
| Stolidobranchia | *Botryllus schlosseri / Botryllus primigenus* | WBR | haemocytes, epithelial cells? | U/D | *piwi*, *vasa*, *pl10*, *pitx*, *pou* | yes = haemocytes | thought to be multipotent | unknown | 134–137 |
|  | *Botrylloides violaceus /Botrylloides leachii* | WBR | haemocytes, epithelial cells? | U/D | *piwi*, *vasa* | yes = haemocytes | thought to be multipotent | unknown | 134–140 |
|  | *Botrylloides diegensis* | WBR | haemocytes | U | *Integrin alpha 6* | yes = haemocytes | thought to be multipotent | unknown | 141 |
|  | *Polyandrocarpa mysakiensis* | WBR | haemocytes, epithelial cells? | U/D | *myc* | yes = haemocytes | thought to be multipotent | unknown | 134–136, 142 |
|  | *Polyandrocarpa zorritiensis* | WBR | haemocytes, epithelial cells? | U/D | unknown | yes = haemocytes | unknown | unknown | 143 |
| Phlebobranchia | *Ciona robusta* | oral siphon tip | siphon stem cells | unknown | *piwi* | yes = siphon stem cells | unknown | unknown | 144 |
|  | *Ciona robusta* | oral siphon tube and base | branchial sac stem cells, branchial sac progenitor cells | U? | *piwi* | yes = haemoblast from branchial sac | multipotent | unknown | 145 |
|  | *Perophora viridis* | WBR | circulating progenitor cells | U | unknown | yes = circulating progenitor cells | pluripotent | unknown | 146 |
| Thaliacea | *Thetys vagina* | unclear | unknown | unknown | unknown | unknown | unknown | unknown | 147 |
| Aplousobranchia | *Rhopalaea idoneta* | thorax | epicardium stem cells | unknown | unknown | unknown | unknown | unknown | 148 |
| **CEPHALOCHORDATA** |  |  |  |  |  |  |  |  |  |
| Leptocardii/  Amphioxiformes | *Branchiostoma lanceolatum* | A and P | muscle satellite-like cells, neural cells, notochord cells | U (and D?) | *Pax3/7*, *sox* | yes = muscle satellite-like progenitor | unknown | dedifferentiation? | 148–151 |
|  | *Branchiostoma japonicum* | A and P | unknown | unknown | unknown | unknown | unknown | unknown | 152, 153 |
|  | *Branchiostoma belcheri* | P | unknown | unknown | unknown | unknown | unknown | unknown | 152 |
|  | *Branchiostoma belcheri* | oral cirri | unknown | unknown | *sox* | unknown | unknown | unknown | 154 |
|  | *Branchiostoma platae* | P | unknown | unknown | unknown | unknown | unknown | unknown | 155, 156 |
|  | *Asymmetron lucayanum* | P | unknown | unknown | unknown | unknown | unknown | unknown | 157 |
|  | *Epigonichthys* | not reported | _ | _ | _ | _ | _ | _ | 150 |
| **VERTEBRATA** |  |  |  |  |  |  |  |  |  |
|  | *Danio rerio* | appendage: fin | osteoblast | D | *myc*, *sox?* | unknown | unipotent | dedifferentiation | 158, 159 |
|  | *Danio rerio* | appendage: fin | mesenchymal cells | D | *myc*, *sox?* | unknown | unipotent | dedifferentiation | 158, 159 |
|  | *Danio rerio* | heart | cardiomyocytes | D | unknown | unknown | unipotent | dedifferentiation | 159 |
|  | *Danio rerio* | liver | hepatocytes | D | unknown | no | _ | proliferation of pre-existing hepatocytes | 159 |
|  | *Danio rerio* | pancreas | α- and β- pancreatic cells | D | unknown | no | _ | transdifferentiation | 159 |
|  | *Danio rerio* | kidney | adult nephron progenitor cells | U | unknown | yes = adult nephron progenitor cells | unipotent | unknown | 159 |
|  | *Danio rerio* | skeletal muscle | muscle satellite cells | U | *pax7* | yes = muscle satellite cells | unknown | unknown | 159 |
|  | *Danio rerio* | neural tissues | radial glia neural stem cells | D | unknown | yes = radial glia neural stem cells | unknown | unknown | 159 |
|  | *Notophthalmus viridescens* | appendage: limb | muscle cells | D | *klf*, *sox*, *myc* | unknown | unipotent | dedifferentiation | 160–163 |
|  | *Ambystoma mexicanum* | appendage: limb | muscle satellite cells | U | *piwi* | yes = muscle satellite cells | unipotent | unknown | 161–163 |
|  | *Ambystoma mexicanum* | appendage: limb | connective tissue (CT) lineages / multipotent skeletal progenitor | U/D | *sox* | yes = multipotent skeletal progenitor | multipotent | dedifferentiation | 161, 164 |
|  | *Ambystoma* spp. | lens | pigmented epithelial cells | D | *klf*, *sox*, *myc* | unknown | unipotent | dedifferentiation | 160 |
|  | *Ambystoma* spp. | brain | ependymoglial cells | D | unknown | unknown | unipotent | dedifferentiation | 165 |

**References**

1. Borisenko IE, Adamska M, Tokina DB, Ereskovsky AV. 2015. Transdifferentiation is a driving force of regeneration in *Halisarca dujardini* (Demospongiae, Porifera). PeerJ. 3:e1211.
2. Funayama N. 2018. The cellular and molecular bases of the sponge stem cell systems underlying reproduction, homeostasis and regeneration. Int J Dev Biol. 62:513-525.
3. Coutinho CC, Rosa IDA, Teixeira JDDO, Andrade LR, Costa ML, Mermelstein C. 2017. Cellular migration, transition and interaction during regeneration of the sponge *Hymeniacidon heliophila*. PLoS ONE. 12(5):e0178350.
4. Ereskovsky AV, Tokina DB, Saidov DM, Baghdiguian S, Le Goff E, Lavrov AI. 2020. Transdifferentiation and mesenchymal‐to‐epithelial transition during regeneration in Demospongiae (Porifera). J Exp Zool B Mol Dev Evol. 334(1):37-58.
5. Windsor Reid PJ, Matveev E, McClymont A, Posfai D, Hill, AL, Leys SP. 2018. Wnt signaling and polarity in freshwater sponges. BMC Evol Biol. 18:12.
6. Ereskovsky A, Lavrov A, Bolshakov F, Tokina D. 2017*b*. Regeneration in White Sea sponge *Leucosolenia complicata* (Porifera, Calcarea). Inv Zool. 14(2):108-113.
7. Lavrov AI, Bolshakov FV, Tokina DB, Ereskovksy AV. 2018. Sewing up the wounds: The epithelial morphogenesis as a central mechanism of calcaronean sponge regeneration. J Exp Zool B Mol Dev Evol. 330(6-7): 351-371.
8. Ereskovsky AV, Borisenko IE, Lapebie P, Gazave E, Tokina DB, Borchiellini C. 2015. *Oscarella lobularis* (Homoscleromorpha) ectosome regeneration: Epithelial morphogenesis and metaplasie. PLoS ONE 10(8): e0134566.
9. Fierro-Constaín L, Schenkelaars Q, Gazave E, Haguenauer A, Rocher C, Ereskovsky E, Borchiellini C, Renard E. 2017. The conservation of the germline multipotency program, from sponges to vertebrates: A stepping stone to understanding the somatic and germline origins. Genome Biol Evol. 9(3):474-488.
10. Leys SP, Mackie GO, Reiswig HM. 2007. The biology of glass sponges. Adv Mar Biol. 52:1-145.
11. Syed T, Schierwater B. 2002. *Trichoplax adhaerens*: discovered as a missing link, forgotten as a hydrozoan, re-discovered as a key to metazoan evolution. Vie Milieu. 52:177-187.
12. Khalturin K, Anton-Erxeleben F, Milde S, Plötz C, Wittlieb J, Hemmrich G, Bosch TCG. 2007. Transgenic stem cells in *Hydra* reveal an early evolutionary origin for key elements controlling self-renewal and differentiation. Dev Biol. 309:32-44.
13. Gahan JM, Bradshaw B, Glici H, Frank U. 2016. The interstitial stem cells in *Hydractinia* and their role in regeneration. Curr Opin Genet Dev.40:65-73.
14. Hemmrich G, Khalturin K, Boehm A-M, Puchert M, Anton-Erxleben F, Wittlieb J, Klostermeier UC, Rosenstiel P, Oberg H-H, Domazet-Loso T, Sugimoto T, Niwa H, Bosch TCG. 2012. Molecular signatures of the three stem cell lineages in *Hydra* and the emergence of stem cell function at the base of multicellularity. Mol Biol Evol. 29:3267-3280.
15. Petersen HO, Hoger SK, Looso M, Lengfeld T, Kuhn A, Warnken U, Nishimiya-Fujisawa C, Schnolzer M, Kruger M, Ozbek S, Simakov O, Holstein, TW. 2015. A comprehensive transcriptomic and proteomic analysis of *Hydra* head regeneration. Mol Biol Evol. 32:1928-1947.
16. Leclère L, Copley RR, Momose T, Houliston E. 2016. Hydrozoan insights in animal development and evolution. Curr Opinion Genet Dev. 39:157-67.
17. Bradshaw B, Thompson K, Frank U. 2015. Distinct mechanisms underlie oral vs aboral regeneration in the cnidarian *Hydractinia echinata*. eLife. 4:e05506.
18. Denker E, Manuel M, Leclère L, Le Guyader H, Rabet N. 2008. Ordered progression of nematogenesis from stem cells through differentiation stages in the tentacle bulb of *Clytia hemisphaerica* (Hydrozoa Cnidaria). Dev Biol. 315:99-113.
19. Leclère L, Jager M, Barreau C, Chang P, Le Guyader H, Manuel M, Houliston E. 2012. Maternally localized germ plasm mRNAs and germ cell/stem cell formation in the cnidarian *Clytia*. Dev Biol. 364:236-248.
20. Schmid V, Wydler M, Alder H. 1982. Transdifferentiation and regeneration in vitro. Dev Biol. 92:476-488.
21. Tardent P. 1963. Regeneration in the Hydrozoa. Biol Rev Camb Philos Soc. 38:293-333.
22. Abrams MJ, Basinger T, Yuan W, Guo CL, Goentoro L. 2015. Self-repairing symmetry in jellyfish through mechanically driven reorganization. Proc Natl Acad Sci USA. 112:E3365-E3373.
23. Nakanishi N, Jacobs DK. 2019. The early evolution of cellular reprogramming in animals. In Bishop CD, Hall, BK editors. Deferring development. 1^st^ ed. New York: CRC press. p. 67-86.
24. Stockard CR. 1908. Studies of tissue growth. I. On experimental study of the rate of regeneration in *Cassiopea xamachana*. Pap from Tortugas Lab Carnegie Inst Wash. 2:61-102.
25. Stamatakis SA, Worsaae K, Garm A. 2018. Regeneration of the rhopalium and the rhopalial nervous system in the box jellyfish *Tripedalia cystophora*. Biol Bull. 234:22-36.
26. Meyer A. 1865. Über die Reproduktionskraft der Lucernarien. Amtl Ber 40 Verslag Deutsch Naturf Ärzte. Hannover. 1865:217.
27. Amiel AR, Johnston HT, Nedoncelle K, Warner JF, Ferreira S, Röttinger E. 2015. Characterization of morphological and cellular events underlying oral regeneration in the sea anemone, *Nematostella vectensis*. Int J Mol Sci. 16(12):28449-71.
28. Luz BLP, Capel KCC, Zilberberg C, Flores AAV, Migotto AE, Kitahara MV. 2018. A polyp from nothing: the extreme regeneration capacity of the Atlantic invasive sun corals *Tubastraea coccinea* and *T. tagusensis* (Anthozoa, Scleractinia). J Exp Mar Biol Ecol. 503:60-65.
29. Traylor-Knowles N. 2016. Distinctive wound-healing characteristics in the corals *Pocillopora damicornis* and *Acropora hyacinthus* found in two different temperature regimes. Mar Biol. 163(11):231.
30. Henry LA, Kenchington EL, Silvaggio A. 2003. Effects of mechanical experimental disturbance on aspects of colony responses, reproduction and regeneration in the cold water octocoral *Gersemia rubiformis*. Can J Zool. 81(10):1691-1701.
31. Henry JQ, Martindale MQ. 2000. Regulation and regeneration in the ctenophore *Mnemiopsis leidyi*. Dev Biol. 227:720-733.
32. Tamm SL. 2012. Regeneration of ciliary comb plates in the ctenophore *Mnemiopsis leidyi*. J Morphol. 273:109-120.
33. Martindale MQ. 2016. The onset of regenerative properties in ctenophores. Curr Opin Genet Dev. 40:113-119.
34. Ramon-Mateu J, Tori Ellison S, Angelini TE, Martindale MQ. 2019. Regeneration in the ctenophore *Mnemiopsis leidyi* occurs in the absence of blastema, requires cell division, and is temporally separable from wound healing. BMC Biol. 17:80.
35. Freeman G. 1967. Studies on regeneration in the creeping ctenophore, *Vallicul multiformis*. J Morphol. 123:71-84.
36. Gehrke AR, Neverett E, Luo YJ, Brandt A, Ricci L, Hulett RE, Gompers A, Ruby JG, Rokhsar DS, Reddien PW, Srivastava M. 2019. Acoel genome reveals the regulatory landscape of whole-body regeneration. Science. 363:6432.
37. Sprecher SG, Bernardo-Garcia FJ, van Giesen L, Hartenstein V, Reichert H, Neves R, Bailly X, Martinez P, Brauchle M. 2015. Functional brain regeneration in the acoel worm *Symsagittifera* *roscoffensis*. Biol Open. 4(12):1688-95.
38. Arboleda E, Hartenstein V, Martinez P, Reichert H, Sen S, Sprecher S, Bailly X. 2018. An emerging system to study photosymbiosis, brain regeneration, chronobiology, and behavior: The marine acoel *Symsagittifera roscoffensis*. Bioessays 40(10):e1800107.
39. De Mulder K, Kuales G, Pfister D, Willems M, Egger B, Salvenmoser W, Thaler M, Gorny AK, Hrouda M, Borgonie G, Ladurner P. 2009*a*. Characterization of the stem cell system of the acoel *Isodiametra pulchra*. BMC Dev Biol. 9:69.
40. Perea-Atienza E, Botta M, Salvenmoser W, Gschwentner R, Egger B, Kristof A, Martinez P, Achatz JG. 2013. Posterior regeneration in *Isodiametra pulchra* (Acoela, Acoelomorpha). Front Zool. 10(1):64.
41. Sikes JM, Bely AE. 2010. Making heads from tails: development of a reversed anterior-posterior axis during budding in an acoel. Dev Biol. 338(1):86-97.
42. Achatz JG, Chiodin M, Salvenmoser W, Tyler S, Martinez P. 2013. The Acoela: on their kind and kinships, especially with nemertodermatids and xenoturbellids (Bilateria incertae sedis). Org Divers Evol. 13(2):267-286.
43. Gschwentner R, Ladurner P, Nimeth K, Rieger R. 2001. Stem cells in a basal bilaterian-S-phase and mitotic cells in *Convolutriloba longifissura* (Acoela, Platyhelminthes). Cell Tissue Res. 304:401-408.
44. Reddien PW, Oviedo NJ, Jennings JR, Jenkin JC, Sánchez Alvarado A. 2005. SMEDWI-2 is a PIWI-like protein that regulates planarian stem cells. Science. 310:1327-1330.
45. Handberg-Thorsager M, Saló E. 2007. The planarian nanos-like gene Smednos is expressed in germline and eye precursor cells during development and regeneration. Dev Genes Evol. 217:403-411.
46. Wagner DE, Ho JJ, Reddien PW. 2012. Genetic regulators of a pluripotent adult stem cell system in planarians identified by RNAi and clonal analysis. Cell Stem Cell. 10:299-311.
47. Wagner DE, Wang IE, Reddien PW. 2011. Clonogenic neoblasts are pluripotent adult stem cells that underlie planarian regeneration. Science 332:811-816.
48. Rouhana L, Shibata N, Nishimura O, Agata K. 2010. Different requirements for conserved post-transcriptional regulators in planarian regeneration and stem cell maintenance. Dev Biol. 341:429-443.
49. Shibata N, Hayashi T, Fukumura R, Fujii J, Kudome-Takamatsu T, Nishimura O, Sano S, Son F, Suzuki N, Araki R, Abe M, Agata K. 2012. Comprehensive gene expression analyses in pluripotent stem cells of a planarian, *Dugesia japonica.* Int J Dev Biol. 56:93-102.
50. Peter R, Ladurner P, Rieger RM. 2001. The role of stem cell strategies in coping with environmental stress and choosing between alternative reproductive modes: Turbellaria rely on a single cell type to maintain individual life and propagate species. Mar Ecol. 22:35-51.
51. Egger B, Gschwentner R, Hess MW, Nimeth KT, Adamski Z, Willems M, Rieger R, Salvenmose W. 2009*a*. The caudal regeneration blastema is an accumulation of rapidly proliferating stem cells in the flatworm *Macrostomum lignano*. BMC Dev Biol. 9:41.
52. Grudniewska M, Mouton S, Simanov D, Beltman F, Grelling M, De Mulder K, Arindrarto W, Weissert PM, Van Der Elst S, Berezikov E. 2016. Transcriptional signatures of somatic neoblasts and germline cells in *Macrostomum lignano*. eLife 5: e20607.
53. Mouton S, Wudarski J, Grudniewska M, Berezikov E. 2018. The regenerative flatworm *Macrostomum lignano*, a model organism with high experimental potential. Int J Dev Biol. 62:551–8.
54. Palmberg I. 1986. Cell migration and differentiation during wound healing and regeneration in *Microstomum lineare* (Turbellaria) Hydrobiologia. 132:181-188.
55. Palmberg I. 1990. Stem cells in microturbellarians – an autoradiographic and immunocytochemical study. Protoplasma. 158:109-120.
56. Bely AE, Zattara EE, Sikes JM. 2014. Regeneration in spiralians: evolutionary patterns and developmental processes. Int J Dev Biol 58:623-634.
57. Zattara EE, Fernández-Álvarez FÁ, Hiebert TC, Bely AE, Norenburg JL. 2019. A phylum-wide survey reveals multiple independent gains of head regeneration in Nemertea. Proc R Soc B. 286:20182524.
58. Giani VCJr, Yamaguchi E, Boyle MJ, Seaver EC. 2011. Somatic and germline expression of piwi during development and regeneration in the marine polychaete annelid *Capitella teleta*. EvoDevo. 2:10.
59. Özpolat BD, Bely AE. 2016. Developmental and molecular biology of annelid regeneration: a comparative review of recent studies. Curr Opin Genet Dev. 40:144-153.
60. de Jong DM, Seaver EC. 2017. Investigation into the cellular origins of posterior regeneration in the annelid *Capitella teleta*. Regeneration. 5:61-77.
61. Özpolat BD, Bely AE. 2015. Gonad establishment during asexual reproduction in the annelid *Pristina leidyi*. Dev Biol. 405:123-136.
62. Zattara EE, Turlington KW, Bely AE. 2016. Long-term time-lapse live imaging reveals extensive cell migration during annelid regeneration. BMC Dev Biol. 16:6.
63. Tadokoro R, Sugio M, Kutsuna J, Tochinai S, Takahashi Y. 2006. Early segregation of germ and somatic lineages during gonadal regeneration in the annelid *Enchytraeus japonensis*. Curr Biol. 16:1012-1017.
64. Sugio M, Takeuchi K, Kutsuna J, Tadokoro R, Takahashi Y, Yoshida-Noro C, Tochinai S. 2008. Exploration of embryonic origins of germline stem cells and neoblasts in *Enchytraeus japonensis* (Oligochaeta, Annelida). Gene Expr Patterns. 8:227-236.
65. Sugio M, Yoshida-Noro C, Ozawa K, Tochinai S. 2012. Stem cells in asexual reproduction of *Enchytraeus japonensis* (Oligochaeta, Annelid): proliferation and migration of neoblasts. Dev Growth Differ. 54:439-450.
66. Myohara M. 2012. What role do annelid neoblasts play? A comparison of the regeneration patterns in a neoblast-bearing and a neoblast-lacking enchytraeid oligochaete. PLoS ONE. 7(5):e37319.
67. Nengwen X, Ge F, Edwards CA. 2011. The regeneration capacity of an earthworm, *Eisenia fetida*, in relation to the site of amputation along the body. Acta Ecol Sin. 31:197-204.
68. Bhambri A, Dhaunta N, Patel SS, Hardikar M, Bhatt A, Srikakulam N, Shridhar S, Vellarikkal S, Pandey R, Jayarajan R, et al. 2018. Large scale changes in the transcriptome of *Eisenia fetida* during regeneration. PLoS ONE 13(9): e0204234.
69. Tweeten KA, Anderson A. 2008. Analysis of cell proliferation and migration during regeneration in *Lumbriculus variegatus* (Clitellata: Lumbriculidae). BIOS 79:183-190.
70. Tweeten KA, Reiner A. 2012. Characterization of serine proteases of *Lumbriculus variegatus* and their role in regeneration. Invert Biol. 131:322-332.
71. Chen CP, Ke-Wing Fok S, Hsieh YW, Chen CY, Hsu FP, Chang YH, Chen J-H. 2020. General characterization of regeneration in *Aeolosoma viride* (Annelida, Aeolosomatidae). Invert Biol. 139:e12277.
72. Szabó R, Ferrier DEK. 2014. The dynamics of alkaline phosphatase activity during operculum regeneration in the polychaete *Pomatoceros lamarckii*. Int J Dev Biol. 58:635-642.
73. Pfeifer K, Dorresteijn AWC, Fröbius AC. 2012. Activation of Hox genes during caudal regeneration of the polychaete annelid *Platynereis dumerilii*. Dev Genes Evol. 222:165-179.
74. Planques A, Malem J, Parapar J, Vervoort M, Gazave E. 2019. Posterior regeneration in the annelid *Platynereis*. Dev Biol. 445(2):189-210.
75. Kozin VV, Kostyuchenko RP. 2015. Vasa, PL10, and Piwi gene expression during caudal regeneration of the polychaete annelid *Alitta virens*. Dev Genes Evol. 225(3):129-138.
76. Kozin VV, Filippova NA, Kostyuchenko RP. 2017. Regeneration of the nervous and muscular system after caudal Amputation in the Polychaete *Alitta virens* (Annelida: nereididae). Russ J Dev Biol. 48:198-210.
77. Ribeiro RP, Bleidorn C, Aguado MT. 2018. Regeneration mechanisms in Syllidae (Annelida). Regeneration. 5(1):26-42.
78. Ribeiro RP, Ponz-Segrelles G, Bleidorn C, Aguado MT. 2018b. Comparative transcriptomics in Syllidae (Annelida) indicates that posterior regeneration and regular growth are comparable, while anterior regeneration is a distinctive process. BMC Genomics. 20: 855.
79. Paulus T, Müller M. 2006. Cell proliferation dynamics and morphological differentiation during regeneration in *Dorvillea bermudensis* (Polychaeta, Dorvilleidae). J Morphol. 267:393-403.
80. Hammond LS. 1983. Experimental studies of salinity tolerance, burrowing behavior and pedicle regeneration in *Lingula anatina* (Brachiopoda, Inarticulata). J Paleontol. 57(6):1311-1316.
81. Emig CC. 1972. Régénération de la région antérieure chez *Phoronis psammophila* Cori (Phoronida). Z Morph Tiere. 73(2):117-144.
82. Marsden JR. 1957. Regeneration in *Phoronis vancouverensis.* J Morphol. 101:307-323.
83. Lombardi C, Taylor PD, Cocito S, Bertolini C, Calosi P. 2017. Low pH conditions impair module capacity to regenerate in a calcified colonial invertebrate, the bryozoan *Cryptosula pallasiana*. Mar Env Res. 125:110-117
84. Manylov OG. 1995. Regeneration in Gastrotricha – I. Light microscopical observations on the regeneration in *Turbanella* sp. Acta Zool, 76:1-6.
85. Imperadore P, Fiorito G. 2018. Cephalopod tissue regeneration: consolidating over a century of knowledge. Front Physiol. 9:593.
86. Imperadore P, Uckermann O, Galli R, Steiner G, Kirsch M, Fiorito G. 2018. Nerve regeneration in the cephalopod mollusc *Octopus vulgaris*: label-free multiphoton microscopy as a tool for investigation. J R Soc Inter. 15:20170889.
87. Yomogida S, Wani, R. (2013). Higher risk of fatality by predatory attacks in earlier ontogenetic stages of modern *Nautilus pompilius* in the Philippines: evidence from the ontogenetic analyses of shell repairs. Lethaia 46:317-330.
88. Matsuo R, Kobayashi S, Tanaka Y, Ito E. 2010. Effects of tentacle amputation and regeneration on the morphology and activity of the olfactory center of the terrestrial slug *Limax* *valentianu*s. J Exp Biol 213:3144-3149.
89. Gorbushin AM, Levakin IA, Panchina NA, Panchin YV. 2001. *Hydrobia ulvae* (Gastropoda: Prosobranchia): A new model for regeneration studies. J Exp Biol 204: 283-289.
90. Moffett S, Austin DR. 1982. Generation of new cerebral ganglion neurons in the snail *Melampus*: An ultrastructural study. J Comp Neurol. 207:177-182.
91. Moffett S, Ridgway RL. 1988. Structural repair and functional recovery following cerebral ganglion removal in the pulmonate snail *Melampus*. Am Zool. 28:1109-1122.
92. Moffett SB. 1992. Mating behavior in the pulmonate snail *Melampus*: can regeneration restore function? Acta Biol Hung. 43(1-4):367-74.
93. Tuchina O, Meyer-Rochow VB. 2010. Regeneration of the visual system in gastropods (Mollusca). Invert Biol. 129: 27-38.
94. Moffett SB. 1996. Nervous system regeneration in the invertebrates. New York (NY): Springer.
95. Sànchez JA, Li Y, Kirk MD. 2000. Regeneration of cerebral-buccal interneurons and recovery of ingestion buccal motor programs in *Aplysia* after CNS lesions. J Neurophysiol. 84, 2961-2974.
96. Liu Y, Bai Z, Li Q, Zhao Y, Li J. 2013. Healing and regeneration of the freshwater pearl mussel *Hyriopsis cumingii* Lea after donating mantle saibos. Aquaculture 392-395: 34-43.
97. Nunez JD, Ocampo EH, Chiaradia NM, Morsan E, Cledon M. 2013. The effect of temperature on the inhalant siphon regeneration of *Amiantis purpurata* (Lamarck, 1818) (Bivalvia; Veneridae) Mar Biol Res. 9:189-197.
98. Merkel J, Wanninger A, Lieb B. 2018. Novel and conserved features of the Hox cluster of Entoprocta (Kamptozoa). J Phylogenetics Evol Biol. 6:1.
99. Mukai H, Makioka T. 1978. Studies on the regeneration of an entoproct, *Barentsia discreta*. J Exp Zool. 205:261-275.
100. Alvarino A. 1983. Chaetognatha. In: Adiyodi KG, Adiyodi RG, editors. Reproductive Biology of Invertebrates. New York (NY): John Wiley & Sons. Vol. 1, Oogenesis, Oviposition, and Oosorption. p. 585-610.
101. Vorontsova MA, Liosner LD. 1960. Asexual propagation and regeneration. Meryl Rose, S, translator. Chicago, Pergamon Press. p 488.
102. Konstantinides N, Averof M. 2014. A common cellular basis for muscle regeneration in arthropods and vertebrates. Science. 343(6172):788-91.
103. Needham AE. 1945. Peripheral nerve and regeneration in Crustacea. J of Exp Biol. 21:144-146.
104. Maruzzo D, Egredzija M, Minelli A, Fusco G. 2008. Segmental pattern formation following amputation in the flagellum of the second antennae of *Asellus aquaticus* (Crustacea, Isopoda), Ital J Zool. 75(3):225-231.
105. Khan SJ, Schuster KJ, Smith-Bolton RK. 2016. Regeneration in crustaceans and insects. In: eLS. John Wiley & Sons, Editors. Chichester. p. 1-14.
106. Steele MI. 1907. Regeneration in compound eyes of Crustacea. J Exp Zool. 5:163-243.
107. Shinji J, Miyanishi H, Gotoh H, Kaneko T. 2016. Appendage regeneration after autotomy is mediated by *baboon* in the crayfish *Procambarus Fallax* F. *Virginalis* Martin, Dorn, Kawai, Heiden and Scholtz, 2010 (Decapoda: Astacoidea: Cambaridae). J Crust Biol. 36(5):649-657.
108. Bely AE, Nyberg KG. 2010. Evolution of animal regeneration: re-emergence of a field. Trends Ecol. Evol. 25:161-170.
109. Nakano H. 2015. What is Xenoturbella? Zoological Letters. 1:22.
110. Kalacheva NV, Eliseikina MG, Frolova LT, Dolmatov IY. 2017. Regeneration of the digestive system in the crinoid *Himerometra robustipinna* occurs by transdifferentiation of neurosecretory-like cells. PLoS ONE. 12(7):e0182001.
111. Kondo M, Akasaka K. 2010. Regeneration in crinoids. Dev Growth Differ. 52(1):57-68.
112. Candia Carnevali MD, Bonasoro F, Lucca E, Thorndyke MC. 1995. Pattern of cell proliferation in the early stages of arm regeneration in the feather star *Antedon mediterranea*. J Exp Zool. 272:464-474.
113. Candia Carnevali MD, Bonasoro F, Biale A. 1997. Pattern of bromodeoxyuridine incorporation in the advanced stages of arm regeneration in the feather star *Antedon mediterranea*. Cell Tissue Res. 289:363-374.
114. Cortes Rivera Y, Hernandez I, Angel P, Meza E, Cuervo R. 2016. Regenerative potential of the sea star *Linckia guildinguii*. Hidrobiológica: 26:103-108.
115. Ben Khadra Y, Sugni M, Ferrario C, Bonasoro F, Varela Coelho A, Martinez P, Candia Carnevali MD. 2017. An integrated view of asteroid regeneration: tissues, cells and molecules. Cell Tissue Res. 370(1):13-28.
116. Rubliar T, Merett PE, Cledon M. 2015. Regeneration rate after fission in the fissiparous sea star *Allostichaster capensis* (Asteroidea). Rev Biol Trop. 63(2):321-8.
117. Garcia-Cisneros A, Perez-Portela R, Almroth BC, Degerman S, Palacin C, Nilsson Sköld H. 2015. Long telomeres are associated with clonality in wild populations of the fissiparous starfish *Coscinasterias tenuispina*. Heredity. 115(5): 437-443.
118. Biressi A, Ting Z, Dupont S, Dahlberg C, Di Benedetto C, Bonasoro F, Thorndyke M, Candia Carnevali MD. 2010. Wound-healing and arm regeneration in *Ophioderma longicaudum* and *Amphiura filiformis* (Ophiuroidea, Echinodermata): comparative morphogenesis and histogenesis. Zoomorph. 129:1-19.
119. Ben Khadra Y, Sugni M, Ferrario C, Bonasoro F, Oliveri P, Martinez P, Candia Carnevali MD. 2018. Regeneration in stellate echinoderms: Crinoidea, Asteroidea, and Ophiuroidea. Results Probl Cell Differ. 65:285-320.
120. Burns G, Ortega-Martinez O, Thorndyke M, Peck LS. 2012. Dynamic gene expression profiles during arm regeneration in the brittle star Amphiura filiformis. J Exp Mar Biol Ecol. 407(2):315-322.
121. Mashanov VS, García-Arrarás JE. 2011. Gut regeneration in holothurians: a snapshot of recent developments. Biol Bull. 221:93-109.
122. García-Arrarás JE, Lázaro-Peña MI, Díaz-Balzac CA. 2018. Holothurians as a model system to study regeneration. Results Probl Cell Differ. 65:255-283.
123. Mashanov V, Zueva O, García-Arrarás JE. 2015a. Expression of pluripotency factors in echinoderm regeneration. Cell Tissue Res. 359:521-536.
124. Mashanov V, Zueva O, García-Arrarás JE. 2015b. Myc regulates programmed cell death and radial glia dedifferentiation after neural injury in an echinoderm. BMC Dev Biol. 15:24.
125. Mashanov V, Zueva O, Mashanova D, García-Arrarás JE. 2017. Expression of stem cell factors in the adult sea cucumber digestive tube. Cell Tissue Res. 370(3):427-440.
126. San Miguel-Ruiz J, García-Arrarás J. 2007. Common cellular events occur during wound healing and organ regeneration in the sea cucumber *Holothuria glaberrima*. BMC Dev Biol. 7(1):1-19.
127. Mashanov VS, Zueva OR, García-Arrarás JE. 2014. Transcriptomic changes during regeneration of the central nervous system in an echinoderm. BMC Genomics. 15:357.
128. Reinardy HC, Emerson CE, Manley JM, Bodnar AG. 2015. Tissue regeneration and biomineralization in sea urchins: role of Notch signaling and presence of stem cell markers. PLoS One 10(8):e0133860.
129. Humphreys T, Sasaki A, Uenishi G, Tamparra K, Arimoto A, Tagawa K. 2010. Regeneration in the Hemichordate *Ptychodera flava*. Zool Sci. 27:91-5.
130. Arimoto A, Tagawa K. 2018. Regeneration in the enteropneust hemichordate, *Ptychodera flava*, and its evolutionary implications. Dev Growth Differ. 60(6):400-408.
131. Yoshimura K, Morino Y, Wada H. 2019. Regeneration of the acorn worm pygochord with the implication for its convergent evolution with the notochord. Dev Growth Differ. 61(2):158-165.
132. Miyamoto N, Saito Y. 2010. Morphological characterization of the asexual reproduction in the acorn worm *Balanoglossus simodensis*. Dev Growth Differ. 52: 615–627.
133. Rychel A, Swalla B. 2009. Regeneration in hemichordates and echinoderms. In: Rinkevich B, Matranga V, editors. Stem Cells in Marine Organisms. Dordrecht, Springer. p. 245-265.
134. Kürn U, Rendulic S, Tiozzo S, Lauzon RJ. 2011. Asexual propagation and regeneration in colonial ascidians. Biol Bull. 221(1):43-61.
135. Kassmer SH, Rodriguez D, De Tomaso AW. 2016. Colonial ascidians as model organisms for the study of germ cells, fertility, whole body regeneration, vascular biology and aging. Curr Opin Genet Dev. 39:101-106.
136. Kassmer SH, Nourizadeh S, De Tomaso AW. 2019. Cellular and molecular mechanisms of regeneration in colonial and solitary ascidians. Dev Biol. 448(2):271-278.
137. Voskoboynik A, Simon-Blecher N, Soen Y, Rinkevich B, De Tomaso AW, Ishizuka KJ, Weissman IL. 2007. Striving for normality: Whole body regeneration through a series of abnormal zooidal generations. FASEB Journal. 21:1335-1344.
138. Rinkevich Y, Paz G, Rinkevich B, Reshef R. 2007. Systemic bud induction and retinoic acid signaling underlie whole body regeneration in the urochordate *Botrylloides leachi*. PLoS Biol. 5: 900-913.
139. Rinkevich Y, Rinkevich B, Reshef R. 2008. Cell signaling and transcription factor genes expressed during whole body regeneration in a colonial chordate. BMC Dev Biol. 8:100.
140. Rinkevich Y, Rosner A, Rabinowitz C, Lapidot Z, Moiseeva E, Rinkevitch B. 2010. Piwi positive cells that line the vasculature epithelium, underlie whole body regeneration in a basal chordate. Dev Biol. 345:94-104.
141. Kassmer SH, Langenbacher AD, De Tomaso AW. 2020. Integrin-alpha-6+ Candidate stem cells are responsible for whole body regeneration in the invertebrate chordate *Botrylloides* *diegensis*. Nat Commun. 11(1):4435.
142. Kaneko N, Katsuyama Y, Kawamura K, Fujiwara S. 2010. Regeneration of the gut requires retinoic acid in the budding ascidian *Polyandrocarpa misakiensis*. Dev Growth Diff. 52:457-468.
143. Scelzo M, Alié A, Pagnotta S, Lejeune C, Henry P, Gilletta L, Hiebert LS, Mastrototaro F, Tiozzo S. 2019. Novel budding mode in *Polyandrocarpa zorritensis*: a model for comparative studies on asexual development and whole body regeneration. EvoDevo. 10:7.
144. Jeffery WR. 2015*b*. Regeneration, stem cells, and aging in the tunicate *Ciona*: insights from the oral siphon. Int Rev Cell Mol Biol. 319:255-82.
145. Jeffery WR. 2019. Progenitor targeting by adult stem cells in *Ciona* homeostasis, injury, and regeneration. Dev Biol. 448(2):279-290.
146. Freeman G. 1964. The role of blood cells in the process of asexual reproduction in the tunicate *Perophora viridis.* J Exp Zool. 156:157-83.
147. Iguchi N, Kidokoro H. 2006. Horizontal distribution of *Thetys vagina Tilesius* (Tunicata, Thaliacea) in the Japan Sea during spring 2004. J Plankton Res. 28:537-541.
148. Shenkar N, Koplovitz G, Dray L, Gissi C, Huchon D. 2016. Back to solitude: Solving the phylogenetic position of the Diazonidae using molecular and developmental characters. Mol Phylogenet Evol. 100:51-6.
149. Somorjai IML, Somorjai RL, Garcia-Fernandez J, Escriva H. 2012a. Vertebrate-like regeneration in the invertebrate chordate amphioxus. Proc Natl Acad Sci USA. 109:517-522.
150. Somorjai IM, Escrivà H, Garcia-Fernàndez J. 2012b. Amphioxus makes the cut-Again. Commun Integr Biol. 5: 499-502.
151. Somorjai IML. 2017. Amphioxus regeneration: evolutionary and biomedical implications. Int J Dev Biol. 61(10-11-12):689-696.
152. Zhang Q, Li G, Sun Y, Wang Y. 2009. Chromosome preparation and preliminary observation of two amphioxus species in Xiamen. Zool Res. 30:131-136.
153. Liang Y, Rathnayake D, Huang S, Pathirana A, Xu Q, Zhang S. 2019. BMP signaling is required for amphioxus tail regeneration. Development 146(4): dev166017.
154. Kaneto S, Wada H. 2011. Regeneration of amphioxus oral cirri and its skeletal rods: implications for the origin of the vertebrate skeleton. J Exp Zool B Mol Dev Evol. 316:409-417.
155. Silva JR, Mendes EG, Mariano M. 1995. Wound repair in the Amphioxus (*Branchiostoma platae*), an animal deprived of inflammatory phagocytes. J Invertebr Pathol. 65: 147-151.
156. Silva JR, Mendes EG, Mariano M. 1998. Regeneration in the amphioxus (*Branchiostoma platae*). Zoologischer Anzeiger 237: 107-112.
157. Andrews EA. 1893. An undescribed acraniate, *Asymmetron lucayanum*. Stud Biol Lab Johns Hopkins Univ. 5:213-247.
158. Christen B, Robles V, Raya M, Paramonov I, Izpisua Belmone JC. 2010. Regeneration and reprogramming compared. BMC Biol. 8:5.
159. Marques I, Lupi E, Mercader N. 2019. Model systems for regeneration: zebrafish. Development. 146(18):dev167692.
160. Nobuyasu M, Suetsugu-Maki R, Agata K, Rio-Tsonis K, Tsonis P. 2009. Expression of stem cell pluripotency factors during regeneration in newts. Dev Dyn. 238:1613-1616.
161. Tanaka EM, Reddien PW. 2011. The cellular basis for animal regeneration. Dev Cell. 21:172–185.
162. Zhu W, Pao G, Satoh A, Cummings G, Monaghan J, Harkins T, Bryant S, Voss S, Gardiner D, Hunter T. 2012. Activation of germline-specific genes is required for limb regeneration in the Mexican axolotl. Dev biol. 370: 42-51.
163. Sandoval-Guzman T, Wang H, Khattak S, Schuez M, Roensch K, Nacu E, Tazaki A, Joven A, Tanaka EM, Simon A. 2014. Fundamental differences in dedifferentiation and stem cell recruitment during skeletal muscle regeneration in two salamander species. Cell Stem Cell. 14:174-187.
164. Gerber T, Murawala P, Knapp D, Masselink W, Schuez M, Hermann S, Gac-Santel M, Nowoshilow S, Kageyama J, Khattak S, Currie JD, Camp JG, Tanaka EM, Treutlein B. 2018. Single-cell analysis uncovers convergence of cell identities during axolotl limb regeneration. Science. 362(6413):eaaq0681.
165. Joven A, Elewa A, Simon A. 2019. Model systems for regeneration: salamanders. Development. 146(14):dev167700.
