# Supplementary material for: A pan‐metazoan concept for adult stem cells: the wobbling Penrose landscape
Source: Biol Rev Camb Philos Soc. 2021 Oct 6;97(1):299–325. doi: 10.1111/brv.12801 (PMC9292022; doi:10.1111/brv.12801)
Supplement: Supplementary file 8 — Table S6. Overview of the involvement of adult stem cell (ASCs) and progenitors in agametic asexual reproduction (budding, fission/fragmentation) in metazoans. [file BRV-97-299-s003.docx]

**Table S6.** Overview of the involvement of adult stem cells (ASCs) and progenitors in agametic asexual reproduction (budding, fission/fragmentation) in metazoans. The presence and type of agametic asexual reproduction, the cell types and putative ASCs or progenitors involved, their level of differentiation and potency, as well as expressed ‘stemness’ gene families are reported for Metazoan phyla. Although every effort was made to include all representative data, the literature is not exhaustive particularly for vertebrates and model ecdysozoans. Red font: high levels of confidence concerning ASC involvement. Orange font: middle levels of confidence concerning ASC involvement. Black font: reported, but no level of confidence established. ASCs can be characterized as undifferentiated (U) or differentiated (D) cell types. For budding modes, B = budding and F = any form of fission/fragmentation. ? = uncertain data.

| **Taxon** | **Species** | **Mode: budding (B),**  **fission (F), fragmentation (FR)** | **Type of budding/**  **fission** | **Cellular contribution** | | | | | | **Cellular process: dedifferentiation, transdifferentiation** | | **Reference** | |
| --- | --- | --- | --- | --- | --- | --- | --- | --- | --- | --- | --- | --- | --- |
|  |  |  |  | **Cell type** | **Origin** | **‘Stemness’ gene family expression** | **Putative ASC or progenitor?** | **Potency** |  | |  | |  |
| **PORIFERA** |  |  |  |  |  |  |  |  |  | |  | |  |
| Demospongiae | *Tethya aurantium*, *Tethya citrina*, *Haliclona fulva*, *Suberites domuncula*, | B | budding | archaeocytes, choanocytes | U, D | piwi | yes= archeocytes, choanocytes | multipotent/  multipotent; gemmular archaeocytes are totipotent | dedifferentiation/ transdifferentiation | | 1–4 | |  |
|  | *Chondrosia reniformis* | F | creeping | unknown | unknown | unknown | unknown | unknown | unknown | | 5 | |  |
| Hexactinellida | *Rhabdocalyptus dawsoni* | B | budding | unknown | unknown | unknown | unknown | unknown | unknown | | 6 | |  |
| Calcarea | *Clathrina aurea*, *Sycettusa hastifera* | B, F | budding/  fragmentation | choanocytes? | D | unknown | yes = choanocytes? | unknown | dedifferentiation | | 7, 8 | |  |
| Homoscleromorpha | *Oscarella lobularis* | B | budding | all cell types | D | *piwi*/*argonaute*, *vasa*/*pl10*, *nanos* | yes = choanocytes? | pluripotent | dedifferentiation, transdifferentiation | | 9, 10 | |  |
| **PLACOZOA** |  |  |  |  |  |  |  |  |  | |  | |  |
|  | *Trichoplax adhaerens* | F | binary/  multiple | unknown | unknown | unknown | unknown | unknown | unknown | | 11, 12 | |  |
| **CNIDARIA** |  |  |  |  |  |  |  |  |  | |  | |  |
| Hydrozoa | *Hydra* spp. | B | polyp–polyp | i-cells | U | unknown | yes = i-cells | multipotent | unknown | | 13–16 | |  |
|  | *Hydra* spp. | B | polyp–polyp | epithelial cells | D | unknown | yes = epithelial cells | multipotent | dedifferentiation | | 13–16 | |  |
|  | *Hydractinia echinata* | B | polyp–polyp | i-cells | U | unknown | yes = i-cells | pluripotent/  totipotent | unknown | | 17–19 | |  |
|  | *Podocoryne carnea* | B | polyp–medusa | epithelial cells | D | *piwi* (*Cniwi*) | yes = epithelial cells | multipotent | dedifferentiation | | 20 | |  |
|  | *Nanomia bijuga* (Siphonophora) | B | zooid budding | i-cells | U | *vasa*, *pl10*, *piwi*, *nanos* | yes = i-cells | unknown | unknown | | 21 | |  |
|  | other (i-cell-lacking hydrozoa) | B, F | polyp–polyp,  polyp–medusa | epithelial cells? | D | unknown | unknown | multipotent | dedifferentiation | | 22 | |  |
|  | Bougainvillioidea | F | polyp–polyp | unknown | unknown | unknown | unknown | unknown | unknown | | 23 | |  |
| Schyphozoa | *Aurelia* sp., *Chrysaora chesapeakei*, *Cassiopea xamachana*, several monodisc and polidisc strobilator species | B | polyp–medusa, polyp–planuloid  (strobilation) | amoebocytes? epithelial cells? | U, D | unknown | unknown | unknown | transdifferentiation of epithelial cells? | | 24–27 | |  |
|  | *Aurelia aurita* | B | polyp–medusa (strobilation) | amoebocytes? epithelial cells? | unknown | unknown | unknown | unknown | unknown | | 28 | |  |
| Cubozoa | *Carybdea marsupialis* | B | polyp–medusa | unknown | unknown | unknown | unknown | unknown | unknown | | 29 | |  |
| Staurozoa | *Haliclystus antarcticus* | B |  | unknown | unknown | unknown | unknown | unknown | unknown | | 30, 31 | |  |
| Hexacorallia | *Acropora* sp., *Porites* sp., *Pocillopora damicornis* | B, F | polyp–polyp | amoebocytes? epithelial cells? | D | unknown | unknown | unknown | transdifferentiation of epithelial cells? | | 32, 33, 26 | |  |
|  | *Nematostella vectensis* | F | architomy (physal pinching)/  paratomy (reversed polarity) | unknown | unknown | unknown | unknown | unknown | unknown | | 34, 35 | |  |
| Octocorallia | *Gersemia rubiformis* | B, F | polyp–polyp | unknown | unknown | unknown | unknown | unknown | unknown | | 36, 37 | |  |
| **CTENOPHORA** |  |  |  |  |  |  |  |  |  | |  | |  |
| Platyctenida | *Coeloplana waltoni*, *Vallicula multiformis* | F | fragmentation | unknown | unknown | unknown | unknown | unknown | unknown | | 38 | |  |
| **ACOELOMORPHA** |  |  |  |  |  |  |  |  |  | |  | |  |
|  | *Convolutriloba retrogemma*, | B | (reversed polarity) budding | neoblasts | U | unknown | yes = neoblast | pluripotent/  totipotent | unknown | | 39–41 | |  |
|  | *Convolutriloba longifissura*, *Amphiscolops langerhansi*, *Adenopea cenata* | F | architomy | neoblast?  differentiated cells? | U, D ?? | unknown | yes = neoblast? | unknown | dedifferentiation? | | 40 | |  |
|  | *Paratomella unichaeta* | F | paratomy | unknown | unknown | unknown | unknown | unknown | unknown | | 42 | |  |
| **PLATYHELMINTHES** |  |  |  |  |  |  |  |  |  | |  | |  |
| Catenulida | *Catenula* | F | paratomy | neoblasts? epithelial stem cells? | U/D | unknown | yes= neoblast? | unknown | unknown | | 43 | |  |
| Macrostomorpha | *Microstomum lineare* | F | paratomy | neoblasts | U | unknown | yes= neoblast? | supposed to be pluripotent | unknown | | 44, 45 | |  |
| Tricladida | *Dugesia tahitiensis*, | F | architomy/  paratomy | neoblasts? | U | unknown | yes= neoblast? | supposed to be pluripotent | unknown | | 45, 46 | |  |
|  | *Dugesia japonica* | F | architomy | neoblasts? | U | unknown | yes= neoblast? | supposed to be pluripotent | unknown | | 47, 48, 49 | |  |
|  | *Dugesia dorotocefala* | F | architomy | neoblasts? | U | unknown | yes= neoblast? | supposed to be pluripotent | unknown | | 50 | |  |
|  | *Dugesia tigrina* | F | architomy | neoblasts | U | unknown | yes= neoblast? | supposed to be pluripotent | unknown | | 51 | |  |
| Cestoda | *Mesocestoides* | F/B | architomy (longitudinal fission)/ budding | unknown | unknown | unknown | unknown | unknown | unknown | | 44 | |  |
| **NEMERTEA** |  |  |  |  |  |  |  |  |  | |  | |  |
| Pilidiophora | *Lineus* sp*.*, *Baseodiscus hemprichii* | F | architomy | unknown | unknown | unknown |  | unknown | unknown | | 52–54 | |  |
| **ANNELIDA** |  |  |  |  |  |  |  |  |  | |  | |  |
| Sedentaria | *Pristina longiseta*, *Nais communis* | F | paratomy | dedifferentiated epidermal cells | D? | *vasa*, *pl10*, and *piwi* | unknown | unknown | dedifferentiation? | | 55–58 | |  |
|  | *Pristina leidyi* | F | paratomy | unknown | unknown | *piwi*, *vasa*, *nanos* | unknown | unknown | unknown | | 59, 60 | |  |
|  | *Paranais litoralis* | F | paratomy | unknown | unknown | *nanos* | unknown | unknown | unknown | | 61 | |  |
|  | *Enchytraeus japonensis* | F | architomy | neoblast, N-cells, epidermal and intestinal cells? | U, D | *piwi* | yes = neoblast-like | multipotent? | dedifferentiation? | | 62–64 | |  |
| Errantia | *Pygospio elegans* | F | architomy | unclear | D | unknown | unknown | unknown | dedifferentiation?? | | 65 | |  |
|  | *Procerastea halleziana*, *Syllis gracilis* | F | fragmentation | unknown | unknown | unknown | unknown | unknown | unknown | | 59 | |  |
| Sipuncula | *Sipunculus robustus* | B | posterior budding | unknown | unknown | unknown | unknown | unknown | unknown | | 66 | |  |
| **BRACHIOPODA** |  |  |  |  |  |  |  |  |  | |  | |  |
|  |  | no fission/  fragmentation/budding |  |  |  |  |  |  |  | |  | |  |
| **PHORONIDA** |  |  |  |  |  |  |  |  |  | |  | |  |
|  | *Phoronis ovalis* | F, B | architomy/budding | unknown | unknown | unknown | unknown | unknown | dedifferentiation? | | 67 | |  |
| **ECTOPROCTA** |  |  |  |  |  |  |  |  |  | |  | |  |
|  | *Hislopia malyensis*, *Cristatella mucedo*, *Lophopus crystallinus* | B | developing cistid budding (similar to tunicate vascular budding) | epidermal cells?, peritoneal (coelomic) cells?, undifferentiated coelomocytes? | U,D | unknown | unknown | multipotent | dedifferentiation | | 68, 69 | |  |
|  | *Cupuladria exfragminis* | F | auto-fragmentation | unknown | unknown | unknown | unknown | unknown | unknown | | 70 | |  |
| **GASTROTRICHA** |  |  |  |  |  |  |  |  |  | |  | |  |
|  |  | no fission/ fragmentation/budding |  |  |  |  |  |  |  | |  | |  |
| **MOLLUSCA** |  |  |  |  |  |  |  |  |  | |  | |  |
|  |  | no fission/  fragmentation/budding |  |  |  |  |  |  |  | |  | |  |
| **ENTOPROCTA** |  |  |  |  |  |  |  |  |  | |  | |  |
|  | *Loxosomella* sp. | B | budding | unknown | unknown | unknown | unknown | unknown | unknown | | 71 | |  |
|  | *Pedicellina* sp. | B | stolonial | epidermal cell? | D | unknown | unknown | unknown | dedifferentiation? | |  | |  |
| **CHAETOGNATHA** |  |  |  |  |  |  |  |  |  | |  | |  |
|  |  | no fission/  fragmentation/budding |  |  |  |  |  |  |  | |  | |  |
| **ROTIFERA** |  |  |  |  |  |  |  |  |  | |  | |  |
|  |  |  |  |  |  |  |  |  |  | |  | |  |
| **ARTHROPODA** |  |  |  |  |  |  |  |  |  | |  | |  |
| Crustacea | *Polyascus poligenea* | B | budding | undifferentiated cells | U | *vasa*-related genes (*PpVLG*, *PpDRH1*) | yes = undifferentiated cells | multipotent | unknown | | 72 | |  |
|  | *Peltogastrella gracilis*,*Thylacoplethus isaevae* | B | budding | unknown | unknown | unknown | unknown | pluripotent? | unknown | | 73, 74 | |  |
| **ONYCHOPHORA** |  |  |  |  |  |  |  |  |  | |  | |  |
|  |  | no fission/  fragmentation/budding |  |  |  |  |  |  |  | |  | |  |
| **TARDIGRADA** |  |  |  |  |  |  |  |  |  | |  | |  |
|  |  | no fission/  fragmentation/budding |  |  |  |  |  |  |  | |  | |  |
| **NEMATODA** |  |  |  |  |  |  |  |  |  | |  | |  |
|  |  | no fission/  fragmentation/budding |  |  |  |  |  |  |  | |  | |  |
| **PRIAPULIDA** |  |  |  |  |  |  |  |  |  | |  | |  |
|  |  | no fission/  fragmentation/budding |  |  |  |  |  |  |  | |  | |  |
| **VERTEBRATA** |  |  |  |  |  |  |  |  |  | |  | |  |
|  |  | no fission/  fragmentation/budding |  |  |  |  |  |  |  | |  | |  |
| **UROCHORDATA** |  |  |  |  |  |  |  |  |  | |  | |  |
| Stolidobranchia | *Botryllus* spp. | B | vascular, palleal | circulating ASCs, multipotent epithelia | U | *POU*, *piwi*, *vasa*, *myc* | yes = haemoblasts, epithelial cells | multipotent/  unipotent/  tissue-restricted progenitor | unknown | | 75–82 | |  |
|  | *Botrylloides* spp. | B | vascular, palleal | circulating ASCs, multipotent epithelia | U | piwi, vasa | yes = haemoblasts, epithelial cells | multipotent/  unipotent | unknown | | 83–85 | |  |
|  | *Polyandrocarpa* spp. | B | vascular, palleal | circulating ASCs, multipotent epithelia | U, D | myc | yes = haemoblasts, epithelial cells | multipotent | transdifferentiation/ dedifferentiation | | 86–91 | |  |
|  | *Polyandrocarpa zorritensis* | B | vasal | circulating ASCs, multipotent epithelia | U/D | unknown | yes = haemoblasts, epithelial cells | unknown | unknown | | 118 | |  |
|  | *Symplegma brakenhielmi* | B | vascular, palleal | circulating ASCs, multipotent epithelia | U | unknown | ye s= haemoblasts, epithelial cells | multipotent | unknown | | 92 | |  |
| Phlebobranchia | *Perophora* | B | stolonial | circulating ASCs | U | unknown | yes = haemoblasts | multipotent | unknown | | 90, 93 | |  |
| Aplousobranchia | *Aplidium* spp., *Pycnoclavella* spp*.*, *Diplosoma listerianum* | B | stolonial, pyloric budding, strobilation | no clear evidence |  | unknown | unknown | unknown | unknown | | 94 | |  |
|  | *Clavelina gemmae* | B | vascular | circulating ASCs | U | unknown | yes = haemoblasts | multipotent | unknown | | 95 | |  |
| Thaliacea | *Pyrosoma* spp., *Doliolum* spp., Salpa spp. | B |  | no clear evidence |  | unknown | unknown | unknown | unknown | | 94 | |  |
| **CEPHALOCHORDATA** |  |  |  |  |  |  |  |  |  | |  | |  |
|  |  | no fission/  fragmentation/ budding |  |  |  |  |  |  |  | |  | |  |
| **XENOTURBELLIDA** |  |  |  |  |  |  |  |  |  | |  | |  |
|  | *Xenoturbella* | F | longitudinal fission | undifferentiated cells? | U | unknown | unknown | unknown | unknown | | 96 | |  |
| **ECHINODERMATA** |  |  |  |  |  |  |  |  |  | |  | |  |
| Asteroidea | *Allostichaster capensis* | F | architomy | dedifferentiating stump cell? | D | unknown | unknown | unknown | dedifferentiation? | | 97 | |  |
|  | *Coscinasterias* sp. | F | architomy | dedifferentiating stump cell? | D | unknown | unknown | unknown | dedifferentiation? | | 98–100 | |  |
|  | *Sclerasterias*, *Stephanasterias*, *Asterina wega* | F | architomy | unknown | unknown | unknown | unknown | unknown | unknown | | 101 | |  |
|  | *Linkia sp.*, *Coscinasterias* sp. | F | clonal autotomy | unknown | unknown | unknown | unknown | unknown | unknown | | 102–104 | |  |
| Ophiuroidea | *Ophiocomella ophiactoides* | F | architomy | unknown | unknown | unknown | unknown | unknown | unknown | | 105 | |  |
|  | *Ophioactis savignyi* | F | architomy | unknown | unknown | unknown | unknown | unknown | unknown | | 106 | |  |
|  | *Ophiotela mirabilis* | F | architomy | unknown | unknown | unknown | unknown | unknown | unknown | | 107 | |  |
| Holothuroidea | Aspidochirotida and Dendrochirotida, several species (16): e.g. *Holothuria* sp., *Stichopus* sp., *Ocnus* sp. | F | architomy (transverse) | dedifferentiating enterocytes/  epidermal cells | D | unknown | unknown | progenitors? | dedifferentiation | | 108 | |  |
|  | *Holothuria atra*, *Bohadschia marmorata* | F | architomy (transverse) | unknown | unknown | unknown | unknown | unknown | dedifferentiation | | 109, 110 | |  |
|  | *Cladolabes schmeltzii* | F | architomy (transverse) | dedifferentiating stump cells? | D | unknown | unknown | unknown | dedifferentiation, epithelial morphogenesis | | 111, 112 | |  |
| **HEMICHORDATA** |  |  |  |  |  |  |  |  |  | |  | |  |
| Enteropneusta | *Balanoglossus* spp., *Glossobalanus crozieri*, *Ptychodera* | F | architomy | unknown | unknown | unknown | unknown | unknown | unknown | | 113–115 | |  |
| Pterobranchia | colonial pterobranch (*Cephalodiscus*, *Rhabdopleura*) | B | stolonial or stalk budding | unknown | unknown | unknown | unknown | unknown | unknown | | 116, 117 | |  |

**References**

1. Ereskovsky AV, Geronimo A, Pérez T. 2017*a*. Asexual and puzzling sexual reproduction of the Mediterranean sponge *Haliclona fulva* (Demospongiae): life cycle and cytological structures. Invert Biol. 136(4):403-421.
2. Gaino E, Scalera-Liaci L, Sciscioli M, Corriero G. 2006. Investigation of the budding process in *Tethya citrina* and *Tethya aurantium* (Porifera, Demospongiae). Zoomorphology. 125:87-97.
3. Müller WEG. 2006. The stem cell concept in sponges (Porifera): Metazoan traits. Semin Cell Dev Biol .17(4):481-91.
4. Ereskovsky AV. 2003. Problems of coloniality, modularity, and individuality in sponges and special features of their morphogeneses during growth and asexual reproduction. Russ J Mar Biol. 29(1):46-56.
5. Fassini D, Parma L, Wilkie IC, Bavestrello G, Bonasoro F, Candia Carnevali MD. 2012. Ecophysiology of mesohyl creep in the demosponge *Chondrosia reniformis* (Porifera:Chondrosida). J Exp Mar Biol Ecol. 428:24-31.
6. Ereskovsky. AV. 2010. *The comparative embryology of sponges*. Dordrecht: Springer.
7. Padua A, Leocorny P, Custódio MR, Klautau MJ. 2016. Fragmentation, fusion, and genetic homogeneity in a calcareous sponge (Porifera, Calcarea). Exp Zool A Ecol Genet Physiol. 325(5):294-303.
8. Lanna E, Klautau M. 2019. The choanoderm of *Sycettusa hastifera* (Calcarea, Porifera) is able to generate new individuals. Invert Biol. 138:e12262.
9. Ereskovsky AV and Tokina DB 2007. Asexual reproduction of homoscleromorph sponges (Porifera; Homoscleromorpha). Mar. Biol. 151:425-434.
10. Fierro-Constaín L, Schenkelaars Q, Gazave E, Haguenauer A, Rocher C, Ereskovsky A, Borchiellini C, Renard E. 2017. The conservation of the germline multipotency program, from sponges to vertebrates:a stepping stone to understanding the somatic and germline origins. Genome Biol Evol. 9(3):474-488.
11. Pearse VB. 1999. Placozoa. In: Knobil E, Neill JD, editors. Encyclopedia of reproduction, vol. 3. San Diego: Academic Press. p. 898-901.
12. Zuccolotto-Arellano J, Cuervo-González R. 2020. Binary fission in *Trichoplax* is orthogonal to the subsequent division plane. Mech Dev. 162:103608.
13. Bosch TCG, Anton-Erxleben F, Hemmrich G, Khalturin K. 2010. The *Hydra* polyp: nothing but an active stem cell community. Dev Growth Differ. 52:15-25.
14. Bode HR, David CN.1978. Regulation of a multipotent stem cell, the interstitial cell of *Hydra.* Prog Biophys Mol Biol. 33:189-206.
15. Holstein TW, Hobmayer E, David CN. 1991. Pattern of epithelial cell cycling in *Hydra.* Dev Biol. 148:602-11.
16. Khalturin K, Anton-Erxleben F, Milde S, Plötz C, Wittlieb J, Hemmrich G, Bosch TC. 2007. Transgenic stem cells in *Hydra* reveal an early evolutionary origin for key elements controlling self-renewal and differentiation. Dev Biol. 309:32-44.
17. Plickert G, Frank U, Müller WA 2012. *Hydractinia*, a pioneering model for stem cell biology and reprogramming somatic cells to pluripotency. Int J Dev Biol. 56:519-534.
18. Kuenzel T, Heiermann R, Frank U, Müller WA, Tilmann W, Bause M, Nonn A, Helling M, Schwarz RS and Plickert G. 2010. Migration and differentiation potential of stem cells in the cnidarian *Hydractinia* analysed in eGFP-transgenic animals and chimeras. Dev Biol. 348:120-129.
19. Müller WA, Frank U, Teo R, Mokady O, Guette C. and Plickert,G. 2007. Wnt signaling in hydroid development: ectopic heads and giant buds induced by GSK-3 beta inhibitors. Int J Dev Biol. 1:211-220.
20. Seipel K, Yanze N, Schmid V. 2004. The germ line and somatic stem cell gene Cniwi in the jellyfish Introduction *Podocoryne carnea*. Int J Dev Biol. 48:1-7.
21. Siebert S, Goetz FE, Church SH, Bhattacharyya P, Zapata F, Haddock SHD, Dunn CW. 2015. Stem cells in *Nanomia bijuga* (Siphonophora), a colonial animal with localized growth zones. EvoDevo. 6:22.
22. Boero F, Bouillon J, Piraino S., Schmid V. 2002. Asexual reproduction in the hydrozoa (Cnidaria). In:KG and RG Adiyodi and RN Huges, editors. Reproductive biology of invertebrates - Progress in Asexual Reproduction. New Delhi: Oxford & IBH Publishing. p. 141-158.
23. Bavestrello G, Puce S, Cerrano C, Senes L. 2000. Strobilation in a species of *Bougainvillioidea* (Cnidaria:Hydrozoa). Sci Mar. 164(1):147-150.
24. Fautin DG. 2002. Reproduction of Cnidaria. Can J Zool. 80(10):1735-1754.
25. Frank U, Plickert G, Müller WA. 2009. Cnidarian interstitial cells: the dawn of stem cell research. In: Rinkevich B and Matranga V, editors. Stem Cells in Marine Organisms. Dordrecht: Springer.
26. Gold DA, Jacobs DK. 2013. Stem cell dynamics in Cnidaria:are there unifying principles? Dev Genes Evol. 223:53-66.
27. Helm RR. 2018. Evolution and development of scyphozoan jellyﬁsh. Biol Rev. 93:1228-1250.
28. Fuchs B, Wang W, Graspeuntner S, Li Y, Insua S, Herbst EM, Dirksen P, Böhm AM, Hemmrich G, Sommer F et al. 2014. Regulation of Polyp-to-Jellyﬁsh Transition in *Aurelia aurita*. Curr Biol. 24:263-273.
29. Fischer AB, Hofmann DK. 2004. Budding, bud morphogenesis, and regeneration in *Carybdea marsupialis* Linnaeus, 1758 (Cnidaria:Cubozoa). Hydrobiologia, 530: 331-337.
30. Miranda LS, Collins AG and Marques AC. 2010. Molecules clarify a cnidarian life cycle - the “hydrozoan” *Microhydrula limopsicola* is an early life stage of the staurozoan *Haliclystus antarcticus*. PLoS ONE, 5(4):e10182.
31. Miranda LS, Morandini AC, Marques AC. 2012. Do Staurozoa bloom? A review of stauromedusan population biology. Hydrobiol. 690 (1):57-67.
32. Highsmith RC. 1982. Reproduction by fragmentation in corals. Mar Ecol Prog Ser 7:207-226.
33. Shafir SJ, Van Rijn J, Rinkevich B. 2001. Nubbing of coral colonies:a novel approach for the development of island broodstocks. Aquar Sci and Conserv 3:183-190.
34. Burton PM, Finnerty JR. 2009. Conserved and novel gene expression between regeneration and asexual fission in *Nematostella vectensis*. Dev Genes Evol. 219(2):79‐87.
35. Reitzel AM, Burton PM, Krone C, Finnerty JR. 2007. Comparison of developmental trajectories in the starlet sea anemone *Nematostella vectensis*: embryogenesis, regeneration, and two forms of asexual fission. Invert Biol. 126:99-112.
36. Lasker H. 1984. Asexual reproduction, fragmentation, and skeletal morphology of a plexaurid gorgonian. Mar Ecol Progr Ser. 19(3):261-268.
37. Henry L-A, Kenchington EL, Silvaggio A. 2003. Effects of mechanical experimental disturbance on aspects of colony responses, reproduction, and regeneration in the cold-water octocoral *Gersemia rubiformis*. Can J Zool. 81:1691-1701.
38. Glynn PW, Coffman B, Primov K, Renegar DA, Gross J, Blackwelder P, Martinez N, Dominguez J, Vanderwoude J, Riegl BM. 2019. Benthic ctenophore (Order *Platyctenida*) reproduction, recruitment, and seasonality in south Florida. Invert Biol. 138(3):e12256.
39. Sikes JM, Bely AE. 2010. Making heads from tails: development of a reversed anterior-posterior axis during budding in an acoel. Dev Biol. 338(1):86-97.
40. Åkesson B, Gschwentner R, Hendelberg J, Ladurner P, Müller J, Rieger R. 2001. Fission in *Convolutriloba longiﬁssura*: asexual reproduction in acoelous turbellarians revisited. Acta Zool. 82:31-39.
41. Hendelberg J and Åkesson B. 1988. *Convolutriloba retrogemma* gen. et sp.n., a turbellarian (Acoela, Platyhelminthes) with reversed polarity of reproductive buds. Fortschritte der Zoologie 36:321-327.
42. Egger B, Gschwentner R, Rieger R. 2007. Free-living flatworms under the knife:past and present. Dev Genes Evol. 217:89-104.
43. Moraczewski, 1977. Asexual reproduction and regeneration of *Catenula* (Turbellaria, Archoophora). J Zoomorph. 88:65-80.
44. Reuter M, Kreshchenko N. 2004. Flatworm asexual multiplication implicates stem cells and regeneration. Can J Zool. 82:334-356.
45. Peter R, Ladurner P, Rieger RM. 2001. The role of stem cell strategies in coping with environmental stress and choosing between alternative reproductive modes: Turbellaria rely on a single cell type to maintain individual life and propagate species. Mar Ecol. 22(1-2):35-51.
46. Sakurai T, Lee H, Kashima M, Saito Y, Hayashi T, Kudome-Takamatsu T, Nishimura O, Agata K, Shibata N. 2012. The planarian P2X homolog in the regulation of asexual reproduction. Int J Dev Biol. 56(1-3):173-82.
47. Hori I, Kishida Y. 1998. A fine structural study of regeneration after fission in the planarian *Dugesia japonica*. Hydrobiologia. 383:131-136.
48. Hori I, Kishida Y. 2001. Further observation on the early regenerates after fission in the planarian *Dugesia japonica*. Belg J Zool. 131(1):117-121.
49. Malinowski PT, Cochet-Escartin O, Kaj KJ, Ronan E, Groisman A, Diamond PH, Collins ES. 2017. Mechanics dictate where and how freshwater planarians fission. Proc Natl Acad Sci USA. 114(41):10888-10893.
50. Nentwig MR. 1978. Comparative morphological studies of head development after decapitation and after fission in the planarian *Dugesia dorotocephala*. Trans Am Microsc Soc. 97:297-310.
51. Krichinskaya EB, Martynova MG. 1975. Distribution of neoblasts and mitoses during the asexual reproduction of the planarian *Dugesia tigrina* (Girard). Sov J Dev Biol. 5(4):309‐314.
52. Kajihara H, Hookabe N. 2019. Anterior Regeneration in *Baseodiscus hemprichii* (Nemertea: Heteronemertea). Trop Nat Hist. 19(1):39-42,
53. Coe WR. 1930. Asexual reproduction in nemerteans. Physiol Zool. 3:297-308.
54. Gontcharoff M. 1951. Biologie de la régénération et de la reproduction chez quelques Lineidae de France. Ann Sci Nat Zool. Série 11. 13:149-235.
55. Bely AE, Wray GA. 2001. Evolution of regeneration and fission in annelids: insights from engrailed-and orthodenticle-class gene expression. Development. 128:2781-279.
56. Smirnova NP, Kostyuchenko RP. 2007. Cellular sources of paratomy zone in oligochaetes *Pristina longiseta* (Naididae): cloning and analysis of expression of genes markers of stem and undifferentiated cells. Tsitologiya. 49(9):794.
57. Kostyuchenko RP, Kozin VV, Kupriashova EE. 2016. Regeneration and asexual reproduction in annelids:cells, genes, and evolution. Biol Bull. 43:185-194.
58. Kharin A, Zagainova I, Kostyuchenko R. (2006). Formation of the paratomic fission zone in freshwater oligochaetes. Russ J Dev Biol. 37:354-365.
59. Ribeiro RP, Bleidorn C, Aguado MT. 2018*a*. Regeneration mechanisms in Syllidae (Annelida). Regeneration. 5(1):26‐42.
60. Özpolat BD, Bely AE. 2015. Gonad establishment during asexual reproduction in the annelid *Pristina leidyi*. Dev Biol. 405(1):123-36.
61. Bely AE and Sikes JM. 2010*b*. Latent regeneration abilities persist following recent evolutionary loss in asexual annelids. Proc Natl Acad Sci USA, 107(4):1464-1469.
62. Sugio M, Yoshida-Noro C, Ozawa K, Tochinai S. 2012. Stem cells in asexual reproduction of *Enchytraeus japonensis* (Oligochaeta, Annelid): proliferation and migration of neoblasts. Dev Growth Differ. 54(4):439-50.
63. Yoshida-Noro C, Tochinai S. 2010. Stem cell system in asexual and sexual reproduction of *Enchytraeus japonensis* (Oligochaeta, Annelida). Dev Growth Differ. 52(1):43-55.
64. Tadokoro R, Sugio M, Kutsuna J, Tochinai S, Takahashi Y.2006. Early segregation of germ and somatic lineages during gonadal regeneration in the annelid *Enchytraeus japonensis*. Curr Biol. 16(10):1012-1017.
65. Gibson GD, Harvey JML. 2000. Morphogenesis during asexual reproduction in *Pygospio elegans* Claparede (Annelida, Polychaeta). Biol Bull. 199(1):41-49.
66. Rajulu G, Krishnan N. 1969. Occurrence of asexual reproduction by budding in sipunculida. Nature. 223:186-187.
67. Zimmer R. 1999. Phoronida. In:Knobil E, Neill JD, editors. Encyclopedia of reproduction. San Diego: Academic Press.
68. Schwaha T, Wood TS. 2011. Organogenesis during budding and lophophoral morphology of *Hislopia malayensis* Annandale,1916 (Bryozoa, Ctenostomata). BMC Dev Biol. 11:23.
69. Schwaha T, Handschuh S, Redl E, Walzl MG. 2011. Organogenesis in the budding process of the freshwater bryozoan *Cristatella mucedo* Cuvier, 1798 (Bryozoa, Phylactolaemata). J Morphol. 272(3):320-41.
70. O’Dea A. 2006. Asexual propagation in the marine bryozoan *Cupuladria exfragminis*. J Exp Mar Biol Ecol. 335(2):312-322.
71. Merkel J, Wanninger A, Lieb B. 2018. Novel and conserved features of the Hox cluster of Entoprocta (Kamptozoa). J Phylogenetics Evol Biol. 6:1.
72. Shukalyuk AI, Golovnina KA, Baiborodin SI, Gunbin KV, Blinov AG, Isaeva VV. 2007. Vasa-related genes and their expression in stem cells of colonial parasitic rhizocephalan barnacle *Polyascus lygenea* (Arthropoda: Crustacea: Cirripedia: Rhizocephala). Cell Biol Int. 31(2):97-108.
73. Isaeva VV, Dolganov SM, Shukalyuk AI. 2005*b*. Rhizocephalan barnacles—Parasites of commercially important crabs and other decapods. Russ J Mar Biol. 31(4):215-220.
74. Isaeva VV, Akhmadieva AV, Aleksandrova YN, Shukalyuk AI. 2009. Morphofunctional organization of reserve stem cells providing for asexual and sexual reproduction of invertebrates. Russ J Dev Biol. 40:57-68.
75. Manni L, Zaniolo G, Cima F, Burighel P, Ballarin L. 2007. *Botryllus schlosseri*: a model ascidian for the study of asexual reproduction. Dev Dyn. 236:335-352.
76. Manni L, Anselmi C, Cima F, Gasparini F, Voskoboynik A, Martini M, Peronato A, Burighel P, Zaniolo G, Ballarin L. 2019. Sixty years of experimental studies on the blastogenesis of the colonial tunicate *Botryllus schlosseri*. Devl Biol, 448(2):293-308.
77. Ricci L, Chaurasia A, Lapébie P, Dru P, Helm RR, Copley RR, Tiozzo S. 2016. Identification of differentially expressed genes from multipotent epithelia at the onset of an asexual development. Sci Rep. 6:27357.
78. Kawamura K, Sunanaga T. 2011. Role of Vasa, Piwi, and Myc-expressing coelomic cells in gonad regeneration of the colonial tunicate, *Botryllus primigenus*. Mech Dev.128(7-10):457-470.
79. Kawamura K, Sunanaga T. 2010. Hemoblasts in colonial tunicates: are they stem cells or tissue-restricted progenitor cells? Dev Growth Differ. 52:69-76.
80. Sunanaga T, Inubushi H, Kawamura K. 2010. Piwi-expressing hemoblasts serve as germline stem cells during postembryonic germ cell specification in colonial ascidian, *Botryllus primigenus*. Dev Growth Differ. 52(7):603-14.
81. Oka H, Watanabe H. 1957. Vascular budding, a new type of budding in *Botryllus*. Biol Bull. 112:225-240.
82. Berrill NJ. 1941. The development of the bud in *Botryllus*. Biol Bull. 80:169-184.
83. Cima F, Perin A, Burighel P, Ballarin L. 2001. Morpho-functional characterization of haemocytes of the compound ascidian *Botrylloides leachi* (Tunicata, Ascidiacea). Acta Zool. 82:261-274.
84. Brown FD, Keeling EL, Le AD, Swalla BJ. 2009*a*. Whole body regeneration in a colonial ascidian, *Botrylloides violaceus*. J Exp Zool B Mol Dev Evol. 312(8):885-900.
85. Brown FD, Swalla. 2007. Vasa expression in a colonial ascidian, *Botrylloides violaceus*. Evol Dev. 9(2):165-177.
86. Alié A, Hiebert LS, Simion P, Scelzo M, Prünster MM, Lotito S, Delsuc F, Douzery EJP, Dantec C, Lemaire P et al 2018. Convergent acquisition of nonembryonic development in styelid ascidians. Mol Biol Evol. 35(7):1728-1743.
87. Kawamura K, Fujiwara S. 1995*a*. Cellular and molecular characterization of transdifferentiation in the process of morphallaxis of budding tunicates. Semin Cell Biol. 6:117-126.
88. Kawamura K, Fujiwara S. 1995*b*. Establishment of cell lines from multipotent epithelial sheet in the budding tunicate, *Polyandrocarpa misakiensis*. Cell Struct Funct. 20:97-106.
89. Fujiwara S, Isozaki T, Mori K, Kawamura K. 2011. Expression and function of myc during asexual reproduction of the budding ascidian *Polyandrocarpa misakiensis*. Dev Growth Differ. 53:1004-1014.
90. Kawamura K, Sugino Y, Sunanaga T, Fujiwara S. 2008*a*. Multipotent epithelial cells in the process of regeneration and asexual reproduction in colonial tunicates. Dev Growth Differ. 50:1-11.
91. Kawamura K, Nakauchi M. 1986. Mitosis and body patterning during morphallactic development of palleal buds in ascidians. Dev Biol. 116:39-50.
92. Gutierrez S, Brown FD. 2017. Vascular budding in *Symplegma brakenhielmi* and the evolution of coloniality in styelid ascidians. Dev Biol. 423:152-169.
93. [Freeman G. 1964. The role of blood cells in the process of asexual reproduction in the tunicate *Perophora viridis*. J Exp Zool. 156:157-183.](https://doi.org/10.1002/jez.1401560204)
94. Brown FD, Swalla BJ. 2012. Evolution and development of budding by stem cells: ascidian coloniality as a case study. Dev Biol. 369(2):151-62.
95. Turon X. 2005. A new mode of colony multiplication by modiﬁed budding in the ascidian *Clavelina gemmae* n. sp. (Clavelinidae). Invert Biol. 124:273-283.
96. Israelsson O. 2006. Observations on some unusual cell types in the enigmatic worm *Xenoturbella* (phylum uncertain). Tissue Cell. 38(4):233-242
97. Rubilar T, Pastor de Ward CT, Diaz de Vivar M. 2005. Sexual and asexual reproduction of *Allostichaster capensis* (Echinodermata: Asteroidea) in Golfo Nuevo. Mar Biol. 146:1083-1090.
98. Ducati CC, Carnevali MC, Barker MF. 2004. Regenerative potential and fissiparity in the forcipulate starfish *Coscinasterias muricata*. In: Heinzeller T, Nebelsick JH, editors. Echinoderms. München Taylor & Francis Group, London. p. 113-118.
99. Alves LS, Pereira A, Ventura C. 2002. Sexual and asexual reproduction of *Coscinasterias tenuispina* (Echinodermata:Asteroidea) from Rio de Janeiro, Brazil. Mar Biol. 140:95-101.
100. Emson RH, Wilkie IC. 1980. Fission and autotomy in echinoderms. Ocean Mar Biol Ann Rev. 18:155-250.
101. Hyman LH. 1955. The Invertebrates. IV. Echinodermata, Asteroidea. McGraw Hill, New York.
102. Cortés RY, Hernández R.I, San Martin del Angel P, Zarza Meza E, Cuervo GR. 2016. Regenerative potential of the sea star *Linckia guildinguii*. Hidrobiológica. 26(1):103-108.
103. Shibata D, Hirano Y, Komatsu M. 2011. Life cycle of the multiarmed sea star *Coscinasterias acutispina* (Stimpson, 1862) in laboratory culture: sexual and asexual reproductive pathways. Zool Sci. 28:313-317.
104. Edmonson CH. 1935. Autotomy and regeneration in Hawaiian starfishes. Bernice P. Bishop Museum Occasional Papers. 11(8):3-20.
105. Mladenov PV, Emson RH, Colpit LV, Wilkie IC. 1983. Asexual reproduction in the West Indian brittle star *Ophiocomella ophiactoides* (H. L. Clark) (Echinodermata: Ophiuroidea). J Exp Mar Biol Ecol. 72:1-23.
106. McGovern TM. 2002. Patterns of sexual and asexual reproduction in the brittle star *Ophiactis savignyiin* the Florida Keys. Mar Ecol. 230:119-126.
107. Tavares MR, Costa PAS, Ventura CRR. 2019. Population size structure, asexual reproduction, and somatic growth estimates of the non-indigenous brittle star *Ophiothela mirabilis* (Echinodermata: Ophiuroidea) on the southeastern coast of Brazil. Mar Biodiv. 49:1713-1725.
108. Dolmatov IY. 2014. Asexual reproduction in holothurians. Scientific World J 2014, Article ID 527234.
109. Laxminarayana A. 2006. Asexual reproduction by induced transverse fission in the sea cucumbers *Bohadschia marmorata* and *Holothuria atra*,” SPC Beche-de-Mer Inf Bull. 23:35-37.
110. Conand C. 1995. Asexual reproduction by fission in *Holothuria atra*: variability of some parameters in populations from the tropical lndo-Pacific. Oceanol Acta. 19:3-4.
111. Kamenev YO, Dolmatov IY. 2017. Anterior regeneration after fission in the holothurian *Cladolabes schmeltzii* (Dendrochirotida:Holothuroidea). Microsc Res Tech. 80(2):183-194.
112. Kamenev YO, Dolmatov IY. 2015. Posterior regeneration following fission in the holothurian *Cladolabes schmeltzii* (Dendrochirotida: Holothuroidea). Microsc Res Tech. 78(7):540-52.
113. Miyamoto N, Saito Y. 2010. Morphological characterization of the asexual reproduction in the acorn worm *Balanoglossus simodensis*. Dev Growth Differ. 52:615-627.
114. Petersen JA, Ditadi ASF. 1971. Asexual reproduction in *Glossobalanus crozieri* (Ptychoderidae, Enteropneusta, Hemichordata). Mar Biol 9(1):78-85.
115. Packard A. 1968. Asexual Reproduction in *Balanoglossus* (Stomochordata). Proc. R Soc Lond B*.* 171:261-272.
116. Rychel AL, Swalla BJ. 2009. Regeneration in Hemichordates and Echinoderms. In: Rinkevich B. and Matranga V, editors. Stem cells in marine organisms. Dordrecht: Springer Science+Business Media BV.
117. King GM. 1998. Reproduction in the Hemichordata. In: Knobil E, Neill JD, editors. Encyclopedia of reproduction vol. 2, San Diego: Academic Press. p. 599-603.
118. Scelzo M, Alié A, Pagnotta S, Lejeune C, Henry P, Gilletta L, Hiebert LS, Mastrototaro F, Tiozzo S. 2019. Novel budding mode in *Polyandrocarpa zorritensis*:a model for comparative studies on asexual development and whole body regeneration. Evodevo. 10:7.
