# Supplementary material for: A pan‐metazoan concept for adult stem cells: the wobbling Penrose landscape
Source: Biol Rev Camb Philos Soc. 2021 Oct 6;97(1):299–325. doi: 10.1111/brv.12801 (PMC9292022; doi:10.1111/brv.12801)
Supplement: Supplementary file 9 — Table S7. Overview of the involvement of adult stem cell (ASCs) and progenitors in dormancy in metazoans. [file BRV-97-299-s006.docx]

**Table S7.** Overview of the involvement of adult stem cells ASCs and progenitors in dormancy in metazoans. The presence and type of dormant state, the life cycle stages at which it was observed, the cell types and putative ASCs or progenitors involved, their level of differentiation and potency, as well as expressed stemness gene families are reported for metazoan phyla. Diapause is a physiologically and environmentally controlled type of dormancy resulting in metabolic depression that does not immediately reverse upon improved conditions. This differs from quiescence, in which the organism can rapidly respond to cues to exit the dormant state. Aestivation and hibernation occur in dry and cold conditions, respectively. Growth/degrowth and ontogeny reversal are included among dormancy types as they represent dramatic responses to changes in metabolism, nutrient availability and environmental stressors that are reversible. Dormancy terms are defined as reported in the literature but see Strachan *et al*. (2015) (reference 82) for a discussion on variability in terminology used. The literature cited is not exhaustive but aims to provide an accurate representation of the types of dormancy present within and across phyla. Red font: high levels of confidence with respect to ASC involvement in dormancy processes. Orange font: middle levels of confidence. Black font: reported, but no level of confidence established. ASCs can be characterised as undifferentiated (U) or differentiated (D) cell types. ? = uncertain data.

| **Taxon** | **Species** | **Dormancy type** | **Stage** | **Phenomenon**  **and cue** | **Cellular contribution** | | | | | | | **Cellular process: dedifferentiation, transdifferentiation** | **Reference** |
| --- | --- | --- | --- | --- | --- | --- | --- | --- | --- | --- | --- | --- | --- |
|  |  |  |  |  | **Cell types** | | **Origin** | | **‘Stemness’ gene expression** | **putative ASC or progenitor cells?** | **Potency** |  |  |
| **PORIFERA** |  |  |  |  |  | |  | |  |  |  |  |  |
| Demospongiae | *Asbestopluma hypogea* | growth/  degrowth | adult | starvation/  feeding cycles | unknown | | unknown | | unknown | unknown | unknown | unknown | 1 |
|  | *Ephydatia fluviatilis* | diapause | adult | gemmule diapause | archaeocytes, choanocytes | | U, D | | *Msi*, *Piwi* | yes = archaeocytes, choanocytes | totipotent, pluripotent | unknown | 2, 3, 85 |
|  | various freshwater species | diapause | adult | gemmule diapause/  aestivation | thesocytes | | D? | | unknown | yes = thesocytes? | unknown | unknown | 4 |
|  | *Spongilla lacustris*, *Corvomeyenia carolinensis*, *Ephydatia fluviatilis* | growth/  degrowth? | adult | Stress induced ‘reduction bodies’ | archaeocytes | | U | | unknown | unknown | unknown | unknown | 86–89 |
| Calcarea | *Petrobiona massiliana* | growth/  degrowth? | adult | Stress induced ‘dormant bodies’ | archaeocytes? | | U? | | unknown | unknown | unknown | unknown | 89, 90 |
| **PLACOZOA** |  |  |  |  |  | |  | |  |  |  |  |  |
|  |  | no dormancy |  |  |  | |  | |  |  |  |  | 5 |
| **CNIDARIA** |  |  |  |  |  | |  | |  |  |  |  |  |
| Hydrozoa | *Hydra vulgaris*, *H. magnipapillata*, *H. oligactis* | quiescence | adult | ‘polyp bail out’ (starvation) | interstitial *versus* epithelial cycling stem cells | | U, D | | unknown | yes = i-cells, epithelial stem cells? | combination multi/ totipotent and restricted | unknown | 6, 7 |
|  | *Hydra attenuata* | growth/degrowth | adult | growth/  degrowth (starvation) | unclear | | unknown | | unknown | unknown | unknown | unknown | 8 |
|  | *Turritopsis dohrnii*,  *T. nutricula* | ontogeny reversal | adult | ontogeny reversal after starvation, mechanical damage, deterioration of environmental conditions –reversion from adult back to more juvenile stage (post larval polyp); cyst stage | requires presence of differentiated cells of the exumbrellar epidermis and part of the gastrovascular system (endoderm lining); i-cells from tentacular bulb or manubrium required but not sufficient so differentiated cells also required | | U, D | | unknown | unknown | unclear but unlikely to be totipotent or to rely exclusively on stem cells | transdifferentation assumed. Few interstitial cells. Epidermis seems to give rise to epidermis and endoderm. No real demonstration that it is not dedifferentiation/ redifferentiation occurring either, but not likely to depend entirely on stem cells | 9–13 |
|  | *Laodicia undulata* | ontogeny reversal | medusa | reverse development medusa to polyp (starvation?); also creeping stolon (starvation) | unknown | | unknown | | unknown | unknown | unknown | unknown | 14, 15 |
|  | *Eudendrium*, *Sarsia*, *Tubularia*, *Halocordyle* | quiescence | adult | stolon as encystment structure; creeping hydrorhiza | unknown | | unknown | | unknown | unknown | unknown | unknown | 11 |
|  | *Craspedacusta* sp. | quiescence | eggs, larvae, polyps (species dependent) | cysts; dehydration and bleach | unknown | | unknown | | unknown | unknown | unknown | unknown | 11 |
|  | *Hydractinia* (*Podocoryne*) *carnea* | ontogeny reversal | medusa | reverse development under stress into ‘ball stage’ (ontogeny reversal)-only in young medusa buds | unknown | | unknown | | unknown | unknown | unknown | unknown | 16 |
| Hexacorallia | *Pocillopora damicornis* | ontogeny reversal | adult (polyp) | ‘reversible metamorphosis’ or ‘polyp bail-out’ (environmental stress): polyps leave hard skeleton and detach from parent colony, with regression to planula like stage | unknown | | unknown | | unknown | unknown | unknown | unknown | 17, 18 |
| Octocorallia | *Cornularia cornucopiae* | quiescence | adult (polyp) | hibernation; dormant stolon in winter; aestivation in some species | unknown | | unknown | | unknown | unknown | unknown | unknown | 19 |
| Cubozoa |  | no dormancy |  |  |  | |  | |  |  |  |  | 18, 20 |
| Scyphozoa | *Aurelia aurita*, *Aurelia* sp. | ontogeny reversal | medusa | Ontogeny reversal; degenerating juvenile medusae (overcrowding/ starvation) undergo ‘degrowth’ or ‘morphoretrogression’; also ‘podocysts’ | unknown | | unknown | | unknown | unknown | unknown | unknown | 16, 20, 21 |
|  | *Aurelia aurita* | growth/ degrowth | ephyra | growth/  degrowth (starvation) | unknown | | unknown | | unknown | unknown | unknown | unknown | 22 |
|  | *Chrysaora pacifica*, *Cyanea nozakii* | quiescence/ diapause | adult (polyp) | encystment; podocysts (seasonal or environmental) | unknown | | unknown | | unknown | unknown | unknown | unknown | 5, 23 |
|  | *Chrysaora hysoscella* | ontogeny reversal | ephyra | reverse development; limited: early ephyrae to scyphistoma | unknown | | unknown | | unknown | unknown | unknown | unknown | 18 |
| Staurozoa |  | no dormancy |  |  |  | |  | |  |  |  |  | 18, 20 |
| **CTENOPHORA** |  |  |  |  |  | |  | |  |  |  |  |  |
|  |  | no dormancy |  |  |  | |  | |  |  |  |  | 5 |
| **ACOELOMORPHA** |  |  |  |  |  | |  | |  |  |  |  |  |
|  | *Isodiometra pulchra* | growth/ degrowth | adult | growth/  degrowth (starvation) | unknown | | U | | *piwi* | yes = neoblasts | pluripotent/ totipotent | unknown | 24 |
| **PLATYHELMINTHES** |  |  |  |  |  | |  | |  |  |  |  |  |
| Macrostomorpha | *Macrostomum* sp., *Macrostomum lignano* | growth/ degrowth | adult | growth/  degrowth (starvation) | unknown | | unknown | | unknown | yes =neoblasts? | unknown | unknown | 25 |
|  | *Macrostomum orthostylum* | quiescence? | resting eggs | (salinity) | unknown | | unknown | | unknown | unknown | unknown | unknown | 20 |
| Tricladida | *Schmidtea mediterranea* | growth/ degrowth | adult | growth/  degrowth (starvation) | neoblast remains constant, with changes in differentiated populations | | U | | *piwi* | yes = neoblasts | assumed pluri/ totipotent | unknown | 26–28 |
|  | *Dugesia japonica* | growth/ degrowth | adult | growth/  degrowth (starvation) | neoblast | | U | | unknown | yes = neoblasts | assumed pluri/ totipotent | unknown | 29 |
| **NEMERTEA** |  |  |  |  |  | |  | |  |  |  |  |  |
|  | Various species | growth/ degrowth | adult | growth/  degrowth | unknown | | unknown | | unknown | unknown | unknown | unknown | 30–32 |
| Enopla |  | quiescence | adult | encystment (mucous cocoons) | unknown | | unknown | | unknown | unknown | unknown | unknown | 20 |
| **ANNELIDA** |  |  |  |  |  | |  | |  |  |  |  |  |
| Sedentaria | *Pristina leidyi* | quiescence | adult | post-starvation refeeding – gonad development | unknown | | unknown | | *piwi* | unknown | unknown | unknown | 33 |
|  | Naidids, *Lumbriculus* | growth/ degrowth | adult | growth/  degrowth  (starvation) | unknown | | unknown | | unknown | unknown | unknown | unknown | 32 |
|  | *Arenicola marina* | quiescence | adult | seasonal cold acclimation | unknown | | unknown | | unknown | unknown | unknown | unknown | 34 |
|  | *Aulodrilus acutus* | quiescence | adult | encystment; mucous cocoons (desiccation) | unknown | | unknown | | unknown | unknown | unknown | unknown | 35 |
|  | *Stylaria lacustris* | diapause | adult | overwintering cocoon | unknown | | unknown | | unknown | unknown | unknown | unknown | 36 |
|  | *Tubifex tubifex* | quiescence | adult | encystment (desiccation, starvation) | unknown | | unknown | | unknown | unknown | unknown | unknown | 37 |
| Clitellata | Leech *Hirudinea* | dormancy | larvae, adult | aestivation  anhydrobiosis | unknown | | unknown | | unknown | unknown | unknown | unknown | 82 |
| **BRACHIOPODA** |  |  |  |  |  | |  | |  |  |  |  |  |
|  |  | no dormancy |  |  |  | |  | |  |  |  |  |  |
| **PHORONIDA** |  |  |  |  |  | |  | |  |  |  |  |  |
|  |  | quiescence | adult | degenerate fragments in tubes (environmental stress) | unknown | | unknown | | unknown | unknown | unknown | unknown | 20 |
| **ECTOPROCTA** |  |  |  |  |  | |  | |  |  |  |  |  |
|  | various | quiescence | degenerating adult colony; eggs? | statoblasts (sessoblasts/  floatoblasts) –freezing/  desiccation | unknown | | unknown | | unknown | unknown | unknown | unknown | 38 |
| Phylactolaemata | *Lophopodella carteri* | quiescence | within body cavity of zooids | statoblasts (encapsulated dormant buds: sessoblasts, floatoblasts, piptoblasts) | unknown | | unknown | | unknown | unknown | unknown | unknown | 20 |
| Gymnolaemata | *Bulbella abscondita* | quiescence | adult | unspecialised quiescent stolons | unknown | | unknown | |  | unknown | unknown | unknown | 20, 39 |
| **GASTROTRICHA** |  |  |  |  |  | |  | |  |  |  |  |  |
| Chaetonotida | various | quiescence | resting eggs | crowding, freezing, desiccation | unknown | | unknown | | unknown | unknown | unknown | unknown | 20 |
| **MOLLUSCA** |  |  |  |  |  | |  | |  |  |  |  |  |
| Gastropoda | *Pila globosa* | quiescence | adult | aestivation | unclear; ASCs in salivary glands at base of alveoli in active state associated with basement membrane | | D? | | unknown | unknown | unknown | unknown | 40 |
|  | *Theba pisana* | quiescence | adult | aestivation | unclear; CNS | | unknown | | unknown | unknown | unknown | unknown | 41 |
|  | *Helix pomatia* | quiescence | adult | hibernation | unclear; brain and haemolymph | | unknown | | unknown | unknown | unknown | unknown | 42 |
| Bivalvia | *Laternula elliptica*, *Arctica islandica* | quiescence | adult | hibernation (cold, hypoxia, anoxia) | unknown | | unknown | | unknown | unknown | unknown | unknown | 20, 43, 44 |
|  | various | diapause | larvae, adult | aestivation | unknown | | unknown | | unknown | unknown | unknown | unknown | 82 |
| **ENTOPROCTA** |  |  |  |  |  | |  | |  |  |  |  |  |
|  | *Urnatella gracilis*, *Loxosomatoides sirindhornae*, *Barentsia matsuhimana* | quiescence/  diapause | adult |  | unknown | | unknown | | unknown | unknown | unknown | unknown | 5 |
| **CHAETOGNATHA** |  |  |  |  |  | |  | |  |  |  |  |  |
|  |  | no dormancy |  |  |  | |  | |  |  |  |  | 5 |
| **ROTIFERA** |  |  |  |  |  | |  | |  |  |  |  |  |
| Bdelloidea | *Mniobia* | quiescence | adult female/ tun | anhydrobiosis reversible desiccation/  ‘resuscitation’; extreme temperatures | unknown | | unknown | | unknown | unknown | unknown | unknown | 5, 20, 45 |
| Monogononta | *Synchaeta pectinata* | dormancy | resting eggs | desiccation | | unknown | | unknown | unknown | unknown | unknown | unknown | 5, 20 |
| **ARTHROPODA** |  |  |  |  |  | |  | |  |  |  |  |  |
|  | Various species across subphyla | diapause, quiescence | egg to adult | aestivation, hibernation, anydrobiosis, cryptobiosis | unknown | | unknown | | unknown | unknown | unknown | unknown | 82 |
| Crustacea | Calanid copepod *Neocalanus flemingeri* | diapause | adult | diapause (starvation); associated directly with reproduction | unknown | | unknown | | *piwi*, *ago* etc; | unknown | unknown | unknown | 46 |
|  | Brine shrimp *Artemia* sp. | diapause | embryo | encystment | unknown | | unknown | | unknown | unknown | unknown | unknown | 45, 81 |
|  | water fleas *Daphnia* spp. | diapause  quiescence | egg, adult | aestivation, hibernation | unknown | | unknown | | unknown | unknown | unknown | unknown | 82 |
|  | Various ostracods,  *Heterocypris bosniaca* | diapause  quiescence | egg, larvae, adult | hibernation | unknown | | unknown | | unknown | unknown | unknown | unknown | 82 |
| Hexapoda | African chironomid *Polypedilum vanderplanki* | quiescence | larvae | anhydrobiosis | unknown | | unknown | | unknown | unknown | unknown | unknown | 45 |
|  | *Drosophila* sp., *Anopheles*, *Sarcophaga* | diapause | adult, pupae, larvae | daylength/cold | unknown | | unknown | | unknown | unknown | unknown | unknown | 81 |
|  | *Collembola* | quiescence | egg, larvae, adult | cryptobiosis | unknown | | unknown | | unknown | unknown | unknown | unknown | 82 |
| Chelicerata | various mites Acarina | quiescence  diapause | adult | hibernation,  aestivation | unknown | | unknown | | unknown | unknown | unknown | unknown | 82 |
| **ONYCHOPHORA** |  |  |  |  |  | |  | |  |  |  |  |  |
|  | several  Peripatidae spp. | diapause | adult | reproductive diapause | unknown | | unknown | | unknown | unknown | unknown | unknown | 20, 47 |
| **TARDIGRADA** |  |  |  |  |  | |  | |  |  |  |  |  |
| Heterotradigrada | *Echiniscoides sigismundi* | quiescence | adult | cryptobiosis; desiccation tolerance (dormant state)-tun formation and recovery; anhydrobiosis very common and any stage of lifecycle | unknown | | unknown | | unknown | unknown | unknown | unknown | 45, 48 |
| Eutardigrada | *Richtersius coronifer* | quiescence | adult | cryptobiosis; desiccation tolerance (dormant state); anhydrobiosis; tun formation | Possible if Type II storage cells (categorised as coelomocytes) in females are stem cells; not affected in tuns | | U? | | unknown | unknown | unknown | unknown | 49 |
|  | *Thulinius ruffoi* | diapause | adult | diapause (temperature); encystment | unknown | | unknown | | unknown | unknown | unknown | unknown | 50 |
|  | various species | diapause | resting eggs | unknown | unknown | | unknown | | unknown | unknown | unknown | unknown | 20, 51, 52 |
|  | various species | quiescence | eggs | unknown | unknown | | unknown | | unknown | unknown | unknown | unknown | 52 |
| **NEMATODA** |  |  |  |  |  | |  | |  |  |  |  |  |
| Rhabidita: Secernentea | *Caenorhabditis elegans* | diapause | post-hatch larvae | nutrition caused L1 arrest or diapause | neural and mesodermal progenitors | | D? | | unknown | no (progenitors) | unknown | stem and progenitors exit from quiescence | 20, 45, 53, 54, 81 |
|  | *Caenorhabditis elegans* | quiescence | any stage | anhydrobiosis; also resting eggs and desiccation-tolerant ‘dauer larva’ | unknown | | unknown | | unknown | unknown | unknown | unknown | 20, 81 |
|  | *Panagrolaimus davidi* | quiescence | larvae,  adult | anhydrobiosis (freezing) | unknown | | unknown | | unknown | unknown | unknown | unknown | 55 |
| **PRIAPULIDA** |  |  |  |  |  | |  | |  |  |  |  |  |
|  |  | no dormancy |  |  |  | |  | |  |  |  |  | 5 |
| **VERTEBRATA** |  |  |  |  |  | |  | |  |  |  |  |  |
| Mammalia | *Mus musculus* | diapause | embryo | diapause | embryonic stem cells | | U | | *myc* | yes = embryonic stem cells | potency not affected by *myc* depletion, only dormancy | unknown | 56 |
|  |  | diapause | adult | various seasonal physiological rhythms | tanycytes = radial glial cells that persist as ASCs; nutrient sensors | | D | | unknown | yes = tanycytes? | unknown | unknown | 57, 83 |
|  |  | diapause | adult | nutrient/calorie restriction | reserve intestinal stem cells (injury-resistant pool) | | U | | unknown |  | unknown | unknown | 58 |
|  | *Mesocricetus auratus* | quiescence | adult | hibernation (seasonal torpor/arousal) | hypothalamic stem cells decrease proliferation of newborn neurons during hibernation | | D | | unknown |  | unknown | unknown | 59 |
|  | *Ictidomys tridecemlineatus* | quiescence | adult | hibernation (seasonal torpor/arousal) | Pax7^+^ (Paired box protein 7^+^) muscle satellite stem cells | | U | | unknown | yes = satellite-like stem cells | Activation/  possible proliferation of quiescent reserve stem cell pool | unknown | 60 |
|  |  | quiescence | adult | hibernation (seasonal torpor/arousal) | unclear (ASCs in brain); cycling between epithelial and mesenchymal phenotype in brain | | D | | *Sox* | unknown | unknown | unknown | 61 |
|  | *Marmota*  *himalayana* | quiescence | adult | hibernation (seasonal torpor/arousal) | unclear (ASCs in brain) | | D | | *Sox*, *myc* (high during torpor, low arousal) | unknown | pluripotent (but not demonstrated) | unknown | 62 |
|  | *Ursus arctos* | quiescence | adult | hibernation (seasonal torpor/arousal) | adipose stem cells (Mesenchymal stem cells like) | | U | | unknown | yes = adipose stem cells? | can differentiate into adipose as well as osteogenic and chondrogenic cells in culture, so likely multi/  pluripotent | unknown | 63 |
|  | *Neovison vison* (American mink), *Macropus eugenii* (Tammar wallaby), *Tarsipus rostratus* (honey possum), *Dasypus novemcinctus*  (nine-banded armadillo) | diapause | embryo (blastocyst) | diapause | unknown | | unknown | | unknown | unknown | unknown | unknown | 64 |
| Amphibia | *Cyclorana alboguttata*; many species of frog and salamander; sirens | quiescence and growth/ degrowth | adult | aestivation (drought tolerance; cocoon) | unknown | | unknown | | unknown | unknown | unknown | unknown | 65, 91 |
| Reptilia | various: snakes, crocodiles | quiescence | adult | hibernation | unknown | | unknown | | unknown | unknown | unknown | unknown | 66, 91 |
|  | various viviparous lizards, turtles | diapause | embryo | diapause | unknown | | unknown | | unknown | unknown | unknown | unknown | 67, 91 |
|  | various lizards, snakes, crocodilians, turtles | quiescence and growth/ degrowth | adult | aestivation (and laboratory-induced drying and fasting experiments) | unknown | | unknown | | unknown | unknown | unknown | unknown | 91 |
| Chondrychthys | *Rhizoprionodon taylori*, *Rhinobatus horkelli*, *Dasyatus say* | diapause | embryo (blastodisc) | diapause | unknown | | unknown | | unknown | unknown | unknown | unknown | 68 |
| Osteichthys-Actenopterygii | *Austrofundulus limnaeus* | diapause | embryo | diapause (drought) | unknown | | unknown | | *Pou* | unknown | unknown | unknown | 69 |
|  | *Nothobranchius furzeri* | diapause | resting eggs | diapause | unknown | | unknown | | *Pou*, *Polycomb* complex (e.g. *EZH1*, *CBX7*, *PCGF*); VASA protein | unknown | unknown | unknown | 70 |
|  | *Notothenia coriiceps* | quiescence | adult | hibernation | unknown | | unknown | | unknown | unknown | unknown | unknown | 5 |
|  | various including killifish *Austrofundulus limnaeus* | diapause | embryo | diapause | unknown | | unknown | | unknown | unknown | unknown | unknown | 71 |
|  | *Brachyhypopomus bombilla* | quiescence | adult | dormancy | unknown | | unknown | | unknown | unknown | unknown | unknown | 73 |
|  | *Synbranchus marmoratus* | quiescence | adult | aestivation | unknown | | unknown | | unknown | unknown | unknown | unknown | 74, 91 |
| Osteichthys-Sarcopterygi | Lungfishes *Protopterus aethiopicus*,  *P. dolloi*,  *P annectens*  *Lepidosiren paradoxia*  *Neoceratodus forsteri* | quiescence and growth/ degrowth | adult | aestivation (some within cocoon) | unknown | | unknown | | unknown | unknown | unknown | unknown | 72, 91 |
| **CEPHALOCHORDATA** |  |  |  |  |  | |  | |  |  |  |  |  |
|  |  | no dormancy |  |  |  | |  | |  |  |  |  | 5, 20 |
| **XENOTURBELLIDA** |  |  |  |  |  | |  | |  |  |  |  |  |
|  |  | no dormancy |  |  |  | |  | |  |  |  |  | 5 |
| **ECHINODERMATA** |  |  |  |  |  | |  | |  |  |  |  |  |
| Holothuroidea | *Apostichopus japonicus* | quiescence | adult | aestivation (high temperature) | unknown | | D? | | *klf* | unknown | unknown | unknown | 5, 78 |
| Echinoidea | *Strongylocentrotus droebachiensis* | quiescence | Juvenile  /larva | rudiment resorption (starvation) | unknown | | unknown | | unknown | unknown | unknown | unknown | 79 |
| Crinoidea |  | no dormancy |  |  |  | |  | |  |  |  |  | 20 |
| Asteroidea |  | no dormancy |  |  |  | |  | |  |  |  |  | 20 |
| **HEMICHORDATA** |  |  |  |  |  | |  | |  |  |  |  |  |
| Pterobranchia |  | quiescence | buds | dormant asexual buds | unknown | | unknown | | unknown | unknown | unknown | unknown | 20 |
| Enteropneusta |  | no dormancy |  |  |  | |  | |  |  |  |  | 20 |
| **UROCHORDATA** |  |  |  |  |  | |  | |  |  |  |  |  |
| Ascidiascea | *Botrylloides leachi* | quiescence | adult | colony regression/ aestivation/ hibernation/ torpor | Yes; activation/ transient mobilisation of piwi+ cells lining vasculature during environmental stress | | U/D | | *piwi*, *pl10* (high in MNC) | yes = inner cells? | multi/ totipotent | dormant stem cells that are attached to the vasculature epithelium may be the source of new stem cells or new blood cell population through process of dedifferentiation as in WBR | 75, 76, 84 |
|  | *Botryllus schlosseri* | diapause | eggs | unknown | unknown | | unknown | | unknown | unknown | unknown | unknown | 5 |
|  | *Polyandrocarpa zorritensis* | quiescence | adult | unknown | unknown | | budding niche | | unknown | unknown | unknown | unknown | 80 |
|  | various | various | various | multiple | unknown | | unknown | | unknown | unknown | unknown | unknown | 77 |
| Thaliacea |  | no dormancy |  |  |  | |  | |  |  |  |  | 20 |
| Appendicularia |  | no dormancy |  |  |  | |  | |  |  |  |  | 20 |

MNC, multi-nucleated cells; Piwi, P-element induced wimpy testis; Vasa protein, an RNA binding protein with an ATP-dependent RNA helicase, a member of the DEAD box family of proteins; WBR, whole body regeneration.

**References**

1. Martinand-Mari C, Vacelet J, Nickel M, Wörheide G, Mangeat P, Baghdiguian S. 2012. Cell death and renewal during prey capture and digestion in the carnivorous sponge *Asbestopluma hypogea* (Porifera: Poecilosclerida). J Exp Biol. 215:3937-43.
2. Funayama N, Nakatsukasa M, Mohri K, Masuda Y, Agata K. 2010. Piwi expression in archeocytes and choanocytes in demosponges: insights into the stem cell system in demosponges. Evol Dev. 12:275-87.
3. Okamoto K, Nakatsukasa M, Alié A, Masuda Y, Agata K, Funayama N. 2012. The active stem cell specific expression of sponge Musashi homolog EflMsiA suggests its involvement in maintaining the stem cell state. Mech Dev. 129:24-37.
4. Loomis SH. 2010. Diapause and estivation in sponges. Prog Mol Subcell Biol. 49:231-43.
5. García-Roger EM, Lubzens E, Fontaneto D, Serra M. 2019. Facing adversity: Dormant embryos in rotifers. Biol Bull. 237:119-144.
6. Buzgariu W, Crescenzi M, Galliot B. 2014. Robust G2 pausing of adult stem cells in *Hydra.* Differentiation. 87:83-99.
7. Bosch TC, David CN. 1984. Growth regulation in *Hydra*: relationship between epithelial cell cycle length and growth rate. Dev Biol.104(1):161-71.
8. Otto JJ, Campbell RD. 1977. Tissue economics of *Hydra*: regulation of cell cycle, animal size and development by controlled feeding rates. J Cell Sci. 28:117‐132.
9. Bavestrello G, Sommer C, Sarà M. 1992. Bidirectional conversion in *Turritopsis nutricula* (Hydrozoa). Scientia Marina. 56:137-140.
10. Piraino S, Boero F, Aeschbach B, Schmid V. 1996. Reversing the life cycle: Medusae transforming into polyps and cell transdifferentiation in *Turritopsis nutricula* (Cnidaria, Hydrozoa). Biol Bull. 190(3):302-312.
11. Boero F, Bouillon J, Piraino S, Schmid V. 2002. Asexual reproduction in the Hydrozoa (Cnidaria) In: Hughes RN, editor. Reproductive Biology of Invertebrates. XI Progress in Asexual Reproduction. New Delhi & Kolkata: Oxford & IBH Publishing Co. p. 141-58.
12. Carla EC, Pagliara P, Piraino S, Boero F, Dini L. 2003. Morphological and ultrastructural analysis of *Turritopsis nutricula* during life cycle reversal. Tissue Cell. 35(3):213-22.
13. Matsumoto, Y., Piraino, S., & Miglietta, M. P. 2019. Transcriptome characterization of reverse development in *Turritopsis dohrnii* (Hydrozoa, Cnidaria). G3 (Bethesda, Md.). 9(12):4127-4138.
14. De Vito D, Piraino S, Schmich J, Bouillon J, Boero F. 2006. Evidence of reverse development in Leptomedusae (Cnidaria, Hydrozoa): the case of *Laodicea undulata* (Forbes and Goodsir 1851). Mar Biol. 149(2):339-46.
15. Kubota S. 2006. Life cycle reversion of *Laodicea undulata* (Hydrozoa, Leptomedusae) from Japan. Bull Biogeogr Soc Jpn. 61(1): 85-8.
16. Schmich J, Kraus Y, De Vito D, Graziussi D, Boero F, Piraino S. 2007. Induction of reverse development in two marine hydrozoans. Int J Dev Biol. 51(1):45-56.
17. Sammarco PW. 1982. Polyp bail-out: an escape response to environmental stress and a new means of reproduction in corals. Mar Ecol. 10:57-65.
18. Piraino S, De Vito D, Schmich J, Bouillon J and Boero F. 2004. Reverse development in Cnidaria. Can J Zool. 82(11):1748-1754.
19. Betti, F, Bo M, Di Camillo CG, Bavestrello G. 2012. Life history of *Cornularia cornucopiae* (Anthozoa: Octocorallia) on the Conero promontory (north Adriatic Sea). Mar Ecol. 33(1):49-55.
20. Càceres CE. 1997. Dormancy in invertebrates. Invertebr Biol. 116:371-383.
21. He J, Zheng L, Zhang W, Lin Y. 2015. Life cycle reversal in *Aurelia sp.1* (Cnidaria, Scyphozoa). PLoS ONE. 10(12):e0145314.
22. Fu Z, Shibata M, Makabe R, Ikeda H, Uye SI. 2014. Body size reduction under starvation, and the point of no return, in ephyrae of the moon jellyfish *Aurelia aurita*. Mar Ecol. 510:255-263.
23. Thein H, Ikeda H, Uye SI. 2013. Ecophysiological characteristics of podocysts in *Chrysaora pacifica* (Goette) and *Cyanea nozakii* Kishinouye (Cnidaria: Scyphozoa: Semaeostomeae): effects of environmental factors on their production, dormancy and excystment. J Exp Mar Biol Ecol. 446:151-158.
24. De Mulder K, Kuales G, Pfister D, Willems M, Egger B, Salvenmoser W, Thaler M, Gorny AK, Hrouda M, Borgonie G, Ladurner P. 2009*a*. Characterization of the stem cell system of the acoel *Isodiametra pulchra*. BMC Dev Biol. 9:69.
25. Nimeth KT, Mahlknecht M, Mezzanato A, Peter R, Rieger R, Ladurner P. 2004. Stem cell dynamics during growth, feeding, and starvation in the basal flatworm *Macrostomum sp* (Platyhelminthes). Dev Dyn. 230(1):91-9.
26. Peiris TH, Weckerle F, Ozamoto E, Ramirez D, Davidian D, García-Ojeda ME, Oviedo NJ. 2012. TOR signaling regulates planarian stem cells and controls localized and organismal growth. J Cell Sci. 125(Pt 7):1657-65.
27. González-Estévez C, Felix DA, Rodríguez-Esteban G, Aboobaker AA. 2012. Decreased neoblast progeny and increased cell death during starvation-induced planarian degrowth. Int J Dev Biol. 56(1-3):83-91.
28. Felix DA, Gutiérrez-Gutiérrez Ó, Espada L, Thems A, González-Estévez C. 2019. It is not all about regeneration: planarians striking power to stand starvation. Semin Cell Dev Biol. 87:169-181.
29. Sakurai T, Lee H, Kashima M, Saito Y, Hayashi T, Kudome-Takamatsu T, Nishimura O, Agata K, Shibata N. 2012. The planarian P2X homolog in the regulation of asexual reproduction. Int J Dev Biol. 56(1-3):173-82.
30. Dawydoff MC. 1928. Sur la réversibilité des processus du développement. Les phases extrêmes de la réduction des Némertes. C R Hebd Seanc Acad Sci. 186:911-913.
31. Coe WR. 1929. Regeneration in nemerteans. J Exp Zool. 54:411-459.
32. Bely AE, Zattara EE, Sikes JM. 2014. Regeneration in spiralians: evolutionary patterns and developmental processes. Int J Dev Biol. 58(6-8):623-34.
33. Özpolat BD, Sloane ES, Zattara EE, Bely AE. 2016. Plasticity and regeneration of gonads in the annelid *Pristina leidyi*. Evodevo. 7:22.
34. Sommer AM, Pörtner HO. 2004. Mitochondrial function in seasonal acclimatization versus latitudinal adaptation to cold in the lugworm *Arenicola marina* (L.). Physiol Biocheml Zool. 77(2):174-186.
35. Ohtaka A. 2018. Aquatic oligochaete fauna (Annelida, Clitellata) in Lake Tonle Sap and adjacent waters in Cambodia. Limnology. 19(3):367-373.
36. Schierwater B, Hauenschild C. 1990. A photoperiod determined life-cycle in an oligochaete worm. Biol Bull. 178(2):111-117.
37. Anlauf A. 1990. Cyst formation of *Tubifex tubifex* (Müller)-an adaptation to survive food deficiency and drought. Hydrobiologia. 190(1):79-82.
38. Hengherr S, Schill RO. 2011. Dormant stages in freshwater bryozoans-an adaptation to transcend environmental constraints. J Insect Physiol*.* 57(5):595-601.
39. Rogick MD. 1941. The resistance of fresh-water Bryozoa to desiccation. Biodynamica. 3:369-378.
40. Bhattacharyya KN, Chaki KK, Sarkar AK, Misra KK. 2012. Ultrastructure of the salivary gland cells in active and aestivated mollusk, *Pila* *globosa* (Gastropoda: Orthogastropoda: Ampularidae). Proc Zool Soc. 65:64-69.
41. Adamson KJ, Wang T, Rotgans BA, Kruangkum T, Kuballa AV, Storey KB, Cummins SF. 2017. Genes and associated peptides involved with aestivation in a land snail. Gen Comp Endocrinol. 246:88-98
42. Pirger Z, Lubics A, Reglodi D, Laszlo Z, Mark L, Kiss T. 2010. Mass spectrometric analysis of activity-dependent changes of neuropeptide profile in the snail, *Helix pomatia*. Neuropeptides. 44(6):475-83.
43. Brockington S. 2001. The seasonal energetics of the Antarctic bivalve *Laternula elliptica* (King and Broderip) at Rothera point, Adelaide Island. Polar Biol*.* 24(7):523-530.
44. Philipp EE, Wessels W, Gruber H, Strahl J, Wagner AE, Ernst IM, Rimbach G, Kraemer L, Schreiber S, Abele D, Rosenstiel P. 2012. Gene expression and physiological changes of different populations of the long-lived bivalve *Arctica islandica* under low oxygen conditions. PLoS ONE. 7(9):e44621.
45. Rebecchi L, Altiero T, Guidetti R. 2007. Anhydrobiosis: the extreme limit of dessication tolerance. Invertebr Surv J. 4(2):65-81.
46. Roncalli V, Sommer SA, Cieslak MC, Clarke C, Hopcroft RR, Lenz PH. 2018. Physiological characterization of the emergence from diapause: A transcriptomics approach. Sci Rep. 8(1):12577.
47. Monge-Nájera J. 1994. Ecological biogeography in the phylum Onycophora. Biogeographica.70:111-123.
48. Hygum TL, Clausen LKB, Halberg KA, Jørgensen A, Møbjerg N. 2016. Tun formation is not a prerequisite for desiccation tolerance in the marine tidal tardigrade *Echiniscoides sigismundi.* Zool J Linnean Soc. 178:907-911.
49. Czerneková M, Janelt K, Student S, Jönsson KI, Poprawa I. 2018. A comparative ultrastructure study of storage cells in the eutardigrade *Richtersius coronifer* in the hydrated state and after desiccation and heating stress. PLoS ONE. 13(8):e0201430.
50. Janelt K, Poprawa I. 2020. Analysis of encystment, excystment, and cyst structure in freshwater Eutardigrade *Thulinius ruffoi* (Tardigrada, Isohypsibioidea: Doryphoribiidae). Diversity. 12:62.
51. Kinchin IM. 1994. The Biology of Tardigrades. London: Portland Press.
52. Guidetti R, Altiero T, Rebecchi L. 2011. On dormancy strategies in tardigrades. J Insect Physiol. 57(5):567-576.
53. Padilla PA, Ladage ML. 2012. Suspended animation, diapause and quiescence. Cell Cycle. 11(9):1672-9.
54. Fukuyama M, Kontani K, Katada T, Rougvie AE. 2015. The *C. elegans* hypodermis couples progenitor cell quiescence to the dietary state. Curr Biol. 25(9):1241-8.
55. Lewis SC, Dyal LA, Hilburn, C.F. Weitz S, Liau WS, LaMunyon CW, Denver DR*.* 2009. Molecular evolution in *Panagrolaimus* nematodes: origins of parthenogenesis, hermaphroditism and the Antarctic species *P. davidi*. BMC Evol Biol 9:1-13.
56. Scognamiglio R, Cabezas-Wallscheid N, Thier MC, Altamura S, Reyes A, Prendergast ÁM, Baumgärtner D, Carnevalli LS, Atzberger A, Haas S, von Paleske L, Boroviak T, Wörsdörfer P, Essers MA, Kloz U, Eisenman RN, Edenhofer F, Bertone P, Huber W, van der Hoeven F, Smith A, Trumpp A. 2016. Myc depletion induces a pluripotent dormant state mimicking diapause. Cell. 164(4):668-80.
57. Lewis JE, Ebling FJ. 2017. Tanycytes as regulators of seasonal cycles in neuroendocrine function. Front Neurol. 8:79.
58. Yousefi M, Nakauka-Ddamba A, Berry CT, Li N, Schoenberger J, Simeonov KP, Cedeno RJ, Yu Z, Lengner CJ. 2018. Calorie restriction governs intestinal epithelial regeneration through cell-autonomous regulation of mTORC1 in reserve stem cells. 10(3):703-711.
59. León-Espinosa G, Regalado-Reyes M, DeFelipe J, Muñoz A. 2018. Changes in neocortical and hippocampal microglial cells during hibernation. Brain Struct Funct. 223(4):1881-1895.
60. Brooks NE, Myburgh KH, Storey KB. 2015. Muscle satellite cells increase during hibernation in ground squirrels. Comp Biochem Physiol B Biochem Mol Biol. 189:55-61.
61. Lee YJ, Bernstock JD, Klimanis D, Hallenbeck JM. 2018. Akt Protein Kinase, miR-200/miR-182 expression and epithelial-mesenchymal transition proteins in hibernating ground squirrels. Front Mol Neurosci. 11:22.
62. Bai L, Liu B, Ji C, Zhao S, Liu S, Wang R, Wang W, Yao P, Li X, Fu X, Yu H, Liu M, Han F, Guan N, Liu H, Liu D, Tao Y, Wang Z, Yan S, Florant G, Butcher MT, Zhang J, Zheng H, Fan J, Liu E. 2019. Hypoxic and cold adaptation insights from the Himalayan marmot genome. iScience. 11:519-530.
63. Fink T, Rasmussen JG, Emmersen J, Pilgaard L, Fahlman Å, Brunberg S, Josefsson J, Arnemo JM, Zachar V, Swenson JE, Fröbert O. 2011. Adipose-derived stem cells from the brown bear (*Ursus arctos*) spontaneously undergo chondrogenic and osteogenic differentiation *in vitro*. Stem Cell Res. 7(1):89‐95.
64. Renfree MB, Fenelon JC. 2017. The enigma of embryonic diapause. Development. 144(18):3199-3210.
65. Reilly BD, Schlipalius DI, Cramp RL, Ebert PR & Franklin CE. 2013. Frogs and estivation: transcriptional insights into metabolism and cell survival in a natural model of extended muscle disuse. Physiol Genomics*.* 45(10):377-388.
66. Bennet AF. 1994. Exercise performance of reptiles. Adv Vet Sci Comp Med. 38B:113-38.
67. Ewert M. 1991. Cold torpor, diapause, delayed hatching and aestivation in reptiles and birds. In: Deeming DC, Ferguson MWJ, editors. Egg incubation: Its effects on embryonic development in birds and reptiles. Cambridge: Cambridge University Press. p. 173-192.
68. Waltrick D, Awruch C, Simpfendorfer C. 2012. Embryonic diapause in the elasmobranchs. Rev Fish Biol Fisher. 22(4):849-859.
69. Wagner JT, Podrabsky JE. 2015. Gene expression patterns that support novel developmental stress buffering in embryos of the annual killifish *Austrofundulus limnaeus*. Evodevo. 6:2.
70. Hu CK, Wang W, Brind'Amour J, Singh PP, Reeves GA, Lorincz MC, Alvarado AS, Brunet A. 2020. Vertebrate diapause preserves organisms long term through Polycomb complex members. Science. 367(6480):870‐874.
71. Schiesari L, O'Connor MB. 2013. Diapause: delaying the developmental clock in response to a changing environment. Curr Top Dev Biol. 105:213-46.
72. Ong JL, Chng YR, Ching B, Chen XL, Hiong KC, Wong WP, Chew SF, Ip YK. 2017. Molecular characterization of myostatin from the skeletal muscle of the African lungfish, *Protopterus annectens*, and changes in its mRNA and protein expression levels during three phases of aestivation. J Comp Phys B. 187(4):575-589.
73. Giora J, Tarasconi HM, Fialho CB. 2012. Reproduction and feeding habits of the highly seasonal *Brachyhypopomus bombilla* (Gymnotiformes: Hypopomidae) from southern Brazil with evidence for a dormancy period. Environ Biol Fishes. *94*(4):649-662.
74. Moraes G, Altran AE, Avilez IM, Barbosa CC, Bidinotto PM. 2005. Metabolic adjustments during semi-aestivation of the marble swamp eel (*Synbranchus marmoratus*, Bloch 1795)-a facultative air breathing fish. Braz J Biol. 65(2):305-312.
75. Rinkevich Y, Rosner A, Rabinowitz C, Lapidot Z, Moiseeva E, Rinkevich B. 2010. Piwi positive cells that line the vasculature epithelium, underlie whole body regeneration in a basal chordate. Dev Biol. 345(1):94-104.
76. Hyams Y, Paz G, Rabinowitz C, Rinkevich B. 2017. Insights into the unique torpor of *Botrylloides leachi*, a colonial urochordate Dev Biol. 428(1):101-117.
77. Berrill NJ. 1951. Regeneration and budding in tunicates. Biol Rev. 26:451–475.
78. Li Y, Wang R, Xun X, Wang J, Bao L, Thimmappa R, Ding J, Jiang J, Zhang L, Li T, Lv J, Mu C, Hu X, Zhang L, Liu J, Li Y, Yao L, Jiao W, Wang Y, Lian S, Zhao Z, Zhan Y, Huang X, Liao H, Wang J, Sun H, Mi X, Xia Y, Xing Q, Lu W, Osbourn A, Zhou Z, Chang Y, Bao Z, Wang S. 2018. Sea cucumber genome provides insights into saponin biosynthesis and aestivation regulation. Cell Discov. 4:29.
79. Singh A, Pinto L, Martin C, Rutherford N, Ragunathan A, Upadhyay U, Kapoor P, McRae M, Siddiqui A, Cantelmi D, Heyland A. 2018. Rudiment resorption as a response to starvation during larval development in the sea urchin *Strongylocentrotus* *droebachiensis*. Can J Zool. 96(10):1178-1185.
80. Scelzo M, Alié A, Pagnotta S, Lejeune C, Henry P, Gilletta L, Hiebert LS, Mastrototaro F, Tiozzo S. 2019. Novel budding mode in *Polyandrocarpa zorritensis*: a model for comparative studies on asexual development and whole body regeneration. Evodevo. 10:7.
81. Hand SC, Denlinger DL, Podrabsky JE, Roy R. 2016. Mechanisms of animal diapause: recent developments from nematodes, crustaceans, insects, and fish. Am J Physiol Regul Integr Comp Physiol. 310(11):R1193-211.
82. Strachan SR, Chester ET, Robson BJ. 2015. Freshwater invertebrate life history strategies for surviving desiccation. Springer Sci Rev. 3(1):57-75.
83. Ebling FJP, Lewis JE. 2018. Tanycytes and hypothalamic control of energy metabolism. Glia. 66(6):1176-1184.
84. Burighel P, Brunetti R, Zaniolo G. 1976. Hibernation of the colonial ascidian *Botrylloides leachi* (Savigny): histological observations. Ital J Zool (Modena). 43:293-301.
85. Höhr D. 1977. Differenzierungsvorgänge in der keimenden Gemmula von *Ephydatia fluviatilis.* Wilhelm Roux' Archiv. 182:329-346.
86. Bisbee JW, Francis JC and Harrison FW. 1989. Cytological examination of freshwater sponge regeneration from reduction bodies. Trans Am Microsc Soc. 108(3):299-303.
87. Harrison FW, Dunkelberger D, Watabe N. 1975 Cytological examination of reduction bodies of *Corvomeyenia carolinensis* harrison (Porifera: Spongillidae). J Morph. 145(4):483-491.
88. Harrison FW, Davis DA 1982. Morphological and cytochemical patterns during early stages of reduction body formation in *Spongilla lacustris* (Porifera: Spongillidae). Trans Am Microsc Soc. 101(4):317-324.
89. Gaino E, Manconi R, Pronzato R. 1995. Organizational plasticity as a successful conservative tactics in sponges. Anim Biol. 4: 31-43.
90. Vacelet J. 1990. Storage cells of calcified relict sponges. In: Ruetzler K, editor*.* New perspectives in sponge biology. Washington: Smithsonian Institution Press. p. 144-152.
91. Secor SM, Lignot JH. 2010. Morphological plasticity of vertebrate aestivation. In: Arturo Navas C, Carvalho J, editors. Aestivation. progress in molecular and subcellular biology. Vol 49. Springer, Berlin, Heidelberg. p. 183-208.
